# Supplementary material for: Implications of the availability and distribution of birth weight on addressing neonatal mortality: population-based assessment from Bihar state of India
Source: BMJ Open. 2022 Jun 21;12(6):e061934. doi: 10.1136/bmjopen-2022-061934 (PMC9214371; doi:10.1136/bmjopen-2022-061934)

Supplementary Figure 1. Distribution of birthweight values of 2500 g, 3,000 g, and 3,500 g by select variables among the livebirths born between October 2018 to September 2019 for whom birthweight was available in the Indian state of Bihar.

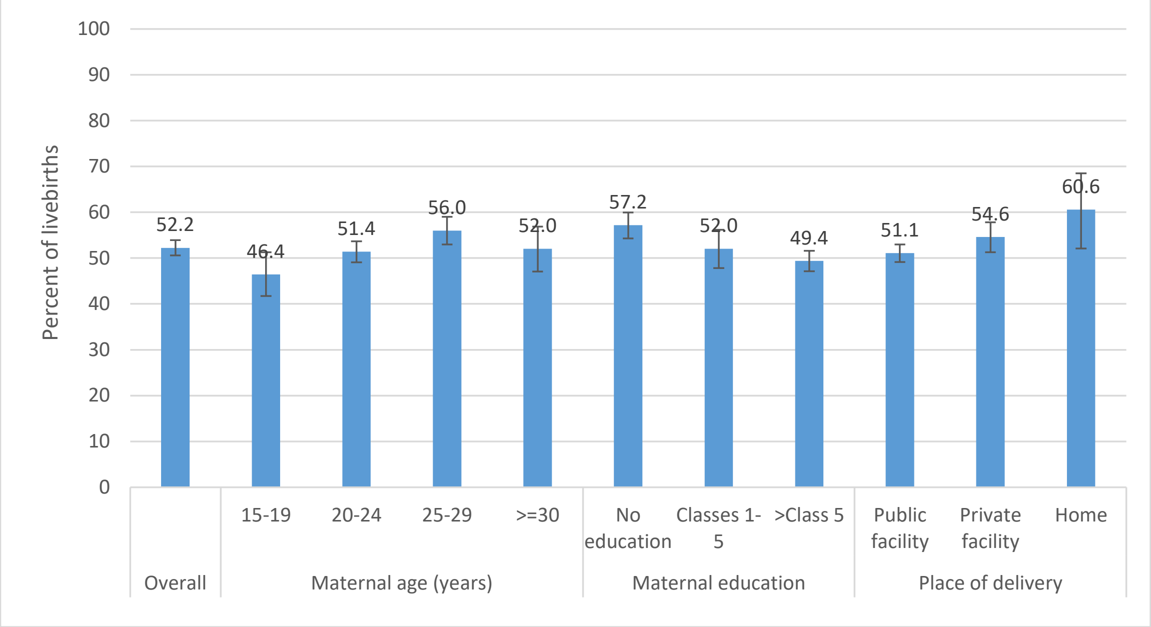

Supplement: Supplementary data [file bmjopen-2022-061934supp001.pdf]
