# Supplementary material for: Implications of the availability and distribution of birth weight on addressing neonatal mortality: population-based assessment from Bihar state of India
Source: BMJ Open. 2022 Jun 21;12(6):e061934. doi: 10.1136/bmjopen-2022-061934 (PMC9214371; doi:10.1136/bmjopen-2022-061934)
Supplement: Supplementary data [file bmjopen-2022-061934supp002.pdf]

Supplementary Table 1. Prevalence of birthweight by categories, of no recall, and of child not being weighted at birth for select characteristics in the Indian state of Bihar for livebirths between October 2018 to September 2019.

	Prevalence per 100 livebirths (95% confidence interval)						
	**Birthweight >=2,500 g**	**Birthweight <2,500 g**	***Birthweight 2,000 - 2,499 g***	***Birthweight 1,500 - 1,999 g***	***Birthweight <1,500 g***	**Mother could not recall birthweight**	**Child not weighted at birth**
Overall	59.3 (57.9-60.6)	13.3 (12.4-14.3)	*9.3 (8.5-10.1)*	*3.0 (2.6-3.5)*	*1.1 (0.8-1.4)*	5.8 (5.2-6.5)	21.5 (20.4-22.7)
**Maternal age***							
15-19 years	56.1 (51.9-60.3)	20.8 (17.5-24.5)	*15.3 (12.5-18.6)*	*4.7 (3.2-6.9)*	*0.8 (0.3-2.0)*	6.0 (4.3-8.4)	17.0 (14.0-20.5)
20-24 years	61.6 (59.6-63.5)	14.0 (12.7-15.5)	*9.8 (8.7-11.1)*	*3.1 (2.4-3.8)*	*1.1 (0.8-1.6)*	5.1 (4.3-6.1)	19.3 (17.7-20.9)
25-29 years	60.8 (58.3-63.3)	9.9 (8.5-11.6)	*6.5 (5.4-7.9)*	*2.3 (1.7-3.3)*	*1.0 (0.6-1.7)*	6.5 (5.4-7.9)	22.7 (20.6-24.9)
>=30 years	49.9 (46.0-53.8)	12.0 (9.7-14.8)	*8.5 (6.6-11.0)*	*2.7 (1.7-4.3)*	*0.8 (0.3-1.9)*	6.5 (4.8-8.7)	31.6 (28.1-35.3)
**Maternal education§**							
No education	48.3 (46.1-50.6)	13.1 (11.7-14.7)	*8.6 (7.4-9.9)*	*3.1 (2.5-4.0)*	*1.4 (0.9-2.0)*	8.0 (6.8-9.3)	30.6 (28.5-32.7)
Class 1 to 5	56.7 (53.2-60.2)	14.9 (12.5-17.6)	*10.4 (8.4-12.8)*	*3.7 (2.6-5.3)*	*0.8 (0.4-1.7)*	5.7 (4.2-7.5)	22.8 (19.9-25.9)
More than class 5	69.1 (67.2-70.9)	12.9 (11.6-14.4)	*9.5 (8.4-10.8)*	*2.6 (2.0-3.3)*	*0.8 (0.5-1.3)*	4.1 (3.4-5.0)	13.8 (12.5-15.3)
**Wealth index quartile #**							
I	47.6 (44.9-50.4)	14.3 (12.4-16.3)	*9.5 (8.0-11.2)*	*3.6 (2.7-4.8)*	*1.2 (0.7-2.0)*	6.1 (4.9-7.6)	32.0 (29.4-34.6)
II	54.3 (51.6-57.1)	14.3 (12.4-16.3)	*9.6 (8.1-11.4)*	*3.3 (2.5-4.5)*	*1.3 (0.8-2.1)*	7.3 (5.9-8.8)	24.1 (21.9-26.6)
III	62.0 (59.3-64.6)	13.3 (11.5-15.3)	*10.0 (8.4-11.7)*	*2.4 (1.7-3.4)*	*1.0 (0.5-1.7)*	7.0 (5.7-8.6)	17.7 (15.7-19.9)
IV	73.1 (70.6-75.5)	11.6 (9.9-13.4)	*8.1 (6.7-9.8)*	*2.6 (1.9-3.7)*	*0.8 (0.4-1.5)*	2.9 (2.1-4.0)	12.4 (10.7-14.4)
**Sex**							
Boy	62.3 (60.4-64.1)	11.9 (10.7-13.2)	*8.0 (7.0-9.1)*	*2.9 (2.4-3.7)*	*1.0 (0.6-1.4)*	5.6 (4.8-6.5)	20.2 (18.7-21.8)
Girl	56.0 (54.1-58.0)	14.9 (13.5-16.4)	*10.7 (9.5-12.0)*	*3.0 (2.4-3.8)*	*1.2 (0.8-1.7)*	6.1 (5.2-7.1)	23.0 (21.3-24.7)
**Gestation period§**							
6-7 months	8.7 (3.3-21.2)	63.0 (48.2-75.8)	*17.4 (8.9-31.3)*	*26.1 (15.4-40.7)*	*19.6 (10.4-33.7)*	8.7 (3.3-21.2)	19.6 (10.4-33.7)
8 months	55.4 (52.2-58.5)	18.9 (16.5-21.5)	*13.1 (11.1-15.4)*	*4.3 (3.2-5.8)*	*1.4 (0.8-2.4)*	5.4 (4.1-7.0)	20.3 (17.9-23.0)
>8 months	60.8 (59.3-62.3)	11.4 (10.5-12.4)	8.3 (7.5-9.2)	2.4 (2.0-2.9)	0.7 (0.5-1.0)	5.9 (5.2-6.7)	21.9 (20.6-23.2)
**Birth order**							
1st	63.5 (60.9-66.0)	17.8 (15.8-19.9)	12.5 (10.9-14.4)	4.2 (3.3-5.5)	1.0 (0.6-1.7)	5.1 (4.0-6.3)	13.7 (12.0-15.6)
2nd	62.1 (59.5-64.6)	12.3 (10.7-14.2)	8.9 (7.5-10.5)	2.4 (1.7-3.4)	1.0 (0.6-1.7)	5.8 (4.7-7.1)	19.8 (17.8-22.0)
$>2^{nd}$	55.2 (53.2-57.2)	11.2 (9.9-12.5)	7.6 (6.6-8.8)	2.5 (2.0-3.3)	1.0 (0.7-1.5)	6.3 (5.4-7.4)	27.3 (25.5-29.2)

	Prevalence per 100 livebirths (95% confidence interval)						
	**Birthweight >=2,500 g**	**Birthweight <2,500 g**	***Birthweight 2,000 - 2,499 g***	***Birthweight 1,500 - 1,999 g***	***Birthweight <1,500 g***	**Mother could not recall birthweight**	**Child not weighted at birth**
**Place of delivery**§							
Public sector facility	74.8 (73.2-76.4)	16.6 (15.2-18.0)	*12.1 (10.9-13.3)*	*3.3 (2.7-4.0)*	*1.2 (0.8-1.7)*	7.3 (6.4-8.3)	1.3 (1.0-1.8)
Private sector facility	71.1 (68.3-73.8)	15.9 (13.8-18.3)	*9.9 (8.2-11.9)*	*4.7 (3.6-6.2)*	*1.4 (0.8-2.3)*	7.1 (5.7-8.9)	5.8 (4.5-7.4)
Home	9.2 (7.6-11.0)	2.6 (1.8-3.7)	*1.7 (1.1-2.6)*	*0.6 (0.3-1.3)*	*0.3 (0.1-0.8)*	0.8 (0.4-1.5)	87.5 (85.4-89.3)
**Current status of livebirth**							
Died on day 0 of birth	28.1 (17.9-41.1)	17.5 (9.7-29.8)	*5.3 (1.7-15.2)*	*8.8 (3.7-19.5)*	*3.5 (0.9-13.1)*	14.0 (7.1-25.8)	40.4 (28.4-53.6)
Died between day 1-27 of birth	37.9 (26.4-51.1)	31.0 (20.4-44.1)	*12.1 (5.8-23.8)*	*12.1 (5.8-23.3)*	*6.9 (2.6-17.1)*	*12.1 (5.8-23.3)*	*19.0 (10.8-31.2)*
Died between day 28 and 11 months of age	37.1 (22.8-54.2)	25.7 (13.8-42.8)	*8.6 (2.7-23.8)*	*11.4 (4.3-27.1)*	*5.7 (1.4-20.5)*	*0*	*37.1 (22.8-54.2)*
Alive	60.1 (58.7-61.4)	13.0 (12.1-14.0)	*9.3 (8.5-10.2)*	*2.8 (2.3-3.2)*	*0.9 (0.7-1.2)*	5.7 (5.1-6.4)	21.2 (20.1-22.4)

*****Data not available for 14 livebirths
§Data not available for 4 livebirths
Data not available for 1 livebirth

Supplementary Table 2. Association of low birth weight (LBW) among babies with birth weight available with select variables using multiple logistic regression for livebirths between October 2018 to September 2019 in the Indian state of Bihar.

	<2,500 g birthweight (LBW)		
**Variables**	**Total N=3,647 (% of total)**	**% of livebirths with LBW**	**Odds ratio for having LBW (95% confidence interval)**
**Maternal age***			
15-19 years	407 (11.2)	110 (27.0)	1.00
20-24 years	1808 (49.7)	335 (18.5)	0.8 (0.6-1.0)
25-29 years	1028 (28.3)	144 (14.0)	0.5 (0.4-0.8)
>=30 years	392 (10.8)	76 (19.4)	0.7 (0.5-1.1)
**Maternal education**#†			
No education	1172 (32.2)	250 (21.3)	1.4 (1.1-1.8)
Classes 1 to 5	544 (14.9)	113 (20.8)	1.3 (1.0-1.7)
More than class 5	1928 (52.9)	304 (15.8)	1.00
**Wealth index quartile**‡			
I	777 (21.3)	179 (23.0)	1.8 (1.3-2.3)
II	861 (23.6)	179 (20.8)	1.6 (1.2-2.1)
III	945 (25.9)	167 (17.7)	1.3 (1.0-1.7)
IV	1063 (29.2)	145 (13.6)	1.00
**Sex**			
Boy	1939 (53.2)	311 (16.0)	1.00
Girl	1708 (46.8)	359 (21.0)	1.4 (1.2-1.6)
**Gestation period**#			
6-7 months	33 (0.9)	29 (87.9)	34.0 (11.6-99.6)
8 months	701 (19.2)	178 (25.4)	1.8 (1.5-2.3)
>8 months	2910 (79.9)	460 (15.8)	1.00
**Birth order**#			
1st	1110 (30.5)	243 (21.9)	1.00
2nd	1019 (28.0)	169 (16.6)	0.8 (0.6-1.0)
>2nd	1515 (41.6)	255 (16.8)	0.8 (0.6-1.0)
**Place of delivery**#§			
Public sector facility	2622 (72.0)	475 (18.1)	1.00
Private sector facility	890 (24.4)	163 (18.3)	1.0 (0.8-1.3)
Home/on route	132 (3.6)	29 (22.0)	1.2 (0.8-1.8)
**Current status of livebirth**			
Died on day 0 of birth	26 (0.7)	10 (38.5)	1.9 (0.8-4.5)
Died between day 1-27 of birth	40 (1.1)	18 (45.0)	1.8 (0.9-3.8)
Died between day 28 and 11 months of age	22 (0.6)	9 (40.9)	2.6 (1.1-6.4)
Alive	3559 (97.6)	633 (17.8)	1.00

*****Data not available for 12 livebirths
†p-value <0.001, chi-square test of significance
§p-value= 0.536, chi-square test of significance
#Data not available for 3 livebirths
‡Data not available for 1 livebirth
