# Supplementary material for: Implications of the availability and distribution of birth weight on addressing neonatal mortality: population-based assessment from Bihar state of India
Source: BMJ Open. 2022 Jun 21;12(6):e061934. doi: 10.1136/bmjopen-2022-061934 (PMC9214371; doi:10.1136/bmjopen-2022-061934)
Supplement: Author's manuscript [file bmjopen-2022-061934.draft_revisions.pdf]

BMJ Open

BMJ Open is committed to open peer review. As part of this commitment we make the peer review history of every article we publish publicly available.

When an article is published we post the peer reviewers' comments and the authors' responses online. We also post the versions of the paper that were used during peer review. These are the versions that the peer review comments apply to.

The versions of the paper that follow are the versions that were submitted during the peer review process. They are not the versions of record or the final published versions. They should not be cited or distributed as the published version of this manuscript.

BMJ Open is an open access journal and the full, final, typeset and author-corrected version of record of the manuscript is available on our site with no access controls, subscription charges or pay-per-view fees (http://bmjopen.bmj.com).

If you have any questions on BMJ Open's open peer review process please email info.bmjopen@bmj.com

BMJ Open

Implications of the availability and distribution of birthweight on addressing neonatal mortality:findings from Bihar state of India

Journal:	*BMJ Open*
Manuscript ID	bmjopen-2022-061934
Article Type:	Original research
Date Submitted by the Author:	11-Feb-2022
Complete List of Authors:	KUMAR, ANIL; Public Health Foundation of India,<br>George, Sibin; Public Health Foundation of India<br>Akbar, Md.; Public Health Foundation of India<br>Bhattacharya, Debarshi; Bill & Melinda Gates Foundation India<br>Nanda, Priya; Bill and Melinda Gates Foundation India<br>Dandona, Lalit; Public Health Foundation of India<br>Dandona, Rakhi; Public Health Foundation of India; University of Washington
Keywords:	PUBLIC HEALTH, Community child health < PAEDIATRICS, EPIDEMIOLOGY

SCHOLARONE™
Manuscripts

I, the Submitting Author has the right to grant and does grant on behalf of all authors of the Work (as defined in the below author licence), an exclusive licence and/or a non-exclusive licence for contributions from authors who are: i) UK Crown employees; ii) where BMJ has agreed a CC-BY licence shall apply, and/or iii) in accordance with the terms applicable for US Federal Government officers or employees acting as part of their official duties; on a worldwide, perpetual, irrevocable, royalty-free basis to BMJ Publishing Group Ltd ("BMJ") its licensees and where the relevant Journal is co-owned by BMJ to the co-owners of the Journal, to publish the Work in this journal and any other BMJ products and to exploit all rights, as set out in our licence.

The Submitting Author accepts and understands that any supply made under these terms is made by BMJ to the Submitting Author unless you are acting as an employee on behalf of your employer or a postgraduate student of an affiliated institution which is paying any applicable article publishing charge ("APC") for Open Access articles. Where the Submitting Author wishes to make the Work available on an Open Access basis (and intends to pay the relevant APC), the terms of reuse of such Open Access shall be governed by a Creative Commons licence – details of these licences and which Creative Commons licence will apply to this Work are set out in our licence referred to above.

Other than as permitted in any relevant BMJ Author's Self Archiving Policies, I confirm this Work has not been accepted for publication elsewhere, is not being considered for publication elsewhere and does not duplicate material already published. I confirm all authors consent to publication of this Work and authorise the granting of this licence.

For peer review only

Implications of the availability and distribution of birthweight on addressing neonatal mortality: findings from Bihar state of India

G. Anil Kumar[1], Sibin George[1], Md. Akbar[1], Debarshi Bhattacharya[3], Priya Nanda[3], Lalit Dandona[1,2], Rakhi Dandona[1,2]

[1]Public Health Foundation of India, Gurugram, National Capital Region, India

[2]Institute for Health Metrics and Evaluation, University of Washington, Seattle, USA

[3]Bill & Melinda Gates Foundation, India Country Office, New Delhi, India

Corresponding author: Prof. Rakhi Dandona, Public Health Foundation of India, Gurugram, Haryana – 122002, India; rakhi.dandona@phfi.org

Short title: Birthweight in Bihar

For peer review only

ABSTRACT

Objective: A large proportion of neonatal deaths in India are attributable to low birthweight (LBW). We report population-based distribution and determinants of birthweight in Bihar state, and on the perceptions about birthweight among carers.

Design: A cross-sectional household survey in a state representative sample of 6,007 livebirths born in 2018-2019. Mothers provided detailed interviews on sociodemographic characteristics and birthweight, and their perceptions on LBW (birthweight <2500 g). We report on birthweight availability, LBW prevalence, neonatal mortality rate (NMR) by birthweight, and perceptions of mothers on LBW implications.

Setting: Bihar state, India

Participants: Women with livebirth between October 2018 to September 2019

Results: A total of 5,021 (83.5%) livebirths participated, and 3,939 (78.4%) were weighed at birth. LBW prevalence among those with available birthweight was 18.4% (95% CI 17.1-19.7). Majority (87.5%) of the livebirths born at home were not weighed at birth. LBW prevalence decreased and birthweight >=2,500 g increased significantly with increasing wealth index quartile. NMR was significantly higher in livebirths weighing <1,500 g (11.3%; 95% CI 5.1-23.1) and 1,500-1,999 g (8.0%; 95% CI 4.6-13.6) than those weighing >=2,500 g (1.3%, 95% CI 0.9-1.7. Assuming proportional correspondence of LBW and NMR in livebirths with and without birthweight, the estimated LBW among those without birthweight was 35.5% (95% CI 33.0-38.0) and among all livebirths irrespective of birthweight availability was 23.0% (95% CI 21.9-24.2). Seventy percent of mothers considered LBW to be a sign of sickness, 59.5% perceived it as a risk of developing other illnesses, and 8.6% as having an increased probability of death.

Conclusions: Missing birthweight is substantially compromising the planning of interventions to address LBW at the population-level. Variations of LBW by place of delivery and socio-demographic indicators, and the perceptions of carers about LBW can facilitate appropriate actions to address LBW and the associated neonatal mortality.

For peer review only - http://bmjopen.bmj.com/site/about/guidelines.xhtml

STRENGTHS AND LIMITATIONS OF THE STUDY

- Data on birthweight documented for a representative sample of livebirths including neonatal deaths
- Documentation of birthweight based on recall, which are of reasonable quality based on the global criterion
- Perceptions of care-givers on low birthweight documented in the same population

For peer review only

For peer review only - http://bmjopen.bmj.com/site/about/guidelines.xhtml

INTRODUCTION

Global nutrition targets include a 30% reduction in low birthweight (LBW, weight less than 2500 g) prevalence between 2012 and 2030.(1) LBW is a significant indicator of not only maternal and fetal health predicting mortality and stunting, but also of adult-onset chronic conditions.(2-7) The global LBW prevalence was estimated at 14·6% in 2015,(8, 9) and short gestation for birthweight accounted for an estimated 1·43 million deaths and 139 million DALYs in 2017.(10)

South Asia, with India as its largest component, was estimated to have the highest LBW prevalence for any region in the world in 2015 as per the most recent global update on LBW prevalence which provided country-level estimates.(8, 9) However, LBW prevalence for India was not estimated in that report due to quality concerns with the available data.(8) We have reported LBW prevalence of 21·4% in India in 2017 as part of the Global Burden of Disease (GBD) Study,(11) and that 83% of neonatal deaths could be attributed to LBW in India in 2017.(12) LBW prevalence has shown modest decline over time in India, and it is projected that India is unlikely to meet the LBW national and global nutrition targets.(11) The inadequate availability and quality of birthweight data in India, like many low-income and middle-income countries, is a major hindrance in tracking LBW as a priority.(8, 9, 11)

In this background, we report on a population-based assessment of birthweight in the Indian state of Bihar, which is among the most populous Indian states accounting for a significant burden of neonatal mortality.(12) The LBW prevalence in Bihar was estimated as 23.4% in 2017 in the GBD study.(11) The aim of this report is to provide nuanced data for policy makers and program planners on the availability and distribution of birthweight, and implications of birthweight non-availability on robustness of LBW estimate which is of utmost significance in planning of interventions to reduce LBW in order to address neonatal mortality. Furthermore, we present the perceptions about LBW among the carers which can improve specificity of interventions to address LBW. We use data as is without smoothening or imputation in order to highlight for the policy makers the gaps in the birthweight data that are to be addressed for meaningful action.(8, 11)

For peer review only - http://bmjopen.bmj.com/site/about/guidelines.xhtml

METHODS

The ethics approval for this study was provided by the Institutional Ethics Committee of Public Health Foundation of India (Study number TRC-IEC 418/19). Written informed consent were obtained from all respondents who could read and write, and the information sheet and consent form were explained by the interviewer to those who could not read/write and their thumb impressions were obtained.

For the survey, a state representative sample of 6,000 livebirths was selected using a multistage sampling approach from 37 of the 38 districts of Bihar state, excluding the Lakhisarai district. In the first stage, 70 functioning community/primary health centres (CHC/PHC) were randomly sampled with probability proportional to population size from a total of 445 functioning CHC/PHCs, with each catering to an average of 84 villages. In the next stage, five villages were selected from the catchment area of each of the selected CHC/PHC using the village list available in the Census 2011.(13) To arrive at a cluster size of 300 households, villages with <300 households were combined with an adjacent village, and the large villages were split into equal-sized segments of 300 households using natural boundaries. In total, 350 clusters were sampled using a systematic sampling. Each selected cluster was mapped and all the households (a household was defined as people eating from the same kitchen) were enumerated to identify the livebirths delivered by women aged 15-49 years between October 2018 to September 2019.

The mother/care-giver of each identified livebirth was contacted for a detailed interview irrespective of whether the baby was currently alive. Details on the socio-demography, the pregnancy, delivery, and postnatal care of the eligible livebirth were documented. Specifically, for the analysis reported in this paper, birthweight was recorded from the mother or caregiver of the child based on their recall. We also documented the mother/caretaker's perception of the birthweight for each livebirth (very weak, weak, normal, overweight), and whether the mother/caretaker perceived low birthweight in a baby to be an indication of sickness, and if so why. Furthermore, the possible reasons for LBW in babies, how to prevent LBW, and if the mother/care-

For peer review only - http://bmjopen.bmj.com/site/about/guidelines.xhtml

taker thought if the delivery process was different based on the birthweight were also documented. The questionnaire was developed in English and then translated into Hindi (local language), after which it was back-translated into English to ensure the accurate and relevant meaning and intent of the questions. Pilot testing of the questionnaires was carried out and modifications made as necessary. Data were collected between November 2019 and January 2020 using Open Development Kit by interviewers trained in study procedures. Data entered were scrutinized using the internal consistency checks built in to detect and correct errors using standardised procedures to meet the data quality. To further improve data quality, spot checks were conducted by the supervisors in 10% of the households and back checks were done in 15% of the households. At least three attempts were made to reach out to all the eligible livebirths.

We tested the quality of birthweight data by using the criteria utilised for the report on the global LBW prevalence estimates.(8) Poor quality data was defined as extreme heaping with >55% of all birthweights falling on three values (2500 g, 3000 g, or 3500 g); >10% of births weighed at least 4500 g; or excessive heaping on the tail end of the birthweight distribution with more than 5% of birthweights at 250–500 g and 5500 g.(8) We report on the quality of birthweight data, and for which livebirths the values of 2500 g, 3000 g, or 3500 g are more likely to be reported at the population-level.

We categorised birthweight into five categories for analysis - <1,500 g, 1,500-1,999 g, 2,000-2,499 g, <2,500 g (LBW), and 2,500 g or more. We present birthweight prevalence per 100 livebirths for these five categories with 95% confidence intervals, and also for not being weighted at birth, and for birthweight could not be recalled considering all livebirths irrespective of birthweight availability. We then report birthweight prevalence for these five birthweight categories considering only the livebirths for whom birthweight was available. Among these, the prevalence and mean birthweight with standard deviation (SD) is also reported by maternal age, maternal education, wealth index, sex of the baby, length of the pregnancy, place of delivery, and based on livebirth survival. Wealth index was estimated using the standard questions and methods used in the National Family Health

For peer review only

For peer review only - http://bmjopen.bmj.com/site/about/guidelines.xhtml

Survey.(14) Two separate multiple logistic regressions were run to investigate the association of not being weighted at birth among all livebirths, and for LBW among the livebirths with birthweight available with the above variables with all the variables introduced simultaneously in the model. Odds ratio with 95% CI are presented for the regression analysis.

We explored the association of neonatal and post-neonatal mortality with birthweight. Based on the difference in neonatal mortality rates between livebirths for whom birthweight was available versus those for whom birthweight was not available, we also report proportionately adjusted LBW prevalence in those with birthweight available to estimate the LBW prevalence in those with birthweight not available. In addition, a variety of perceptions of the caregivers about LBW are reported. All analysis was performed using STATA 13.1 software (Stata Corp, USA).

Patient and Public Involvement

Patients or the public were not involved in the design, or conduct, or reporting, or dissemination plans of our research.

For peer review only - http://bmjopen.bmj.com/site/about/guidelines.xhtml

RESULTS

We identified 6,007 livebirths representative of the Bihar state between October 2018 to September 2019 from 5,852 women aged 15-49 years in 55,475 households. Detailed interview was available for 5,021 (83.6%) livebirths, majority (98.2%) of whom were singleton births, 2,614 (52.1%) were boys, 2,870 (57.2 %) were born in a public health facility, and 150 (3%) were currently not alive. Of the 5,021 livebirths, 3,939 (78.4%) were weighed at birth but birthweight could not be recalled for 292 (7.4%, 95% CI 6.6-8.3) livebirths.

Quality of birthweight data

Considering the 3,647 livebirths with birthweight available, 52% of all birthweight values fell on 2,500 g, 3,000 g, or 3,500 g; 1.6% livebirths weighed at least 4,500 g; and 0.36% of birthweights were either at 250–500 g or 5,500 g. This indicates data to be of reasonable quality, as the heaping was less than the criteria for poor quality data.(8) Significant variation was seen in the reporting of birthweight values of 2,500 g, 3,000 g, and 3,500 g by maternal age (chi-square, p=0.008), maternal education (chi-square, p<0.001), and place of delivery (chi-square, p=0.028) as shown in Supplementary Figure 1.

Distribution of birthweight among all livebirths

Considering all livebirths irrespective of birthweight availability, prevalence of birthweight >=2500 g was 59.3% (95% CI 57.9-60.6), of LBW was 13.3% (95% CI 12.4-14.3), and of livebirths not weighed at birth was 21.5% (95% CI 20.4-22.7) as shown in Supplementary Table 1. Using multiple logistic regression (Supplementary Table 2), the odds of not being weighed at birth were the highest for home births (OR 532.2; 95% CI 365.9-774.2) followed by for livebirths who had died on day 0 as compared with those who were currently alive (OR 8.6; 95% CI 3.6-20.5). Mothers who were not educated also had significantly higher odds of having the livebirth not weighed at birth (OR 1.8; 95% CI 1.3-2.5) as compared to mothers who had more than primary schooling (Supplementary Table 2).

Distribution of birthweight among livebirths with birthweight available

For peer review only - http://bmjopen.bmj.com/site/about/guidelines.xhtml

Among livebirths with birthweight available, the mean birthweight was 2,848.2 g with SD of ± 647.2 g (Table 1), and was significantly lower for livebirths born at 6-7 months of gestation (1,710.6 ± 577.4 g) and for livebirths of younger mothers aged <20 years (2,718.0 ± 642.5 g). Girls, livebirths belonging to lower wealth index quartile, and livebirths who did not survive were significantly more likely to have a lower mean birthweight as compared with boys, those belonging to higher wealth index quartile, and those currently alive, respectively (Table 1).

Table 1. Mean birthweight for livebirths between October 2018 to September 2019 for whom birthweight could be recalled in the Indian state of Bihar.

	Total	Availability of birth weight (% of total)	Mean birthweight (g)
**Overall**	**5,021**	**3,647 (72.6)**	**2,848.2 ± 647.2**
**Maternal age *†**			
15-19 years	529	407 (76.9)	2,718.0 ± 642.5
20-24 years	2,392	1,808 (75.6)	2,836.6 ± 646.3
25-29 years	1,453	1,028 (70.8)	2,911.8 ± 632.8
>=30 years	633	392 (61.9)	2,878.7 ± 662.5
**Maternal education§†**			
No education	1,907	1,172 (61.5)	2,801.0 ± 685.6
Classes 1 to 5	760	544 (71.6)	2,826.0 ± 664.4
More than class 5	2,350	1,928 (82.0)	2,885.4 ± 613.3
**Wealth index quartile#†**			
I	1,255	777 (61.9)	2,781.9 ± 690.1
II	1,255	861 (68.6)	2,800.7 ± 656.0
III	1,255	945 (75.3)	2,879.9 ± 659.2
IV	1,255	1,063 (84.7)	2,907.0 ± 588.0
**Sex‡**			
Boy	2,614	1,939 (74.2)	2,888.7 ± 647.1
Girl	2,407	1,708 (71.0)	2,802.3 ± 644.3
**Gestation period†**			
6-7 months	46	33 (71.7)	1,710.6 ± 577.4
8 months	944	701 (74.3)	2,735.7 ± 631.7
>8 months	4,027	2,910 (72.3)	2,889.7 ± 635.2
**Birth order†**			
1st	1,366	1,110 (81.3)	2,775.2 ± 628.5
2nd	1,369	1,019 (74.4)	2,892.5 ± 653.1
>2nd	2,282	1,515 (66.4)	2,874.8 ± 649.8
**Place of delivery§†**			
Public sector facility	2,870	2,622 (91.4)	2,839.3 ± 625.9
Private sector facility	1,022	890 (87.1)	2,880.7 ± 697.0
Home	1,125	132 (11.7)	2,839.2 ± 679.6
**Current status of livebirth‡**			
Died on day 0 of birth	57	26 (45.6)	2,644.2 ± 1,082.1

For peer review only - http://bmjopen.bmj.com/site/about/guidelines.xhtml

Died between day 1-27 of birth	58	40 (69.0)	2,611.3 ± 1,071.3
Died between day 28 and 11 months of age	35	22 (62.9)	2,368.2 ± 771.9
Alive	4,871	3559 (73.1)	2,855.3 ± 634.4

*Data not available for 14 livebirths
†Chi-square test of significance, p-value <0.001
§Data not available for 4 livebirths
#Data not available for 1 livebirth
‡Chi-square test of significance, p-value =0.001

The prevalence of LBW was 18.4 (95% CI 17.1-19.7), and that of birthweight <1,500 g was 1.5 (95% CI 1.1-1.9), of 1,500-1,999 g was 4.1 (95% CI 3.5-4.8), and of 2,000-2,400 g was 12.8 (95% CI 11.8-13.9) as shown in Table 2. LBW prevalence was 5.6 times higher among the babies who were born with 6-7 months of gestation as compared with those born >8 months of gestation (Table 2 and Figure 1). LBW prevalence decreased and that for birthweight >=2,500 g increased significantly (p<0.001) with increasing wealth index quartile (Table 2 and Figure 1). Using multiple logistic regression (Supplementary Table 2), the most significant odds of having LBW were for livebirths with gestation period of 6-7 months (OR 34.0; 95% CI 11.6-99.6).

Table 2. Prevalence of birthweight by categories among the livebirths who had birthweight available for select characteristics in the Indian state of Bihar for livebirths between October 2018 to September 2019.

	**Prevalence per 100 livebirths (95% confidence interval)**				
	**Birthweight >=2,500 g**	**Birthweight <2,500 g**	***Birthweight 2,000 - 2,499 g***	***Birthweight 1,500 - 1,999 g***	***Birthweight <1,500 g***
Overall	81.6 (80.3-82.9)	18.4 (17.1-19.7)	*12.8 (11.8-13.9)*	*4.1 (3.5-4.8)*	*1.5 (1.1-1.9)*
**Maternal age***					
15-19 years	73.0 (68.4-77.1)	27.0 (22.9-31.6)	*19.9 (16.3-24.1)*	*6.1 (4.2-8.9)*	*1.0 (0.4-2.6)*
20-24 years	81.5 (79.6-83.2)	18.5 (16.8-20.4)	*13.0 (11.5-14.6)*	*4.0 (3.2-5.1)*	*1.5 (1.0-2.2)*
25-29 years	86.0 (83.7-88.0)	14.0 (12.0-16.3)	*9.2 (7.6-11.2)*	*3.3 (2.4-4.6)*	*1.5 (0.9-2.4)*
>=30 years	80.6 (76.4-84.2)	19.4 (15.8-23.6)	*13.8 (10.7-17.6)*	*4.3 (2.7-6.9)*	*1.3 (0.5-3.0)*
**Maternal education§**					
No education	78.7 (76.2-80.9)	21.3 (19.1-23.8)	*14.0 (12.1-16.1)*	*5.1 (4.0-6.5)*	*2.2 (1.5-3.2)*
Class 1 to 5	79.2 (75.6-82.4)	20.8 (17.6-24.4)	*14.5 (11.8-17.7)*	*5.2 (3.6-7.4)*	*1.1 (0.5-2.4)*
More than class 5	84.2 (82.5-85.8)	15.8 (14.2-17.5)	*11.6 (10.3-13.1)*	*3.2 (2.5-4.1)*	*1.0 (0.6-1.5)*
**Wealth index quartile #**					
I	78.7 (76.2-80.9)	23.0 (20.2-26.1)	*15.3 (13.0-18.0)*	*5.8 (4.4-7.7)*	*1.9 (1.2-3.2)*
II	79.2 (75.6-82.4)	20.8 (18.2-23.6)	*14.1 (11.9-16.5)*	*4.9 (3.6-6.5)*	*1.9 (1.1-3.0)*
III	84.2 (82.5-85.8)	17.7 (15.4-20.2)	*13.2 (11.2-15.5)*	*3.2 (2.2-4.5)*	*1.3 (0.7-2.2)*

For peer review only - http://bmjopen.bmj.com/site/about/guidelines.xhtml

	Prevalence per 100 livebirths (95% confidence interval)				
	**Birthweight >=2,500 g**	**Birthweight <2,500 g**	***Birthweight 2,000 - 2,499 g***	***Birthweight 1,500 - 1,999 g***	***Birthweight <1,500 g***
IV	78.7 (76.2-80.9)	13.6 (11.7-15.8)	*9.6 (8.0-11.5)*	*3.1 (2.2-4.3)*	*0.9 (0.5-1.7)*
**Sex**					
Boy	84.0 (82.3-85.5)	16.0 (14.5-17.7)	*10.8 (9.5-12.2)*	*4.0 (3.2-4.9)*	*1.3 (0.9-1.9)*
Girl	79.0 (77.0-80.9)	21.0 (19.2-23.0)	*15.1 (13.5-16.9)*	*4.3 (3.4-5.3)*	*1.6 (1.1-2.4)*
**Gestation period§**					
6-7 months	12.1 (4.6-28.5)	87.9 (71.5-95.5)	*24.2 (12.5-41.8)*	*36.4 (21.8-54.0)*	*27.3 (14.7-45.0)*
8 months	74.6 (71.3-77.7)	25.4 (22.3-28.8)	*17.7 (15.0-20.7)*	*5.9 (4.3-7.9)*	*1.9 (1.1-3.2)*
>8 months	84.2 (82.8-85.5)	15.8 (14.5-17.2)	*11.5 (10.4-12.7)*	*3.3 (2.7-4.0)*	*1.0 (0.7-1.4)*
**Birth order**					
1st	78.1 (75.6-80.5)	21.9 (19.6-24.4)	*15.4 (13.4-17.7)*	*5.2 (4.1-6.7)*	*1.3 (0.8-2.1)*
2nd	83.4 (81.0-85.6)	16.6 (14.4-19.0)	*12.0 (10.1-14.1)*	*3.2 (2.3-4.5)*	*1.4 (0.8-2.3)*
>2nd	83.2 (81.2-85.0)	16.8 (15.0-18.8)	*11.5 (10.0-13.2)*	*3.8 (3.0-4.9)*	*1.5 (1.0-2.3)*
**Place of delivery§**					
Public sector facility	81.9 (80.4-83.3)	18.1 (16.7-19.6)	*13.2 (12.0-14.6)*	*3.6 (2.9-4.4)*	*1.3 (0.9-1.8)*
Private sector facility	81.7 (79.0-84.1)	18.3 (15.9-21.0)	*11.4 (9.4-13.6)*	*5.4 (4.1-7.1)*	*1.6 (0.9-2.6)*
Home	78.0 (70.1-84.3)	22.0 (15.7-29.9)	*14.4 (9.4-21.5)*	*5.3 (2.5-10.7)*	*2.3 (0.7-6.8)*
**Current status of livebirth**					
Died on day 0 of birth	61.5 (41.7-78.2)	38.5 (21.8-58.3)	*11.5 (3.7-30.8)*	*19.2 (8.1-39.2)*	*7.7 (1.9-26.6)*
Died between day 1-27 of birth	55.0 (39.4-69.7)	45.0 (30.3-60.6)	*17.5 (8.5-32.6)*	*17.5 (8.5-32.6)*	*10.0 (3.8-24.0)*
Died between day 28 and 11 months of age	59.1 (37.7-77.5)	40.9 (22.5-62.3)	*13.6 (4.3-35.5)*	*18.2 (6.8-40.3)*	*9.1 (2.2-30.7)*
Alive	82.2 (80.9-83.4)	17.8 (16.6-19.1)	*12.8 (11.7-13.9)*	*3.8 (3.2-4.4)*	*1.3 (1.0-1.7)*

Of the 670 LBW babies, the parents of 463 (69.1%) livebirths were informed by the health provider that the baby was weak/LBW. This proportion was 87.2% for the 203 livebirths with birthweight of <2,000 g and 94.3% for 53 livebirths with birthweight of <1,500 g. Considering the 190 facility livebirths with birthweight <2,000 g, livebirths at public facility (84%) were significantly less likely to be informed by the health provider of the baby being weak/having LBW as compared with those born in a private sector facility (93.6%; Z test for significance p<0.1).

Mortality and birthweight

A total of 150 (3.0%) livebirths were not currently alive) of whom 114 (76%) had died during the neonatal period (Table 1). The neonatal mortality rate in livebirths weighing <1,500 g (11.3%; 95% CI 5.1-23.1) and 1,500-1,999 g (8.0%; 95% CI 4.6-13.6) was significantly higher than in those weighing

For peer review only - http://bmjopen.bmj.com/site/about/guidelines.xhtml

>=2,500 g (Table 3). The neonatal mortality rate in livebirths for whom birthweight was not available (3.5; 95% CI 2.6-4.6) was almost twice as high as compared with those for whom birthweight was available (1.8%, 95% CI1.4-2.3) as shown in Table 3. Based on this 93% higher neonatal mortality rate among livebirths for whom birthweight was not available, and assuming a direct correspondence between neonatal mortality rate and LBW, we estimated that LBW among livebirths for whom birthweight was not available would be 35.5% (95% CI 33.0-38.0), that is, 93% higher than the 18.4% LBW among livebirths for whom birthweight was available. Based on the proportions of these two groups among all livebirths, we estimated an overall LBW of 23.0% (95% CI 21.9-24.2) among all livebirths.

Table 3. Mortality by birthweight categories among the livebirths born between October 2018 to September 2019 in the Indian state of Bihar. CI denotes confidence interval.

Birthweight	Number of livebirths	Number of neonatal deaths	Neonatal mortality rate, % (95% CI)	Number of deaths in post neonatal period to 11 months of age	Post-neonatal mortality rate to 11 months of age, % (95% CI)
>=2,500 g	2,977	38	1.3 (0.9-1.7)	13	0.4 (0.3-0.8)
<2,500 g	670	28	4.2 (2.9-6.0)	9	1.3 (0.7-2.6)
<1,500 g	*53*	*6*	*11.3 (5.1-23.1)*	*2*	*3.8 (0.9-14.0)*
1,500-1,999 g	*150*	*12*	*8.0 (4.6-13.6)*	*4*	*2.7 (1.0-6.9)*
2,000-2,499 g	*467*	*10*	*2.1 (1.2-3.9)*	*3*	*0.6 (0.2-2.0)*
**Birthweight available**	3,647	66	1.8 (1.4-2.3)	22	0.6 (0.4-0.9)
Not recalled	292	15	5.1 (3.1-8.4)	0	0
Not weighed at birth	1,082	33	3.0 (2.2-4.3)	14	1.3 (0.8-2.2)
**Birthweight not available**	1,374	48	3.5 (2.6-4.6)	14	1.0 (0.6-1.7)
**All livebirths**	**5,021**	**114**	**2.3 (1.9-2.7)**	**96**	**0.7 (0.5-1.0)**

Respondent's perceptions about LBW

Mothers were the predominant respondent in the survey (99.8%). Figure 2 shows the perception of mothers on the birthweight of their livebirth. Overall, 74.7% (3,748) of all mothers of livebirth, 88.1% (2,622) of mothers of livebirths >= 2,500g, and 25.5% (170) of mothers of LBW livebirths perceived their newborns to be of normal weight. Perception of weak or very weak was higher in LBW livebirths (73.3%) as compared with >= 2,500g livebirths (11%). Among the 53 livebirths with birthweight <1,500 g, 36 (67.9%) were perceived to be very weak, 9 (17%) weak and 6 (1.3%) of normal weight by the mother.

For peer review only - http://bmjopen.bmj.com/site/about/guidelines.xhtml

For peer review only

These perceptions are not mutually exclusive.

A total of 3,527 (70.2%) mothers considered LBW to be a sign of sickness/illness. Among these 3,527 women, 2,988 (84.2%) perceived it as a risk of developing other illnesses, 1,764 (50%) considered it a risk for weak growth, and 433 (12.3%) perceived it as having an increased probability of death (not mutually exclusive). Among the 1,350 (26.9%) women who did not consider LBW to be a sickness in a newborn, 1,308 (96.9%) felt that the baby would gain weight after birth and hence there was nothing to worry. Majority (4,570; 91%) of the mothers thought that LBW baby needed extra care; and the extra care practices commonly reported (not mutually exclusive) were oil massage (76.4%), exclusive breastfeeding (74.3%), seeking health care advice (46.6%), and keeping the baby warm (31.2%).

Figure 2 shows the possible reasons of LBW as reported by the mothers (not mutually exclusive). Mother eating less during pregnancy (74.7%), inadequate diet during pregnancy (43.8%), and weak mother (33.2%) were the most cited reasons for LBW baby. Majority of the mothers (94.9%) reported that intake of nutritious diet during pre- and during pregnancy can prevent LBW, followed by full antenatal care check-up (28.3%) and iron and folic acid intake (23.3%). A total of 3,026 (60.8%) mothers perceived the delivery process to be different depending on the birthweight of baby; 2,515 (83.1%) felt that delivery of LBW baby was easier than that of a normal weight baby, 891 (29.4%) thought that C-section was needed less for LBW babies, and 874 (28.9 %) felt that duration of labour was shorter for them (not mutually exclusive).

For peer review only

DISCUSSION

We present the estimates for birthweight prevalence across various categories in the Indian state of Bihar, including LBW prevalence which is essential for tracking progress towards the national and global nutrition targets. These estimates are presented in two ways – including and excluding livebirths based on birthweight availability – to highlight the need for improved birthweight availability to arrive at robust understanding of LBW prevalence for appropriate action both within the health system and the community. Socio-demographic distribution of livebirths for whom birthweight was not available can facilitate formulating specific actions in these populations to improve birthweight availability. Notably, the perceptions of mothers regarding reasons for LBW and its implications can provide a framework for developing relevant actions to improve care of LBW babies and possible actions to reduce LBW prevalence.

Birthweight was missing for 1 out of 4 livebirths in this population. Extrapolating our findings to the estimated 2.5 million livebirths in 2019 in Bihar, 543,000 livebirths were not weighted at birth and recall was not available for 146,600. Though home births accounted for only 22% of all livebirths in this population, these accounted for majority of the livebirths who were not weighted at birth. Therefore, until facility births can be increased further in the long-term that could result in increased birthweight measurement, tracking LBW as a priority target is not possible unless urgent targeted efforts are made in the short-term to engage with the health providers who assist with home births to improve birthweight availability.

Overall, birthweight data in our study was of reasonable quality as per the criteria used in the recent report on global estimation of LBW prevalence.(8) Unlike other reports,(8, 9) we did not smoothen the data for heaping, but have presented data as is to enhance understanding of where heaping was more likely to be reported to facilitate development of targeted approach in addressing this heaping. For the policy makers and program planners it is imperative to note where most action is needed to improve robustness of birthweight estimates. One of the assumptions made in the recent global report on LBW prevalence was that missing birthweights are missing at random and

For peer review only - http://bmjopen.bmj.com/site/about/guidelines.xhtml

that the true distribution of birthweights in a population can be approximated by a mixture of two normal distributions.(8) Our data has highlight that birthweight is not missing at random but in specific sub-groups, and this may be need to be taken into account in assumptions for global estimates.

The LBW prevalence estimated was 18.4% considering only livebirths with birthweight available, and 23% in all livebirths by proportionately adjusting for those who did not birthweight available based on their higher neonatal mortality rate. Even though the adjustment made for neonatal mortality is fairly simplistic, the extent of variation in LBW prevalence with this adjustment conveys the enormous implications of non-availability of birthweight for the planning of interventions and to appropriately allocate resources to address LBW at the population-level. Those without birthweight accounted for one-third of all neonatal deaths, and birthweight availability was less than half for the livebirths who had died on day 0. Importantly, the LBW prevalence was estimated to be almost twice among livebirths for whom birthweight was not available versus those for whom birthweight was available. This finding is of significance as we have previously reported that 50% of all neonatal mortality in the state to be in 0-2 days of birth, with 35% of them not weighted at birth.(15) Though the current study included only livebirths, our previous work in Bihar has also documented birthweight non-availability at 85% for stillbirths.(16) One of the proposed newborn quality of care indicator at health-facility level in low- and middle-income setting is facility neonatal mortality rate disaggregated by birth weight.(17) With majority of births now in the facilities, urgent and sustained effort is needed to track this quality indicator on a routine basis, which is currently not tracked in the Indian health information system. Interestingly, the Civil Registration System captures the birthweight for all births but that data is not available in public domain to comment on availability and quality of that data.(18) As LBW and short gestation are the predominant risk factors for neonatal mortality in India and in Bihar,(12) ensuring birthweight is measured for all livebirths irrespective of survival at birth is extremely important. Understanding the health providers perspectives on the need of birthweight measurement and quality is an

For peer review only - http://bmjopen.bmj.com/site/about/guidelines.xhtml

understudied issue,(19) and effort to improve this understanding is needed urgently to improve birthweight documentation.

A significant focus of neonatal health programs is on caring for the small and sick newborns, and communication with the carer/family is an integral part for their meaningful participation.(20) Seven in 10 carers of LBW babies were informed by the health provider that the baby was weak/LBW, and this proportion increased with decreasing birthweight. Some additional effort is needed in the public sector facilities as the families of babies born there were less likely to be informed than those in the private sector and informing birthweight and its implications by them to the family. Importantly, 70% of the mothers interviewed considered LBW to be a sign of sickness/illness, and such level of awareness could be translated not only into demand for availability of birthweight in the community, but also to increase uptake of relevant interventions for LBW babies.(21-26)

The finding of decrease in prevalence of LBW and increase in birthweight >=2,500 g with increasing wealth index quartile is not surprising, given that maternal undernutrition is associated with poor maternal-fetal outcomes including LBW.(2-6, 27) Despite decades of efforts in India to tackle malnutrition, it was the predominant risk factor for under-5 deaths in every state of India in 2017, accounting for 68·2% of the total under-5 deaths.(11) Globally, India has the highest prevalence of BMI lower than 16 in women, with less prevalence in women belonging to higher wealth index.(28) Evidence from Bangladesh suggests that low levels of women's empowerment are associated with maternal undernutrition as well as with delivering LBW babies, and empowerment is lower in women of lower wealth index.(27) What is noteworthy is that majority of the women in our study were well aware of the link between maternal nutrition and LBW, highlighting that facilitators are needed to translate this awareness into action to improve maternal nutrition, which can be achieved by bringing convergence of variety of nutrition-related activities of various government ministries and stakeholders for maternal health across the life cycle.(11, 29-33)

For peer review only - http://bmjopen.bmj.com/site/about/guidelines.xhtml

Documentation of birthweight based on recall in this study could be considered a limitation, however, these data were of reasonable quality using the global criterion.(8) The strengths of our study include an attempt to estimate LBW for all livebirths at the population level, and inclusion of carer perspectives in addition to birthweight availability that can facilitate actionable interventions or further implementation research to improve tracking of LBW, which is a priority global health indicator.

In conclusion, significant efforts are needed beyond what is has been done so far to increase the availability and quality of birthweight in order to improve robustness of LBW estimates, which can help planning of appropriate interventions and investments to address this important risk factor of neonatal mortality. Without robust birthweight estimates, India may not able to address neonatal mortality effectively to meet the Sustainable Development Goal by 2030.

For peer review only

For peer review only - http://bmjopen.bmj.com/site/about/guidelines.xhtml

ACKNOWLEDGEMENTS

The authors acknowledge the contributions of Moutushi Majumder and Kaavya Singh from Public Health Foundation of India, and Asif Iqbal and Vipul Singhal from the Oxford Policy Management, India for data collection and data management.

AUTHORS' CONTRIBUTIONS

RD and GAK had full access to data in the study, take full responsibility for the integrity of data and accuracy of the data analysis, and had final responsibility for the decision to submit for publication; RD, GAK and LD conceptualized the study; RD guided the data analysis and drafted the manuscript; SG performed data analysis; MA guided data collection; MA, DB, PN and LD contributed to data analysis and interpretation; all authors approved the final manuscript.

FUNDING

This work was supported by Bill & Melinda Gates Foundation grant number INV-007989.

COMPETING INTEREST

PN and DB are employees of Bill & Melinda Gates Foundation. Other authors declare no completing interests.

DATA AVAILABILITY STATEMENT

All the data of the current study is available with the corresponding author, can be made available on request.

ETHICS APPROVAL

The ethics approval for this study was provided by the Institutional Ethics Committee of Public Health Foundation of India (Study number TRC-IEC 418/19).

For peer review only

REFERENCES

1. World Health Organization. Global nutrition monitoring framework: operational guidance for tracking progress in meeting targets for 2025. Geneva: WHO; 2017.
2. Katz J, Lee AC, Kozuki N, et al. Mortality risk in preterm and small-for-gestational-age infants in low-income and middle-income countries: a pooled country analysis. *Lancet*. 2013;382(9890):417-25.
3. Loret de Mola C, de França GV, Quevedo Lde A, et al. Low birth weight, preterm birth and small for gestational age association with adult depression: systematic review and meta-analysis. *Br J Psychiatry*. 2014;205(5):340-7.
4. Oudgenoeg-Paz O, Mulder H, Jongmans MJ, et al. The link between motor and cognitive development in children born preterm and/or with low birth weight: A review of current evidence. *Neurosci Biobehav Rev*. 2017;80:382-93.
5. Shinzawa M, Tanaka S, Tokumasu H, et al. Association of Low Birth Weight With Childhood Proteinuria at Age 3 Years: A Population-Based Retrospective Cohort Study. *Am J Kidney Dis*. 2019;74(1):141-3.
6. Victora CG, Adair L, Fall C, et al. Maternal and child undernutrition: consequences for adult health and human capital. *Lancet*. 2008;371(9609):340-57.
7. Wojcik W, Lee W, Colman I, et al. Foetal origins of depression? A systematic review and meta-analysis of low birth weight and later depression. *Psychol Med*. 2013;43(1):1-12.
8. Blencowe H, Krasevec J, de Onis M, et al. National, regional, and worldwide estimates of low birthweight in 2015, with trends from 2000: a systematic analysis. *Lancet Glob Health*. 2019;7(7):e849-e60.
9. United Nations Children's Fund (UNICEF) and World Health Organization. UNICEF-WHO Low birthweight estimates: Levels and trends 2000–2015. Geneva: UNICEF and WHO; 2019.
10. GBD 2017 Risk Factor Collaborators. Global, regional, and national comparative risk assessment of 84 behavioural, environmental and occupational, and metabolic risks or clusters of risks for 195 countries and territories, 1990-2017: a systematic analysis for the Global Burden of Disease Study 2017. *Lancet*. 2018;392(10159):1923-94.
11. India State-Level Disease Burden Initiative Malnutrition Collaborators. The burden of child and maternal malnutrition and trends in its indicators in the states of India: the Global Burden of Disease Study 1990-2017. *Lancet Child Adolesc Health*. 2019;3(12):855-70.
12. India State-Level Disease Burden Initiative Child Mortality Collaborators. Subnational mapping of under-5 and neonatal mortality trends in India: the Global Burden of Disease Study 2000-17. *Lancet*. 2020;395(10237):1640-58.
13. Office of the Registrar General & Census Commissioner of India GoI, . C.D. Blockwise Primary Census Abstract Data (PCA) - Bihar: RGI; [Available from: http://censusindia.gov.in/pca/cdb_pca_census/Houselisting-housing-BR.html.
14. International Institute for Population Sciences. About NFHS: IIPS; [Available from: http://rchiips.org/nfhs/about.shtml.
15. Dandona R, Kumar GA, Bhattacharya D, et al. Distinct mortality patterns at 0-2 days versus the remaining neonatal period: results from population-based assessment in the Indian state of Bihar. *BMC Med*. 2019;17(1):140.
16. Dandona R, Kumar GA, Akbar M, et al. Deferred and referred deliveries contribute to stillbirths in the Indian state of Bihar: results from a population-based survey of all births. *BMC Med*. 2019;17(1):28.
17. Madaj B, Smith H, Mathai M, et al. Developing global indicators for quality of maternal and newborn care: a feasibility assessment. *Bull World Health Organ*. 2017;95(6):445-52I.
18. Office of the Registrar General & Census Commissioner of India GoI. Birth Report: RGI; [Available from: https://censusindia.gov.in/2011-Documents/CRS_Forms/CRS%20Forms.pdf.

For peer review only - http://bmjopen.bmj.com/site/about/guidelines.xhtml

19. Gladstone ME, Salim N, Ogillo K, et al. Birthweight measurement processes and perceived value: qualitative research in one EN-BIRTH study hospital in Tanzania. *BMC Pregnancy Childbirth*. 2021;21(Suppl 1):232.
20. World Health Organization. Standards for improving the quality of care for small and sick newborns in health facilities. Geneva: WHO; 2020.
21. Koenraads M, Phuka J, Maleta K, et al. Understanding the challenges to caring for low birthweight babies in rural southern Malawi: a qualitative study exploring caregiver and health worker perceptions and experiences. *BMJ Glob Health*. 2017;2(3):e000301.
22. Lydon M, Longwe M, Likomwa D, et al. Starting the conversation: community perspectives on preterm birth and kangaroo mother care in southern Malawi. *J Glob Health*. 2018;8(1):010703.
23. Nisha MK, Raynes-Greenow C, Rahman A, et al. Perceptions and practices related to birthweight in rural Bangladesh: Implications for neonatal health programs in low- and middle-income settings. *PLoS One*. 2019;14(12):e0221691.
24. Chan G, Bergelson I, Smith ER, et al. Barriers and enablers of kangaroo mother care implementation from a health systems perspective: a systematic review. *Health Policy Plan*. 2017;32(10):1466-75.
25. Mazumder S, Upadhyay RP, Hill Z, et al. Kangaroo mother care: using formative research to design an acceptable community intervention. *BMC Public Health*. 2018;18(1):307.
26. Smith ER, Bergelson I, Constantian S, et al. Barriers and enablers of health system adoption of kangaroo mother care: a systematic review of caregiver perspectives. *BMC Pediatr*. 2017;17(1):35.
27. Kabir A, Rashid MM, Hossain K, et al. Women's empowerment is associated with maternal nutrition and low birth weight: evidence from Bangladesh Demographic Health Survey. *BMC Womens Health*. 2020;20(1):93.
28. Razak F, Corsi DJ, Slutsky AS, et al. Prevalence of Body Mass Index Lower Than 16 Among Women in Low- and Middle-Income Countries. *JAMA*. 2015;314(20):2164-71.
29. Kim SS, Avula R, Ved R, et al. Understanding the role of intersectoral convergence in the delivery of essential maternal and child nutrition interventions in Odisha, India: a qualitative study. *BMC Public Health*. 2017;17(1):161.
30. McKerricher L, Petrucka P. Maternal nutritional supplement delivery in developing countries: a scoping review. *BMC Nutr*. 2019;5:8.
31. Noznesky EA, Ramakrishnan U, Martorell R. A situation analysis of public health interventions, barriers, and opportunities for improving maternal nutrition in Bihar, India. *Food Nutr Bull*. 2012;33(2 Suppl):S93-103.
32. Young MF, Bootwala A, Kachwaha S, et al. Understanding Implementation and Improving Nutrition Interventions: Barriers and Facilitators of Using Data Strategically to Inform the Implementation of Maternal Nutrition in Uttar Pradesh, India. *Curr Dev Nutr*. 2021;5(6):nzab081.
33. Ministry of Women and Child Development. Government of India. Administrative guidelines for implementation of National Nutrition Mission.: Ministry of Women and Child Development; 2018 [Available from: https://icds-wcd.nic.in/nnm/NNM-Web-Contents/UPPER-MENU/AdministrativeApproval-Guidelines/Administrative_Guidelines_NNM-26022018.pdf.

For peer review only - http://bmjopen.bmj.com/site/about/guidelines.xhtml

FIGURES

Figure 1. Distribution of birthweight by the gestation period and wealth index quartile for livebirths between October 2018 to September 2019 for whom birthweight was available in the Indian state of Bihar.

Figure 2. Factors perceived as responsible for low birthweight in babies among the mothers of livebirths between October 2018 to September 2019 in the Indian state of Bihar.

SUPPLEMENTARY MATERIAL

Supplementary Figure 1. Distribution of birthweight values of 2500 g, 3,000 g, and 3,500 g by select variables among the livebirths born between October 2018 to September 2019 for whom birthweight was available in the Indian state of Bihar.

Supplementary Table 1. Prevalence of birthweight by categories, of no recall, and of child not being weighted at birth for select characteristics in the Indian state of Bihar for livebirths between October 2018 to September 2019.

Supplementary Table 2. Association of low birthweight (LBW) among babies with birthweight available, and of not being weighted at birth among all livebirths with select variables using multiple logistic regression for livebirths between October 2018 to September 2019 in the Indian state of Bihar.

For peer review only

Figure 1. Distribution of birthweight by the gestation period and wealth index quartile for livebirths between October 2018 to September 2019 for whom birthweight was available in the Indian state of Bihar.

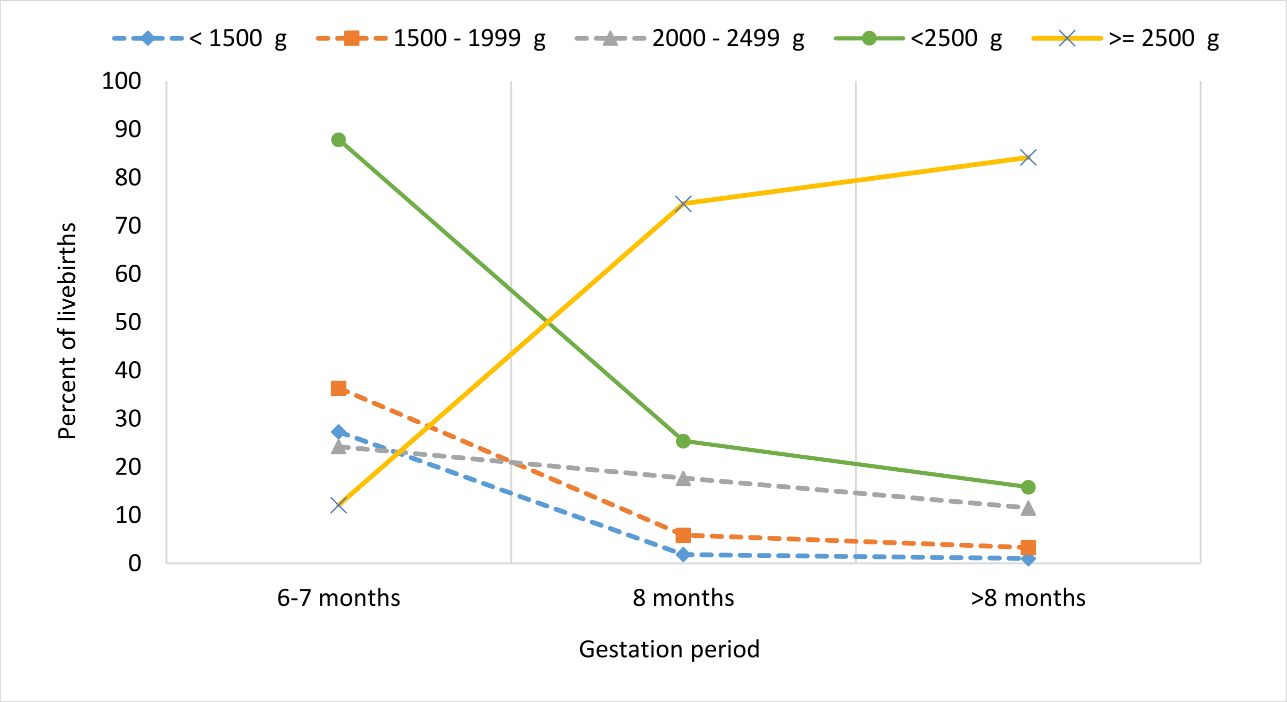

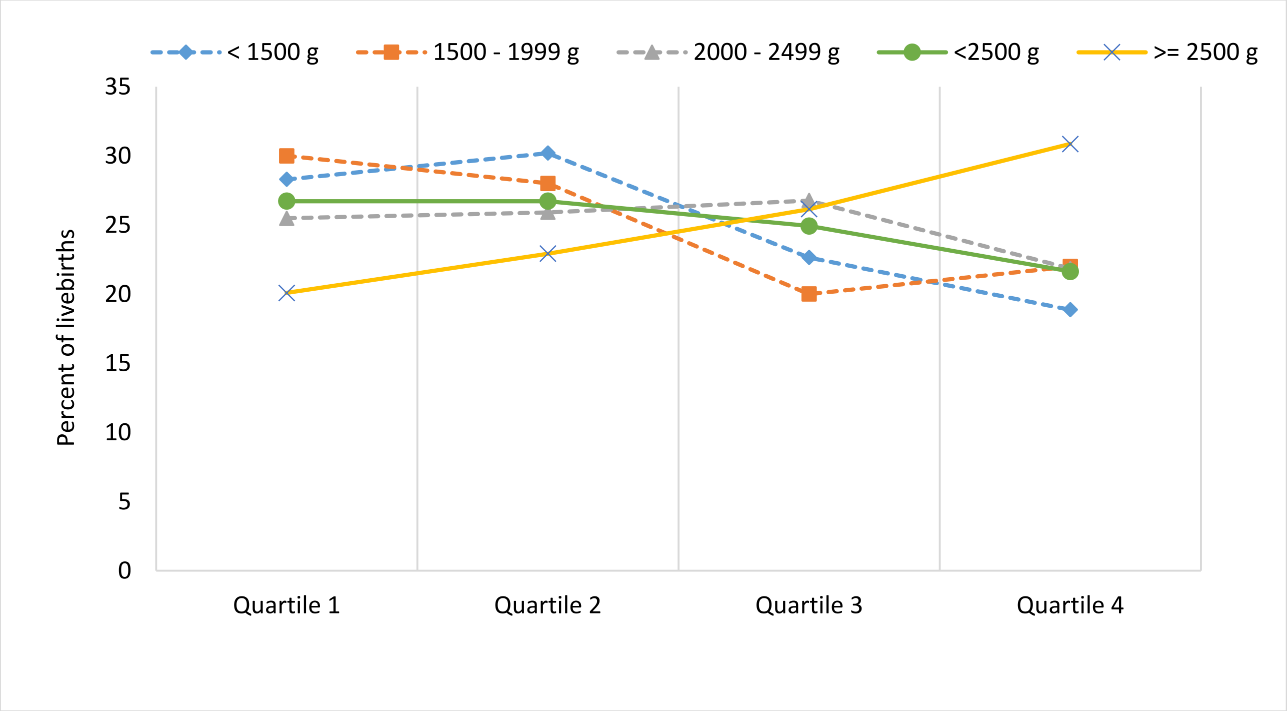

For peer review only - http://bmjopen.bmj.com/site/about/guidelines.xhtml

Figure 2. Factors perceived as responsible for low birthweight in babies among the mothers of livebirths between October 2018 to September 2019 in the Indian state of Bihar. These perceptions are not mutually exclusive.

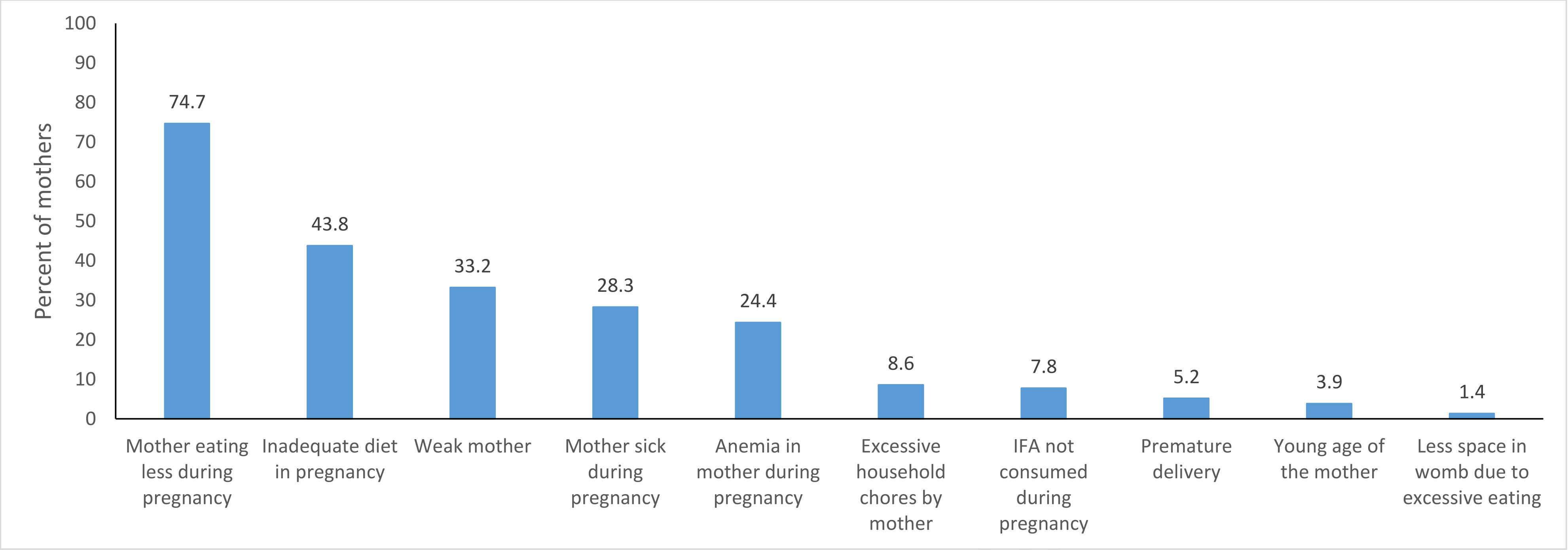

Supplementary Table 1. Prevalence of birthweight by categories, of no recall, and of child not being weighted at birth for select characteristics in the Indian state of Bihar for livebirths between October 2018 to September 2019.

	Prevalence per 100 livebirths (95% confidence interval)						
	**Birthweight >=2,500 g**	**Birthweight <2,500 g**	***Birthweight 2,000 - 2,499 g***	***Birthweight 1,500 - 1,999 g***	***Birthweight <1,500 g***	**Mother could not recall birthweight**	**Child not weighted at birth**
Overall	59.3 (57.9-60.6)	13.3 (12.4-14.3)	*9.3 (8.5-10.1)*	*3.0 (2.6-3.5)*	*1.1 (0.8-1.4)*	5.8 (5.2-6.5)	21.5 (20.4-22.7)
**Maternal age**[*]							
15-19 years	56.1 (51.9-60.3)	20.8 (17.5-24.5)	*15.3 (12.5-18.6)*	*4.7 (3.2-6.9)*	*0.8 (0.3-2.0)*	6.0 (4.3-8.4)	17.0 (14.0-20.5)
20-24 years	61.6 (59.6-63.5)	14.0 (12.7-15.5)	*9.8 (8.7-11.1)*	*3.1 (2.4-3.8)*	*1.1 (0.8-1.6)*	5.1 (4.3-6.1)	19.3 (17.7-20.9)
25-29 years	60.8 (58.3-63.3)	9.9 (8.5-11.6)	*6.5 (5.4-7.9)*	*2.3 (1.7-3.3)*	*1.0 (0.6-1.7)*	6.5 (5.4-7.9)	22.7 (20.6-24.9)
>=30 years	49.9 (46.0-53.8)	12.0 (9.7-14.8)	*8.5 (6.6-11.0)*	*2.7 (1.7-4.3)*	*0.8 (0.3-1.9)*	6.5 (4.8-8.7)	31.6 (28.1-35.3)
**Maternal education**[§]							
No education	48.3 (46.1-50.6)	13.1 (11.7-14.7)	*8.6 (7.4-9.9)*	*3.1 (2.5-4.0)*	*1.4 (0.9-2.0)*	8.0 (6.8-9.3)	30.6 (28.5-32.7)
Class 1 to 5	56.7 (53.2-60.2)	14.9 (12.5-17.6)	*10.4 (8.4-12.8)*	*3.7 (2.6-5.3)*	*0.8 (0.4-1.7)*	5.7 (4.2-7.5)	22.8 (19.9-25.9)
More than class 5	69.1 (67.2-70.9)	12.9 (11.6-14.4)	*9.5 (8.4-10.8)*	*2.6 (2.0-3.3)*	*0.8 (0.5-1.3)*	4.1 (3.4-5.0)	13.8 (12.5-15.3)
**Wealth index quartile**[#]							
I	47.6 (44.9-50.4)	14.3 (12.4-16.3)	*9.5 (8.0-11.2)*	*3.6 (2.7-4.8)*	*1.2 (0.7-2.0)*	6.1 (4.9-7.6)	32.0 (29.4-34.6)
II	54.3 (51.6-57.1)	14.3 (12.4-16.3)	*9.6 (8.1-11.4)*	*3.3 (2.5-4.5)*	*1.3 (0.8-2.1)*	7.3 (5.9-8.8)	24.1 (21.9-26.6)
III	62.0 (59.3-64.6)	13.3 (11.5-15.3)	*10.0 (8.4-11.7)*	*2.4 (1.7-3.4)*	*1.0 (0.5-1.7)*	7.0 (5.7-8.6)	17.7 (15.7-19.9)
IV	73.1 (70.6-75.5)	11.6 (9.9-13.4)	*8.1 (6.7-9.8)*	*2.6 (1.9-3.7)*	*0.8 (0.4-1.5)*	2.9 (2.1-4.0)	12.4 (10.7-14.4)
**Sex**							
Boy	62.3 (60.4-64.1)	11.9 (10.7-13.2)	*8.0 (7.0-9.1)*	*2.9 (2.4-3.7)*	*1.0 (0.6-1.4)*	5.6 (4.8-6.5)	20.2 (18.7-21.8)
Girl	56.0 (54.1-58.0)	14.9 (13.5-16.4)	*10.7 (9.5-12.0)*	*3.0 (2.4-3.8)*	*1.2 (0.8-1.7)*	6.1 (5.2-7.1)	23.0 (21.3-24.7)
**Gestation period§**							
6-7 months	8.7 (3.3-21.2)	63.0 (48.2-75.8)	*17.4 (8.9-31.3)*	*26.1 (15.4-40.7)*	*19.6 (10.4-33.7)*	8.7 (3.3-21.2)	19.6 (10.4-33.7)
8 months	55.4 (52.2-58.5)	18.9 (16.5-21.5)	*13.1 (11.1-15.4)*	*4.3 (3.2-5.8)*	*1.4 (0.8-2.4)*	5.4 (4.1-7.0)	20.3 (17.9-23.0)
>8 months	60.8 (59.3-62.3)	11.4 (10.5-12.4)	8.3 (7.5-9.2)	2.4 (2.0-2.9)	0.7 (0.5-1.0)	5.9 (5.2-6.7)	21.9 (20.6-23.2)
**Birth order**							
1st	63.5 (60.9-66.0)	17.8 (15.8-19.9)	12.5 (10.9-14.4)	4.2 (3.3-5.5)	1.0 (0.6-1.7)	5.1 (4.0-6.3)	13.7 (12.0-15.6)
2nd	62.1 (59.5-64.6)	12.3 (10.7-14.2)	8.9 (7.5-10.5)	2.4 (1.7-3.4)	1.0 (0.6-1.7)	5.8 (4.7-7.1)	19.8 (17.8-22.0)
$>2^{nd}$	55.2 (53.2-57.2)	11.2 (9.9-12.5)	7.6 (6.6-8.8)	2.5 (2.0-3.3)	1.0 (0.7-1.5)	6.3 (5.4-7.4)	27.3 (25.5-29.2)

	Prevalence per 100 livebirths (95% confidence interval)						
	**Birthweight >=2,500 g**	**Birthweight <2,500 g**	***Birthweight 2,000 - 2,499 g***	***Birthweight 1,500 - 1,999 g***	***Birthweight <1,500 g***	**Mother could not recall birthweight**	**Child not weighted at birth**
**Place of delivery§**							
Public sector facility	74.8 (73.2-76.4)	16.6 (15.2-18.0)	*12.1 (10.9-13.3)*	*3.3 (2.7-4.0)*	*1.2 (0.8-1.7)*	7.3 (6.4-8.3)	1.3 (1.0-1.8)
Private sector facility	71.1 (68.3-73.8)	15.9 (13.8-18.3)	*9.9 (8.2-11.9)*	*4.7 (3.6-6.2)*	*1.4 (0.8-2.3)*	7.1 (5.7-8.9)	5.8 (4.5-7.4)
Home	9.2 (7.6-11.0)	2.6 (1.8-3.7)	*1.7 (1.1-2.6)*	*0.6 (0.3-1.3)*	*0.3 (0.1-0.8)*	0.8 (0.4-1.5)	87.5 (85.4-89.3)
**Current status of livebirth**							
Died on day 0 of birth	28.1 (17.9-41.1)	17.5 (9.7-29.8)	*5.3 (1.7-15.2)*	*8.8 (3.7-19.5)*	*3.5 (0.9-13.1)*	14.0 (7.1-25.8)	40.4 (28.4-53.6)
Died between day 1-27 of birth	37.9 (26.4-51.1)	31.0 (20.4-44.1)	*12.1 (5.8-23.8)*	*12.1 (5.8-23.3)*	*6.9 (2.6-17.1)*	*12.1 (5.8-23.3)*	*19.0 (10.8-31.2)*
Died between day 28 and 11 months of age	37.1 (22.8-54.2)	25.7 (13.8-42.8)	*8.6 (2.7-23.8)*	*11.4 (4.3-27.1)*	*5.7 (1.4-20.5)*	*0*	*37.1 (22.8-54.2)*
Alive	60.1 (58.7-61.4)	13.0 (12.1-14.0)	*9.3 (8.5-10.2)*	*2.8 (2.3-3.2)*	*0.9 (0.7-1.2)*	5.7 (5.1-6.4)	21.2 (20.1-22.4)

*Data not available for 14 livebirths
§Data not available for 4 livebirths
Data not available for 1 livebirth

For peer review only

Supplementary Table 2. Association of low birth weight (LBW) among babies with birth weight available, and of not being weighted at birth among all livebirths with select variables using multiple logistic regression for livebirths between October 2018 to September 2019 in the Indian state of Bihar.

	<2,500 g birthweight (LBW)			Not being weighted at birth		
Variables	Total N=3,647 (% of total)	% of livebirths with LBW	Odds ratio for having LBW (95% confidence interval)	Total N=5,021 (% of total)	% of livebirths not weighed at birth	Odds ratio for not being weighted at birth (95% confidence interval)
**Maternal age***						
15-19 years	407 (11.2)	110 (27.0)	1.00	529 (10.5)	90 (17.0)	1.00
20-24 years	1808 (49.7)	335 (18.5)	0.8 (0.6-1.0)	2392 (47.6)	461 (19.3)	1.0 (0.6-1.7)
25-29 years	1028 (28.3)	144 (14.0)	0.5 (0.4-0.8)	1453 (28.9)	330 (22.7)	0.9 (0.5-1.5)
>=30 years	392 (10.8)	76 (19.4)	0.7 (0.5-1.1)	633 (12.6)	200 (31.6)	1.3 (0.7-2.5)
**Maternal education**[#†]						
No education	1172 (32.2)	250 (21.3)	1.4 (1.1-1.8)	1907 (38.0)	583 (30.6)	1.8 (1.3-2.5)
Classes 1 to 5	544 (14.9)	113 (20.8)	1.3 (1.0-1.7)	760 (15.1)	173 (22.8)	1.3 (0.9-2.0)
More than class 5	1928 (52.9)	304 (15.8)	1.00	2350 (46.8)	325 (13.8)	1.00
**Wealth index quartile** ⊠						
I	777 (21.3)	179 (23.0)	1.8 (1.3-2.3)	1255 (25.0)	401 (32.0)	1.4 (0.9-2.1)
II	861 (23.6)	179 (20.8)	1.6 (1.2-2.1)	1255 (25.0)	303 (24.1)	1.1 (0.7-1.7)
III	945 (25.9)	167 (17.7)	1.3 (1.0-1.7)	1255 (25.0)	222 (17.7)	1.2 (0.8-1.8)
IV	1063 (29.2)	145 (13.6)	1.00	1255 (25.0)	156 (12.4)	1.00
**Sex**						
Boy	1939 (53.2)	311 (16.0)	1.00	2614 (52.1)	529 (20.2)	1.00
Girl	1708 (46.8)	359 (21.0)	1.4 (1.2-1.6)	2407 (47.9)	553 (23.0)	1.0 (0.8-1.3)
**Gestation period**[#]						
6-7 months	33 (0.9)	29 (87.9)	34.0 (11.6-99.6)	46 (0.9)	9 (19.6)	0.5 (0.1-1.9)
8 months	701 (19.2)	178 (25.4)	1.8 (1.5-2.3)	944 (18.8)	192 (20.3)	0.8 (0.6-1.2)

For peer review only

	<2,500 g birthweight (LBW)			Not being weighted at birth		
**Variables**	**Total N=3,647 (% of total)**	**% of livebirths with LBW**	**Odds ratio for having LBW (95% confidence interval)**	**Total N=5,021 (% of total)**	**% of livebirths not weighed at birth**	**Odds ratio for not being weighted at birth (95% confidence interval)**
>8 months	2910 (79.9)	460 (15.8)	1.00	4027 (80.3)	880 (21.9)	1.00
**Birth order#**						
1st	1110 (30.5)	243 (21.9)	1.00	1366 (27.2)	187 (13.7)	1.00
2nd	1019 (28.0)	169 (16.6)	0.8 (0.6-1.0)	1369 (27.3)	271 (19.8)	1.0 (0.6-1.5)
>2nd	1515 (41.6)	255 (16.8)	0.8 (0.6-1.0)	2282 (45.5)	623 (27.3)	1.1 (0.7-1.7)
**Place of delivery#§**						
Public sector facility	2622 (72.0)	475 (18.1)	1.00	2870 (57.2)	38 (1.3)	1.00
Private sector facility	890 (24.4)	163 (18.3)	1.0 (0.8-1.3)	1022 (20.4)	59 (5.8)	5.3 (3.5-8.1)
Home/on route	132 (3.6)	29 (22.0)	1.2 (0.8-1.8)	1125 (22.4)	984 (87.5)	532.2 (365.8-774.2)
**Current status of livebirth**						
Died on day 0 of birth	26 (0.7)	10 (38.5)	1.9 (0.8-4.5)	57 (1.1)	23 (40.4)	8.6 (3.6-20.5)
Died between day 1-27 of birth	40 (1.1)	18 (45.0)	1.8 (0.9-3.8)	58 (1.2)	11 (1.0)	0.7 (0.2-2.6)
Died between day 28 and 11 months of age	22 (0.6)	9 (40.9)	2.6 (1.1-6.4)	35 (0.7)	13 (37.1)	0.7 (0.2-2.7)
Alive	3559 (97.6)	633 (17.8)	1.00	4,871 (97.0)	1,035 (21.3)	1.00

*Data not available for 12 and 14 livebirths for LBW and not being weighted at birth, respectively
[illegible]-[illegible]alue <0.001, chi-square test of significance for both LBW and not being weighted at birth
§p-value= 0.536 for LBW and p-value <0.001 for not being weighted at birth, chi-square test of significance
#Data not available for 3 and 4 livebirths for LBW and not being weighted at birth, respectively
[illegible]ata not available for 1 livebirth for both LBW and not being weighted at birth

For peer review only

Supplementary Figure 1. Distribution of birthweight values of 2500 g, 3,000 g, and 3,500 g by select variables among the livebirths born between October 2018 to September 2019 for whom birthweight was available in the Indian state of Bihar.

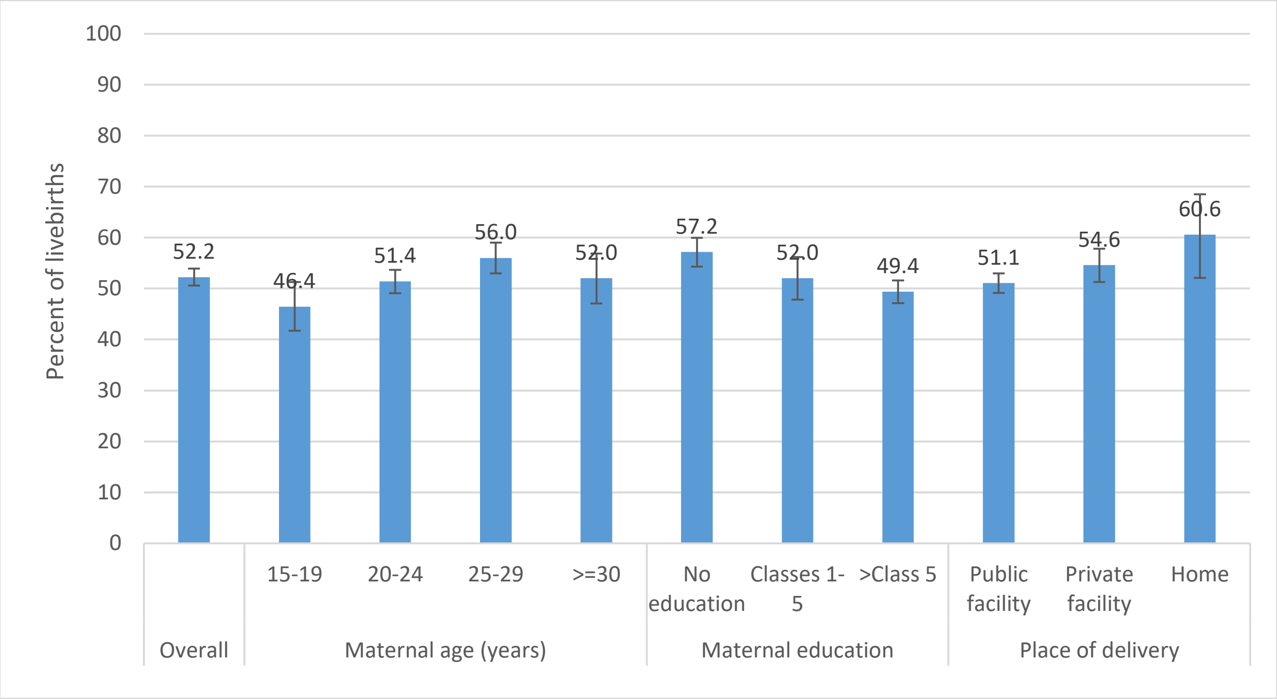

STROBE 2007 (v4) Statement—Checklist of items that should be included in reports of *cross-sectional studies*

Section/Topic	Item #	Recommendation	Reported on page #
**Title and abstract**	1	(*a*) Indicate the study's design with a commonly used term in the title or the abstract	2
		(*b*) Provide in the abstract an informative and balanced summary of what was done and what was found	2
**Introduction**			
Background/rationale	2	Explain the scientific background and rationale for the investigation being reported	4
Objectives	3	State specific objectives, including any pre-specified hypotheses	4
**Methods**			
Study design	4	Present key elements of study design early in the paper	5
Setting	5	Describe the setting, locations, and relevant dates, including periods of recruitment, exposure, follow-up, and data collection	5
Participants	6	(*a*) Give the eligibility criteria, and the sources and methods of selection of participants	5
Variables	7	Clearly define all outcomes, exposures, predictors, potential confounders, and effect modifiers. Give diagnostic criteria, if applicable	6
Data sources/ measurement	8*	For each variable of interest, give sources of data and details of methods of assessment (measurement). Describe comparability of assessment methods if there is more than one group	6
Bias	9	Describe any efforts to address potential sources of bias	
Study size	10	Explain how the study size was arrived at	5
Quantitative variables	11	Explain how quantitative variables were handled in the analyses. If applicable, describe which groupings were chosen and why	6
Statistical methods	12	(*a*) Describe all statistical methods, including those used to control for confounding	6
		(*b*) Describe any methods used to examine subgroups and interactions	6-7
		(*c*) Explain how missing data were addressed	Tables 1 and 2
		(*d*) If applicable, describe analytical methods taking account of sampling strategy	
		(*e*) Describe any sensitivity analyses	
**Results**			

For peer review only

Participants	13*	(a) Report numbers of individuals at each stage of study—eg numbers potentially eligible, examined for eligibility, confirmed eligible, included in the study, completing follow-up, and analysed	8
		(b) Give reasons for non-participation at each stage	
		(c) Consider use of a flow diagram	
Descriptive data	14*	(a) Give characteristics of study participants (eg demographic, clinical, social) and information on exposures and potential confounders	8
		(b) Indicate number of participants with missing data for each variable of interest	Tables 1 and 2
Outcome data	15*	Report numbers of outcome events or summary measures	8
Main results	16	(*a*) Give unadjusted estimates and, if applicable, confounder-adjusted estimates and their precision (eg, 95% confidence interval). Make clear which confounders were adjusted for and why they were included	8-12
		(*b*) Report category boundaries when continuous variables were categorized	Tables 1-3
		(*c*) If relevant, consider translating estimates of relative risk into absolute risk for a meaningful time period	
Other analyses	17	Report other analyses done—eg analyses of subgroups and interactions, and sensitivity analyses	12-13
**Discussion**			
Key results	18	Summarise key results with reference to study objectives	14
Limitations	19	Discuss limitations of the study, taking into account sources of potential bias or imprecision. Discuss both direction and magnitude of any potential bias	16
Interpretation	20	Give a cautious overall interpretation of results considering objectives, limitations, multiplicity of analyses, results from similar studies, and other relevant evidence	14-16
Generalisability	21	Discuss the generalisability (external validity) of the study results	14
**Other information**			
Funding	22	Give the source of funding and the role of the funders for the present study and, if applicable, for the original study on which the present article is based	18

*Give information separately for cases and controls in case-control studies and, if applicable, for exposed and unexposed groups in cohort and cross-sectional studies.

Note: An Explanation and Elaboration article discusses each checklist item and gives methodological background and published examples of transparent reporting. The STROBE checklist is best used in conjunction with this article (freely available on the Web sites of PLoS Medicine at http://www.plosmedicine.org/, Annals of Internal Medicine at http://www.annals.org/, and Epidemiology at http://www.epidem.com/). Information on the STROBE Initiative is available at www.strobe-statement.org.

For peer review only

BMJ Open

Implications of the availability and distribution of birthweight on addressing neonatal mortality: Population-based assessment from Bihar state of India

Journal:	*BMJ Open*
Manuscript ID	bmjopen-2022-061934.R1
Article Type:	Original research
Date Submitted by the Author:	06-May-2022
Complete List of Authors:	KUMAR, ANIL; Public Health Foundation of India,<br>George, Sibin; Public Health Foundation of India<br>Akbar, Md.; Public Health Foundation of India<br>Bhattacharya, Debarshi; Bill & Melinda Gates Foundation India<br>Nanda, Priya; Bill and Melinda Gates Foundation India<br>Dandona, Lalit; Public Health Foundation of India<br>Dandona, Rakhi; Public Health Foundation of India; University of Washington
**Primary Subject Heading**:	Epidemiology
Secondary Subject Heading:	Public health
Keywords:	PUBLIC HEALTH, Community child health < PAEDIATRICS, EPIDEMIOLOGY

SCHOLARONE™
Manuscripts

I, the Submitting Author has the right to grant and does grant on behalf of all authors of the Work (as defined in the below author licence), an exclusive licence and/or a non-exclusive licence for contributions from authors who are: i) UK Crown employees; ii) where BMJ has agreed a CC-BY licence shall apply, and/or iii) in accordance with the terms applicable for US Federal Government officers or employees acting as part of their official duties; on a worldwide, perpetual, irrevocable, royalty-free basis to BMJ Publishing Group Ltd ("BMJ") its licensees and where the relevant Journal is co-owned by BMJ to the co-owners of the Journal, to publish the Work in this journal and any other BMJ products and to exploit all rights, as set out in our licence.

The Submitting Author accepts and understands that any supply made under these terms is made by BMJ to the Submitting Author unless you are acting as an employee on behalf of your employer or a postgraduate student of an affiliated institution which is paying any applicable article publishing charge ("APC") for Open Access articles. Where the Submitting Author wishes to make the Work available on an Open Access basis (and intends to pay the relevant APC), the terms of reuse of such Open Access shall be governed by a Creative Commons licence – details of these licences and which Creative Commons licence will apply to this Work are set out in our licence referred to above.

Other than as permitted in any relevant BMJ Author's Self Archiving Policies, I confirm this Work has not been accepted for publication elsewhere, is not being considered for publication elsewhere and does not duplicate material already published. I confirm all authors consent to publication of this Work and authorise the granting of this licence.

For peer review only

May 2022

Implications of the availability and distribution of birthweight on addressing neonatal mortality: Population-based assessment from Bihar state of India

G. Anil Kumar[1], Sibin George[1], Md. Akbar[1], Debarshi Bhattacharya[3], Priya Nanda[3], Lalit Dandona[1,2], Rakhi Dandona[1,2]

[1]Public Health Foundation of India, Gurugram, National Capital Region, India

[2]Institute for Health Metrics and Evaluation, University of Washington, Seattle, USA

[3]Bill & Melinda Gates Foundation, India Country Office, New Delhi, India

Corresponding author: Prof. Rakhi Dandona, Public Health Foundation of India, Gurugram, Haryana – 122002, India; rakhi.dandona@phfi.org

Short title: Birthweight in Bihar

For peer review only - http://bmjopen.bmj.com/site/about/guidelines.xhtml

ABSTRACT

Objective: A large proportion of neonatal deaths in India are attributable to low birthweight (LBW). We report population-based distribution and determinants of birthweight in Bihar state, and on the perceptions about birthweight among carers.

Design: A cross-sectional household survey in a state representative sample of 6,007 livebirths born in 2018-2019. Mothers provided detailed interviews on sociodemographic characteristics and birthweight, and their perceptions on LBW (birthweight <2500 g). We report on birthweight availability, LBW prevalence, neonatal mortality rate (NMR) by birthweight, and perceptions of mothers on LBW implications.

Setting: Bihar state, India

Participants: Women with livebirth between October 2018 to September 2019

Results: A total of 5,021 (83.5%) livebirths participated, and 3,939 (78.4%) were weighed at birth. LBW prevalence among those with available birthweight was 18.4% (95% CI 17.1-19.7). Majority (87.5%) of the livebirths born at home were not weighed at birth. LBW prevalence decreased and birthweight >=2,500 g increased significantly with increasing wealth index quartile. NMR was significantly higher in livebirths weighing <1,500 g (11.3%; 95% CI 5.1-23.1) and 1,500-1,999 g (8.0%; 95% CI 4.6-13.6) than those weighing >=2,500 g (1.3%, 95% CI 0.9-1.7. Assuming proportional correspondence of LBW and NMR in livebirths with and without birthweight, the estimated LBW among those without birthweight was 35.5% (95% CI 33.0-38.0) and among all livebirths irrespective of birthweight availability was 23.0% (95% CI 21.9-24.2). Seventy percent of mothers considered LBW to be a sign of sickness, 59.5% perceived it as a risk of developing other illnesses, and 8.6% as having an increased probability of death.

Conclusions: Missing birthweight is substantially compromising the planning of interventions to address LBW at the population-level. Variations of LBW by place of delivery and socio-demographic indicators, and the perceptions of carers about LBW can facilitate appropriate actions to address LBW and the associated neonatal mortality.

For peer review only - http://bmjopen.bmj.com/site/about/guidelines.xhtml

STRENGTHS AND LIMITATIONS OF THE STUDY

- Data on birthweight documented for a representative sample of livebirths including neonatal deaths
- Documentation of birthweight based on recall, which are of reasonable quality based on the global criterion
- Perceptions of care-givers on low birthweight documented in the same population

For peer review only

For peer review only - http://bmjopen.bmj.com/site/about/guidelines.xhtml

INTRODUCTION

Global nutrition targets include a 30% reduction in low birthweight (LBW, weight less than 2500 g) prevalence between 2012 and 2030.(1) LBW is a significant indicator of not only maternal and fetal health predicting mortality and stunting, but also of adult-onset chronic conditions.(2-7) The global LBW prevalence was estimated at 14·6% in 2015,(8, 9) and short gestation for birthweight accounted for an estimated 1·43 million deaths and 139 million DALYs in 2017.(10)

South Asia, with India as its largest component, was estimated to have the highest LBW prevalence for any region in the world in 2015 as per the most recent global update on LBW prevalence which provided country-level estimates.(8, 9) However, LBW prevalence for India was not estimated in that report due to quality concerns with the available data.(8) We have reported LBW prevalence of 21·4% in India in 2017 as part of the Global Burden of Disease (GBD) Study,(11) and that 83% of neonatal deaths could be attributed to LBW in India in 2017.(12) LBW prevalence has shown modest decline over time in India, and it is projected that India is unlikely to meet the LBW national and global nutrition targets.(11) The inadequate availability and quality of birthweight data in India, like many low-income and middle-income countries, is a major hindrance in tracking LBW as a priority.(8, 9, 11)

In this background, we report on a population-based assessment of birthweight in the Indian state of Bihar, which is among the most populous Indian states accounting for a significant burden of neonatal mortality.(12) The LBW prevalence in Bihar was estimated as 23.4% in 2017 in the GBD study.(11) The aim of this report is to provide nuanced data for policy makers and program planners on the availability and distribution of birthweight, and implications of birthweight non-availability on robustness of LBW estimate which is of utmost significance in planning of interventions to reduce LBW in order to address neonatal mortality. Furthermore, we present the perceptions about LBW among the carers which can improve specificity of interventions to address LBW. We use data as is without smoothening or imputation in order to highlight for the policy makers the gaps in the birthweight data that are to be addressed for meaningful action.(8, 11)

For peer review only - http://bmjopen.bmj.com/site/about/guidelines.xhtml

METHODS

The ethics approval for this study was provided by the Institutional Ethics Committee of Public Health Foundation of India (Study number TRC-IEC 418/19). Written informed consent were obtained from all respondents who could read and write, and the information sheet and consent form were explained by the interviewer to those who could not read/write and their thumb impressions were obtained.

For the survey, a state representative sample of 6,000 livebirths was selected using a multistage sampling approach from 37 of the 38 districts of Bihar state, excluding the Lakhisarai district. In the first stage, 70 functioning community/primary health centres (CHC/PHC) were randomly sampled with probability proportional to population size from a total of 445 functioning CHC/PHCs, with each catering to an average of 84 villages. In the next stage, five villages were selected from the catchment area of each of the selected CHC/PHC using the village list available in the Census 2011.(13) To arrive at a cluster size of 300 households, villages with <300 households were combined with an adjacent village, and the large villages were split into equal-sized segments of 300 households using natural boundaries. In total, 350 clusters were sampled using a systematic sampling. Each selected cluster was mapped and all the households (a household was defined as people eating from the same kitchen) were enumerated to identify the livebirths delivered by women aged 15-49 years between October 2018 to September 2019.

The mother/care-giver of each identified livebirth was contacted for a detailed interview irrespective of whether the baby was currently alive. Details on the socio-demography, the pregnancy, delivery, and postnatal care of the eligible livebirth were documented. Specifically, for the analysis reported in this paper, birthweight was recorded from the mother or caregiver of the child based on their recall. We also documented the mother/caretaker's perception of the birthweight for each livebirth (very weak, weak, normal, overweight), and whether the mother/caretaker perceived low birthweight in a baby to be an indication of sickness, and if so why. Furthermore, the possible reasons for LBW in babies, how to prevent LBW, and if the mother/care-

For peer review only - http://bmjopen.bmj.com/site/about/guidelines.xhtml

taker thought if the delivery process was different based on the birthweight were also documented. The questionnaire was developed in English and then translated into Hindi (local language), after which it was back-translated into English to ensure the accurate and relevant meaning and intent of the questions. Pilot testing of the questionnaires was carried out and modifications made as necessary. Data were collected between November 2019 and January 2020 using Open Development Kit by interviewers trained in study procedures. Data entered were scrutinized using the internal consistency checks built in to detect and correct errors using standardised procedures to meet the data quality. To further improve data quality, spot checks were conducted by the supervisors in 10% of the households and back checks were done in 15% of the households. At least three attempts were made to reach out to all the eligible livebirths.

We tested the quality of birthweight data by using the criteria utilised for the report on the global LBW prevalence estimates.(8) Poor quality data was defined as extreme heaping with >55% of all birthweights falling on three values (2500 g, 3000 g, or 3500 g); >10% of births weighed at least 4500 g; or excessive heaping on the tail end of the birthweight distribution with more than 5% of birthweights at 250–500 g and 5500 g. We report on the quality of birthweight data, and for which livebirths the values of 2500 g, 3000 g, or 3500 g are more likely to be reported at the population-level.(8) We assessed the assumption if the data on child not weighted at birth was missing at random in this population using the run test of randomness.(14)

We categorised birthweight into five categories for analysis - <1,500 g, 1,500-1,999 g, 2,000-2,499 g, <2,500 g (LBW), and 2,500 g or more. We present birthweight prevalence per 100 livebirths for these five categories with 95% confidence intervals, and also for not being weighted at birth, and for birthweight could not be recalled considering all livebirths irrespective of birthweight availability. We then report birthweight prevalence for these five birthweight categories considering only the livebirths for whom birthweight was available. Among these, the prevalence and mean birthweight with standard deviation (SD) is also reported by maternal age, maternal education, wealth index, sex of the baby, length of the pregnancy, place of delivery, and based on livebirth survival. Wealth index

For peer review only - http://bmjopen.bmj.com/site/about/guidelines.xhtml

was estimated using the standard questions and methods used in the National Family Health Survey.(15) Multiple logistic regression was run to investigate the association of LBW among the livebirths with birthweight available with the above variables with all the variables introduced simultaneously in the model. Odds ratio with 95% CI are presented for the regression analysis.

We explored the association of neonatal and post-neonatal mortality with birthweight. Based on the difference in neonatal mortality rates between livebirths for whom birthweight was available versus those for whom birthweight was not available, we also report proportionately adjusted LBW prevalence in those with birthweight available to estimate the LBW prevalence in those with birthweight not available. In addition, a variety of perceptions of the caregivers about LBW are reported. All analysis was performed using STATA 13.1 software (Stata Corp, USA).

Patient and Public Involvement

Patients or the public were not involved in the design, or conduct, or reporting, or dissemination plans of our research.

For peer review only

RESULTS

We identified 6,007 livebirths representative of the Bihar state between October 2018 to September 2019 from 5,852 women aged 15-49 years in 55,475 households. Detailed interview was available for 5,021 (83.6%) livebirths, majority (98.2%) of whom were singleton births, 2,614 (52.1%) were boys, 2,870 (57.2 %) were born in a public health facility, and 150 (3%) were currently not alive. Of the 5,021 livebirths, 3,939 (78.4%) were weighed at birth but birthweight could not be recalled for 292 (7.4%, 95% CI 6.6-8.3) livebirths.

Quality of birthweight data

Considering the 3,647 livebirths with birthweight available, 52% of all birthweight values fell on 2,500 g, 3,000 g, or 3,500 g; 1.6% livebirths weighed at least 4,500 g; and 0.36% of birthweights were either at 250–500 g or 5,500 g. This indicates data to be of reasonable quality, as the heaping was less than the criteria for poor quality data. Significant variation was seen in the reporting of birthweight values of 2,500 g, 3,000 g, and 3,500 g by maternal age (chi-square, p=0.008), maternal education (chi-square, p<0.001), and place of delivery (chi-square, p=0.028) as shown in Supplementary Figure 1. The data on child not weighted at birth was not missing at random (z=0.22, p=0.820).

Distribution of birthweight among all livebirths

Considering all livebirths irrespective of birthweight availability, prevalence of birthweight >=2500 g was 59.3% (95% CI 57.9-60.6), of LBW was 13.3% (95% CI 12.4-14.3), and of livebirths not weighed at birth was 21.5% (95% CI 20.4-22.7) as shown in Supplementary Table 1. Importantly, the prevalence of livebirths not weighed at birth was 87.5 (95% CI 85.4-89.3) in home births as compared with only negligible facility births for whom birthweight was not measured (Supplementary Table 1).

Distribution of birthweight among livebirths with birthweight available

Among livebirths with birthweight available, the mean birthweight was 2,848.2 g with SD of ± 647.2 g (Table 1), and was significantly lower for livebirths born at 6-7 months of gestation (1,710.6 ± 577.4

For peer review only - http://bmjopen.bmj.com/site/about/guidelines.xhtml

g) and for livebirths of younger mothers aged <20 years (2,718.0 ± 642.5 g). Girls, livebirths belonging to lower wealth index quartile, and livebirths who did not survive were significantly more likely to have a lower mean birthweight as compared with boys, those belonging to higher wealth index quartile, and those currently alive, respectively (Table 1).

Table 1. Mean birthweight for livebirths between October 2018 to September 2019 for whom birthweight could be recalled in the Indian state of Bihar.

	Total	Availability of birth weight (% of total)	Mean birthweight (g)
**Overall**	**5,021**	**3,647 (72.6)**	**2,848.2 ± 647.2**
**Maternal age *†**			
15-19 years	529	407 (76.9)	2,718.0 ± 642.5
20-24 years	2,392	1,808 (75.6)	2,836.6 ± 646.3
25-29 years	1,453	1,028 (70.8)	2,911.8 ± 632.8
>=30 years	633	392 (61.9)	2,878.7 ± 662.5
**Maternal education§†**			
No education	1,907	1,172 (61.5)	2,801.0 ± 685.6
Classes 1 to 5	760	544 (71.6)	2,826.0 ± 664.4
More than class 5	2,350	1,928 (82.0)	2,885.4 ± 613.3
**Wealth index quartile#†**			
I	1,255	777 (61.9)	2,781.9 ± 690.1
II	1,255	861 (68.6)	2,800.7 ± 656.0
III	1,255	945 (75.3)	2,879.9 ± 659.2
IV	1,255	1,063 (84.7)	2,907.0 ± 588.0
**Sex‡**			
Boy	2,614	1,939 (74.2)	2,888.7 ± 647.1
Girl	2,407	1,708 (71.0)	2,802.3 ± 644.3
**Gestation period†**			
6-7 months	46	33 (71.7)	1,710.6 ± 577.4
8 months	944	701 (74.3)	2,735.7 ± 631.7
>8 months	4,027	2,910 (72.3)	2,889.7 ± 635.2
**Birth order†**			
1st	1,366	1,110 (81.3)	2,775.2 ± 628.5
2nd	1,369	1,019 (74.4)	2,892.5 ± 653.1
>2nd	2,282	1,515 (66.4)	2,874.8 ± 649.8
**Place of delivery§†**			
Public sector facility	2,870	2,622 (91.4)	2,839.3 ± 625.9
Private sector facility	1,022	890 (87.1)	2,880.7 ± 697.0
Home	1,125	132 (11.7)	2,839.2 ± 679.6
**Current status of livebirth‡**			
Died on day 0 of birth	57	26 (45.6)	2,644.2 ± 1,082.1
Died between day 1-27 of birth	58	40 (69.0)	2,611.3 ± 1,071.3
Died between day 28 and 11 months of age	35	22 (62.9)	2,368.2 ± 771.9
Alive	4,871	3559 (73.1)	2,855.3 ± 634.4

For peer review only

*Data not available for 14 livebirths
†Chi-square test of significance, p-value <0.001
§Data not available for 4 livebirths
#Data not available for 1 livebirth
‡Chi-square test of significance, p-value =0.001

The prevalence of LBW was 18.4 (95% CI 17.1-19.7), and that of birthweight <1,500 g was 1.5 (95% CI 1.1-1.9), of 1,500-1,999 g was 4.1 (95% CI 3.5-4.8), and of 2,000-2,400 g was 12.8 (95% CI 11.8-13.9) as shown in Table 2. LBW prevalence was 5.6 times higher among the babies who were born with 6-7 months of gestation as compared with those born >8 months of gestation (Table 2 and Figure 1). LBW prevalence decreased and that for birthweight >=2,500 g increased significantly (p<0.001) with increasing wealth index quartile (Table 2 and Figure 1). Using multiple logistic regression (Supplementary Table 2), the most significant odds of having LBW were for livebirths with gestation period of 6-7 months (OR 34.0; 95% CI 11.6-99.6).

Table 2. Prevalence of birthweight by categories among the livebirths who had birthweight available for select characteristics in the Indian state of Bihar for livebirths between October 2018 to September 2019.

	Prevalence per 100 livebirths (95% confidence interval)				
	**Birthweight >=2,500 g**	**Birthweight <2,500 g**	***Birthweight 2,000 - 2,499 g***	***Birthweight 1,500 - 1,999 g***	***Birthweight <1,500 g***
Overall	81.6 (80.3-82.9)	18.4 (17.1-19.7)	*12.8 (11.8-13.9)*	*4.1 (3.5-4.8)*	*1.5 (1.1-1.9)*
**Maternal age***					
15-19 years	73.0 (68.4-77.1)	27.0 (22.9-31.6)	*19.9 (16.3-24.1)*	*6.1 (4.2-8.9)*	*1.0 (0.4-2.6)*
20-24 years	81.5 (79.6-83.2)	18.5 (16.8-20.4)	*13.0 (11.5-14.6)*	*4.0 (3.2-5.1)*	*1.5 (1.0-2.2)*
25-29 years	86.0 (83.7-88.0)	14.0 (12.0-16.3)	*9.2 (7.6-11.2)*	*3.3 (2.4-4.6)*	*1.5 (0.9-2.4)*
>=30 years	80.6 (76.4-84.2)	19.4 (15.8-23.6)	*13.8 (10.7-17.6)*	*4.3 (2.7-6.9)*	*1.3 (0.5-3.0)*
**Maternal education§**					
No education	78.7 (76.2-80.9)	21.3 (19.1-23.8)	*14.0 (12.1-16.1)*	*5.1 (4.0-6.5)*	*2.2 (1.5-3.2)*
Class 1 to 5	79.2 (75.6-82.4)	20.8 (17.6-24.4)	*14.5 (11.8-17.7)*	*5.2 (3.6-7.4)*	*1.1 (0.5-2.4)*
More than class 5	84.2 (82.5-85.8)	15.8 (14.2-17.5)	*11.6 (10.3-13.1)*	*3.2 (2.5-4.1)*	*1.0 (0.6-1.5)*
**Wealth index quartile #**					
I	78.7 (76.2-80.9)	23.0 (20.2-26.1)	*15.3 (13.0-18.0)*	*5.8 (4.4-7.7)*	*1.9 (1.2-3.2)*
II	79.2 (75.6-82.4)	20.8 (18.2-23.6)	*14.1 (11.9-16.5)*	*4.9 (3.6-6.5)*	*1.9 (1.1-3.0)*
III	84.2 (82.5-85.8)	17.7 (15.4-20.2)	*13.2 (11.2-15.5)*	*3.2 (2.2-4.5)*	*1.3 (0.7-2.2)*
IV	78.7 (76.2-80.9)	13.6 (11.7-15.8)	*9.6 (8.0-11.5)*	*3.1 (2.2-4.3)*	*0.9 (0.5-1.7)*
**Sex**					
Boy	84.0 (82.3-85.5)	16.0 (14.5-17.7)	*10.8 (9.5-12.2)*	*4.0 (3.2-4.9)*	*1.3 (0.9-1.9)*
Girl	79.0 (77.0-80.9)	21.0 (19.2-23.0)	*15.1 (13.5-16.9)*	*4.3 (3.4-5.3)*	*1.6 (1.1-2.4)*

For peer review only - http://bmjopen.bmj.com/site/about/guidelines.xhtml

	Prevalence per 100 livebirths (95% confidence interval)				
	**Birthweight >=2,500 g**	**Birthweight <2,500 g**	***Birthweight 2,000 - 2,499 g***	***Birthweight 1,500 - 1,999 g***	***Birthweight <1,500 g***
**Gestation period§**					
6-7 months	12.1 (4.6-28.5)	87.9 (71.5-95.5)	*24.2 (12.5-41.8)*	*36.4 (21.8-54.0)*	*27.3 (14.7-45.0)*
8 months	74.6 (71.3-77.7)	25.4 (22.3-28.8)	*17.7 (15.0-20.7)*	*5.9 (4.3-7.9)*	*1.9 (1.1-3.2)*
>8 months	84.2 (82.8-85.5)	15.8 (14.5-17.2)	*11.5 (10.4-12.7)*	*3.3 (2.7-4.0)*	*1.0 (0.7-1.4)*
**Birth order**					
1st	78.1 (75.6-80.5)	21.9 (19.6-24.4)	*15.4 (13.4-17.7)*	*5.2 (4.1-6.7)*	*1.3 (0.8-2.1)*
2nd	83.4 (81.0-85.6)	16.6 (14.4-19.0)	*12.0 (10.1-14.1)*	*3.2 (2.3-4.5)*	*1.4 (0.8-2.3)*
>2nd	83.2 (81.2-85.0)	16.8 (15.0-18.8)	*11.5 (10.0-13.2)*	*3.8 (3.0-4.9)*	*1.5 (1.0-2.3)*
**Place of delivery§**					
Public sector facility	81.9 (80.4-83.3)	18.1 (16.7-19.6)	*13.2 (12.0-14.6)*	*3.6 (2.9-4.4)*	*1.3 (0.9-1.8)*
Private sector facility	81.7 (79.0-84.1)	18.3 (15.9-21.0)	*11.4 (9.4-13.6)*	*5.4 (4.1-7.1)*	*1.6 (0.9-2.6)*
Home	78.0 (70.1-84.3)	22.0 (15.7-29.9)	*14.4 (9.4-21.5)*	*5.3 (2.5-10.7)*	*2.3 (0.7-6.8)*
**Current status of livebirth**					
Died on day 0 of birth	61.5 (41.7-78.2)	38.5 (21.8-58.3)	*11.5 (3.7-30.8)*	*19.2 (8.1-39.2)*	*7.7 (1.9-26.6)*
Died between day 1-27 of birth	55.0 (39.4-69.7)	45.0 (30.3-60.6)	*17.5 (8.5-32.6)*	*17.5 (8.5-32.6)*	*10.0 (3.8-24.0)*
Died between day 28 and 11 months of age	59.1 (37.7-77.5)	40.9 (22.5-62.3)	*13.6 (4.3-35.5)*	*18.2 (6.8-40.3)*	*9.1 (2.2-30.7)*
Alive	82.2 (80.9-83.4)	17.8 (16.6-19.1)	*12.8 (11.7-13.9)*	*3.8 (3.2-4.4)*	*1.3 (1.0-1.7)*

Of the 670 LBW babies, the parents of 463 (69.1%) livebirths were informed by the health provider that the baby was weak/LBW. This proportion was 87.2% for the 203 livebirths with birthweight of <2,000 g and 94.3% for 53 livebirths with birthweight of <1,500 g. Considering the 190 facility livebirths with birthweight <2,000 g, livebirths at public facility (84%) were significantly less likely to be informed by the health provider of the baby being weak/having LBW as compared with those born in a private sector facility (93.6%; Z test for significance p<0.1).

Mortality and birthweight

A total of 150 (3.0%) livebirths were not currently alive) of whom 114 (76%) had died during the neonatal period (Table 1). The neonatal mortality rate in livebirths weighing <1,500 g (11.3%; 95% CI 5.1-23.1) and 1,500-1,999 g (8.0%; 95% CI 4.6-13.6) was significantly higher than in those weighing >=2,500 g (Table 3). The neonatal mortality rate in livebirths for whom birthweight was not available (3.5; 95% CI 2.6-4.6) was almost twice as high as compared with those for whom birthweight was

For peer review only

available (1.8%, 95% CI1.4-2.3) as shown in Table 3. Based on this 93% higher neonatal mortality rate among livebirths for whom birthweight was not available, and assuming a direct correspondence between neonatal mortality rate and LBW, we estimated that LBW among livebirths for whom birthweight was not available would be 35.5% (95% CI 33.0-38.0), that is, 93% higher than the 18.4% LBW among livebirths for whom birthweight was available. Based on the proportions of these two groups among all livebirths, we estimated an overall LBW of 23.0% (95% CI 21.9-24.2) among all livebirths.

Table 3. Mortality by birthweight categories among the livebirths born between October 2018 to September 2019 in the Indian state of Bihar. CI denotes confidence interval.

Birthweight	Number of livebirths	Number of neonatal deaths	Neonatal mortality rate, % (95% CI)	Number of deaths in post neonatal period to 11 months of age	Post-neonatal mortality rate to 11 months of age, % (95% CI)
>=2,500 g	2,977	38	1.3 (0.9-1.7)	13	0.4 (0.3-0.8)
<2,500 g	670	28	4.2 (2.9-6.0)	9	1.3 (0.7-2.6)
<1,500 g	*53*	*6*	*11.3 (5.1-23.1)*	*2*	*3.8 (0.9-14.0)*
1,500-1,999 g	*150*	*12*	*8.0 (4.6-13.6)*	*4*	*2.7 (1.0-6.9)*
2,000-2,499 g	*467*	*10*	*2.1 (1.2-3.9)*	*3*	*0.6 (0.2-2.0)*
**Birthweight available**	3,647	66	1.8 (1.4-2.3)	22	0.6 (0.4-0.9)
Not recalled	292	15	5.1 (3.1-8.4)	0	0
Not weighed at birth	1,082	33	3.0 (2.2-4.3)	14	1.3 (0.8-2.2)
**Birthweight not available**	1,374	48	3.5 (2.6-4.6)	14	1.0 (0.6-1.7)
**All livebirths**	**5,021**	**114**	**2.3 (1.9-2.7)**	**96**	**0.7 (0.5-1.0)**

Respondent's perceptions about LBW

Mothers were the predominant respondent in the survey (99.8%). Figure 2 shows the perception of mothers on the birthweight of their livebirth. Overall, 74.7% (3,748) of all mothers of livebirth, 88.1% (2,622) of mothers of livebirths >= 2,500g, and 25.5% (170) of mothers of LBW livebirths perceived their newborns to be of normal weight. Perception of weak or very weak was higher in LBW livebirths (73.3%) as compared with >= 2,500g livebirths (11%). Among the 53 livebirths with birthweight <1,500 g, 36 (67.9%) were perceived to be very weak, 9 (17%) weak and 6 (1.3%) of normal weight by the mother. These perceptions are not mutually exclusive.

A total of 3,527 (70.2%) mothers considered LBW to be a sign of sickness/illness. Among these 3,527 women, 2,988 (84.2%) perceived it as a risk of developing other illnesses, 1,764 (50%)

For peer review only - http://bmjopen.bmj.com/site/about/guidelines.xhtml

considered it a risk for weak growth, and 433 (12.3%) perceived it as having an increased probability of death (not mutually exclusive). Among the 1,350 (26.9%) women who did not consider LBW to be a sickness in a newborn, 1,308 (96.9%) felt that the baby would gain weight after birth and hence there was nothing to worry. Majority (4,570; 91%) of the mothers thought that LBW baby needed extra care; and the extra care practices commonly reported (not mutually exclusive) were oil massage (76.4%), exclusive breastfeeding (74.3%), seeking health care advice (46.6%), and keeping the baby warm (31.2%).

Figure 2 shows the possible reasons of LBW as reported by the mothers (not mutually exclusive). Mother eating less during pregnancy (74.7%), inadequate diet during pregnancy (43.8%), and weak mother (33.2%) were the most cited reasons for LBW baby. Majority of the mothers (94.9%) reported that intake of nutritious diet during pre- and during pregnancy can prevent LBW, followed by full antenatal care check-up (28.3%) and iron and folic acid intake (23.3%). A total of 3,026 (60.8%) mothers perceived the delivery process to be different depending on the birthweight of baby; 2,515 (83.1%) felt that delivery of LBW baby was easier than that of a normal weight baby, 891 (29.4%) thought that C-section was needed less for LBW babies, and 874 (28.9 %) felt that duration of labour was shorter for them (not mutually exclusive).

For peer review only

For peer review only - http://bmjopen.bmj.com/site/about/guidelines.xhtml

DISCUSSION

We present the estimates for birthweight prevalence across various categories in the Indian state of Bihar, including LBW prevalence which is essential for tracking progress towards the national and global nutrition targets. These estimates are presented in two ways – including and excluding livebirths based on birthweight availability – to highlight the need for improved birthweight availability to arrive at robust understanding of LBW prevalence for appropriate action both within the health system and the community. Socio-demographic distribution of livebirths for whom birthweight was not available can facilitate formulating specific actions in these populations to improve birthweight availability. Notably, the perceptions of mothers regarding reasons for LBW and its implications can provide a framework for developing relevant actions to improve care of LBW babies and possible actions to reduce LBW prevalence.

Birthweight was missing for 1 out of 4 livebirths in this population. Extrapolating our findings to the estimated 2.5 million livebirths in 2019 in Bihar, 543,000 livebirths were not weighted at birth and recall was not available for 146,600. Though home births accounted for only 22% of all livebirths in this population, these accounted for majority of the livebirths who were not weighted at birth. Therefore, until facility births can be increased further in the long-term that could result in increased birthweight measurement, tracking LBW as a priority target is not possible unless urgent targeted efforts are made in the short-term to engage with the health providers who assist with home births to improve birthweight availability.

Overall, birthweight data in our study was of reasonable quality as per the criteria used in the recent report on global estimation of LBW prevalence.(8) Unlike other reports,(8, 9) we did not smoothen the data for heaping, but have presented data as is to enhance understanding of where heaping was more likely to be reported to facilitate development of targeted approach in addressing this heaping. For the policy makers and program planners it is imperative to note where most action is needed to improve robustness of birthweight estimates. One of the assumptions made in the recent global report on LBW prevalence was that missing birthweights are missing at random and

For peer review only - http://bmjopen.bmj.com/site/about/guidelines.xhtml

that the true distribution of birthweights in a population can be approximated by a mixture of two normal distributions.(8) Our data has highlight that birthweight is not missing at random but in specific sub-groups, and this may be need to be taken into account in assumptions for global estimates.

The LBW prevalence estimated was 18.4% considering only livebirths with birthweight available, and 23% in all livebirths by proportionately adjusting for those who did not birthweight available based on their higher neonatal mortality rate. Even though the adjustment made for neonatal mortality is fairly simplistic, the extent of variation in LBW prevalence with this adjustment conveys the enormous implications of non-availability of birthweight for the planning of interventions and to appropriately allocate resources to address LBW at the population-level. Those without birthweight accounted for one-third of all neonatal deaths, and birthweight availability was less than half for the livebirths who had died on day 0. Importantly, the LBW prevalence was estimated to be almost twice among livebirths for whom birthweight was not available versus those for whom birthweight was available. This finding is of significance as we have previously reported that 50% of all neonatal mortality in the state to be in 0-2 days of birth, with 35% of them not weighted at birth.(16) Though the current study included only livebirths, our previous work in Bihar has also documented birthweight non-availability at 85% for stillbirths.(17) One of the proposed newborn quality of care indicator at health-facility level in low- and middle-income setting is facility neonatal mortality rate disaggregated by birth weight.(18) With majority of births now in the facilities, urgent and sustained effort is needed to track this quality indicator on a routine basis, which is currently not tracked in the Indian health information system. Interestingly, the Civil Registration System captures the birthweight for all births but that data is not available in public domain to comment on availability and quality of that data.(19) As LBW and short gestation are the predominant risk factors for neonatal mortality in India and in Bihar,(12) ensuring birthweight is measured for all livebirths irrespective of survival at birth is extremely important. Understanding the health providers perspectives on the need of birthweight measurement and quality is an

For peer review only - http://bmjopen.bmj.com/site/about/guidelines.xhtml

understudied issue,(20) and effort to improve this understanding is needed urgently to improve birthweight documentation.

A significant focus of neonatal health programs is on caring for the small and sick newborns, and communication with the carer/family is an integral part for their meaningful participation.(21) Seven in 10 carers of LBW babies were informed by the health provider that the baby was weak/LBW, and this proportion increased with decreasing birthweight. Some additional effort is needed in the public sector facilities as the families of babies born there were less likely to be informed than those in the private sector and informing birthweight and its implications by them to the family. Importantly, 70% of the mothers interviewed considered LBW to be a sign of sickness/illness, and such level of awareness could be translated not only into demand for availability of birthweight in the community, but also to increase uptake of relevant interventions for LBW babies.(22-27)

The finding of decrease in prevalence of LBW and increase in birthweight >=2,500 g with increasing wealth index quartile is not surprising, given that maternal undernutrition is associated with poor maternal-fetal outcomes including LBW.(2-6, 28) Despite decades of efforts in India to tackle malnutrition, it was the predominant risk factor for under-5 deaths in every state of India in 2017, accounting for 68·2% of the total under-5 deaths.(11) Globally, India has the highest prevalence of BMI lower than 16 in women, with less prevalence in women belonging to higher wealth index.(29) Evidence from Bangladesh suggests that low levels of women's empowerment are associated with maternal undernutrition as well as with delivering LBW babies, and empowerment is lower in women of lower wealth index.(28) What is noteworthy is that majority of the women in our study were well aware of the link between maternal nutrition and LBW, highlighting that facilitators are needed to translate this awareness into action to improve maternal nutrition, which can be achieved by bringing convergence of variety of nutrition-related activities of various government ministries and stakeholders for maternal health across the life cycle.(11, 30-34)

For peer review only - http://bmjopen.bmj.com/site/about/guidelines.xhtml

Documentation of birthweight based on recall in this study could be considered a limitation, however, these data were of reasonable quality using the global criterion.(8) The strengths of our study include an attempt to estimate LBW for all livebirths at the population level, and inclusion of carer perspectives in addition to birthweight availability that can facilitate actionable interventions or further implementation research to improve tracking of LBW, which is a priority global health indicator.

For peer review only

For peer review only - http://bmjopen.bmj.com/site/about/guidelines.xhtml

CONCLUSION

Significant efforts are needed in India beyond what is has been done so far to increase the availability and quality of birthweight in order to improve robustness of LBW estimates, which can help planning of appropriate interventions and investments to address this important risk factor of neonatal mortality. Without robust birthweight estimates, India may not able to address neonatal mortality effectively to meet the Sustainable Development Goal by 2030.

For peer review only

For peer review only - http://bmjopen.bmj.com/site/about/guidelines.xhtml

ACKNOWLEDGEMENTS

The authors acknowledge the contributions of Moutushi Majumder and Kaavya Singh from Public Health Foundation of India, and Asif Iqbal and Vipul Singhal from the Oxford Policy Management, India for data collection and data management.

AUTHORS' CONTRIBUTIONS

RD and GAK had full access to data in the study, take full responsibility for the integrity of data and accuracy of the data analysis, and had final responsibility for the decision to submit for publication; RD, GAK and LD conceptualized the study; RD guided the data analysis and drafted the manuscript; SG performed data analysis; MA guided data collection; MA, DB, PN and LD contributed to data analysis and interpretation; all authors approved the final manuscript.

FUNDING

This work was supported by Bill & Melinda Gates Foundation grant number INV-007989.

COMPETING INTEREST

PN and DB are employees of Bill & Melinda Gates Foundation. Other authors declare no completing interests.

DATA AVAILABILITY STATEMENT

All the data of the current study is available with the corresponding author, can be made available on request.

ETHICS APPROVAL

The ethics approval for this study was provided by the Institutional Ethics Committee of Public Health Foundation of India (Study number TRC-IEC 418/19).

For peer review only - http://bmjopen.bmj.com/site/about/guidelines.xhtml

REFERENCES

1. World Health Organization. Global nutrition monitoring framework: operational guidance for tracking progress in meeting targets for 2025. Geneva: WHO, 2017.
2. Katz J, Lee AC, Kozuki N, Lawn JE, Cousens S, Blencowe H, et al. Mortality risk in preterm and small-for-gestational-age infants in low-income and middle-income countries: a pooled country analysis. Lancet (London, England). 2013;382(9890):417-25.
3. Loret de Mola C, de França GV, Quevedo Lde A, Horta BL. Low birth weight, preterm birth and small for gestational age association with adult depression: systematic review and meta-analysis. The British journal of psychiatry : the journal of mental science. 2014;205(5):340-7.
4. Oudgenoeg-Paz O, Mulder H, Jongmans MJ, van der Ham IJM, Van der Stigchel S. The link between motor and cognitive development in children born preterm and/or with low birth weight: A review of current evidence. Neuroscience and biobehavioral reviews. 2017;80:382-93.
5. Shinzawa M, Tanaka S, Tokumasu H, Takada D, Tsukamoto T, Yanagita M, et al. Association of Low Birth Weight With Childhood Proteinuria at Age 3 Years: A Population-Based Retrospective Cohort Study. American journal of kidney diseases : the official journal of the National Kidney Foundation. 2019;74(1):141-3.
6. Victora CG, Adair L, Fall C, Hallal PC, Martorell R, Richter L, et al. Maternal and child undernutrition: consequences for adult health and human capital. Lancet (London, England). 2008;371(9609):340-57.
7. Wojcik W, Lee W, Colman I, Hardy R, Hotopf M. Foetal origins of depression? A systematic review and meta-analysis of low birth weight and later depression. Psychological medicine. 2013;43(1):1-12.
8. Blencowe H, Krasevec J, de Onis M, Black RE, An X, Stevens GA, et al. National, regional, and worldwide estimates of low birthweight in 2015, with trends from 2000: a systematic analysis. The Lancet Global health. 2019;7(7):e849-e60.
9. United Nations Children's Fund (UNICEF) and World Health Organization. UNICEF-WHO Low birthweight estimates: Levels and trends 2000–2015. Geneva: UNICEF and WHO, 2019.
10. GBD 2017 Risk Factor Collaborators. Global, regional, and national comparative risk assessment of 84 behavioural, environmental and occupational, and metabolic risks or clusters of risks for 195 countries and territories, 1990-2017: a systematic analysis for the Global Burden of Disease Study 2017. Lancet (London, England). 2018;392(10159):1923-94.
11. India State-Level Disease Burden Initiative Malnutrition Collaborators. The burden of child and maternal malnutrition and trends in its indicators in the states of India: the Global Burden of Disease Study 1990-2017. The Lancet Child & adolescent health. 2019;3(12):855-70.
12. India State-Level Disease Burden Initiative Child Mortality Collaborators. Subnational mapping of under-5 and neonatal mortality trends in India: the Global Burden of Disease Study 2000-17. Lancet (London, England). 2020;395(10237):1640-58.
13. Office of the Registrar General & Census Commissioner of India GoI, . C.D. Blockwise Primary Census Abstract Data (PCA) - Bihar: RGI; [cited 2021 23 June]. Available from: http://censusindia.gov.in/pca/cdb_pca_census/Houselisting-housing-BR.html.
14. Statistics Solutions. Run Test of Randomness: Statistics Solutions; 2022 [cited 2022 1 May]. Available from: https://www.statisticssolutions.com/free-resources/directory-of-statistical-analyses/run-test-of-randomness/.
15. International Institute for Population Sciences. About NFHS: IIPS; [cited 2021 20 August]. Available from: http://rchiips.org/nfhs/about.shtml.
16. Dandona R, Kumar GA, Bhattacharya D, Akbar M, Atmavilas Y, Nanda P, et al. Distinct mortality patterns at 0-2 days versus the remaining neonatal period: results from population-based assessment in the Indian state of Bihar. BMC medicine. 2019;17(1):140.

For peer review only - http://bmjopen.bmj.com/site/about/guidelines.xhtml

17. Dandona R, Kumar GA, Akbar M, Bhattacharya D, Nanda P, Dandona L. Deferred and referred deliveries contribute to stillbirths in the Indian state of Bihar: results from a population-based survey of all births. BMC medicine. 2019;17(1):28.
18. Madaj B, Smith H, Mathai M, Roos N, van den Broek N. Developing global indicators for quality of maternal and newborn care: a feasibility assessment. Bulletin of the World Health Organization. 2017;95(6):445-52I.
19. Office of the Registrar General & Census Commissioner of India GoI. Birth Report: RGI; [cited 2021 23 August]. Available from: https://censusindia.gov.in/2011-Documents/CRS_Forms/CRS%20Forms.pdf.
20. Gladstone ME, Salim N, Ogillo K, Shamba D, Gore-Langton GR, Day LT, et al. Birthweight measurement processes and perceived value: qualitative research in one EN-BIRTH study hospital in Tanzania. BMC pregnancy and childbirth. 2021;21(Suppl 1):232.
21. World Health Organization. Standards for improving the quality of care for small and sick newborns in health facilities. Geneva: WHO, 2020.
22. Koenraads M, Phuka J, Maleta K, Theobald S, Gladstone M. Understanding the challenges to caring for low birthweight babies in rural southern Malawi: a qualitative study exploring caregiver and health worker perceptions and experiences. BMJ global health. 2017;2(3):e000301.
23. Lydon M, Longwe M, Likomwa D, Lwesha V, Chimtembo L, Donohue P, et al. Starting the conversation: community perspectives on preterm birth and kangaroo mother care in southern Malawi. Journal of global health. 2018;8(1):010703.
24. Nisha MK, Raynes-Greenow C, Rahman A, Alam A. Perceptions and practices related to birthweight in rural Bangladesh: Implications for neonatal health programs in low- and middle-income settings. PloS one. 2019;14(12):e0221691.
25. Chan G, Bergelson I, Smith ER, Skotnes T, Wall S. Barriers and enablers of kangaroo mother care implementation from a health systems perspective: a systematic review. Health policy and planning. 2017;32(10):1466-75.
26. Mazumder S, Upadhyay RP, Hill Z, Taneja S, Dube B, Kaur J, et al. Kangaroo mother care: using formative research to design an acceptable community intervention. BMC public health. 2018;18(1):307.
27. Smith ER, Bergelson I, Constantian S, Valsangkar B, Chan GJ. Barriers and enablers of health system adoption of kangaroo mother care: a systematic review of caregiver perspectives. BMC pediatrics. 2017;17(1):35.
28. Kabir A, Rashid MM, Hossain K, Khan A, Sikder SS, Gidding HF. Women's empowerment is associated with maternal nutrition and low birth weight: evidence from Bangladesh Demographic Health Survey. BMC women's health. 2020;20(1):93.
29. Razak F, Corsi DJ, Slutsky AS, Kurpad A, Berkman L, Laupacis A, et al. Prevalence of Body Mass Index Lower Than 16 Among Women in Low- and Middle-Income Countries. Jama. 2015;314(20):2164-71.
30. Kim SS, Avula R, Ved R, Kohli N, Singh K, van den Bold M, et al. Understanding the role of intersectoral convergence in the delivery of essential maternal and child nutrition interventions in Odisha, India: a qualitative study. BMC public health. 2017;17(1):161.
31. McKerricher L, Petrucka P. Maternal nutritional supplement delivery in developing countries: a scoping review. BMC nutrition. 2019;5:8.
32. Noznesky EA, Ramakrishnan U, Martorell R. A situation analysis of public health interventions, barriers, and opportunities for improving maternal nutrition in Bihar, India. Food and nutrition bulletin. 2012;33(2 Suppl):S93-103.
33. Young MF, Bootwala A, Kachwaha S, Avula R, Ghosh S, Sharma PK, et al. Understanding Implementation and Improving Nutrition Interventions: Barriers and Facilitators of Using Data Strategically to Inform the Implementation of Maternal Nutrition in Uttar Pradesh, India. Current developments in nutrition. 2021;5(6):nzab081.

For peer review only - http://bmjopen.bmj.com/site/about/guidelines.xhtml

34. Ministry of Women and Child Development. Government of India. Administrative guidelines for implementation of National Nutrition Mission.: Ministry of Women and Child Development; 2018 [cited 2021 23 August]. Available from: https://icds-wcd.nic.in/nnm/NNM-Web-Contents/UPPER-MENU/AdministrativeApproval-Guidelines/Administrative_Guidelines_NNM-26022018.pdf.

For peer review only

For peer review only - http://bmjopen.bmj.com/site/about/guidelines.xhtml

FIGURES

Figure 1. Distribution of birthweight by the gestation period and wealth index quartile for livebirths between October 2018 to September 2019 for whom birthweight was available in the Indian state of Bihar.

Figure 2. Factors perceived as responsible for low birthweight in babies among the mothers of livebirths between October 2018 to September 2019 in the Indian state of Bihar.

SUPPLEMENTARY MATERIAL

Supplementary Figure 1. Distribution of birthweight values of 2500 g, 3,000 g, and 3,500 g by select variables among the livebirths born between October 2018 to September 2019 for whom birthweight was available in the Indian state of Bihar.

Supplementary Table 1. Prevalence of birthweight by categories, of no recall, and of child not being weighted at birth for select characteristics in the Indian state of Bihar for livebirths between October 2018 to September 2019.

Supplementary Table 2. Association of low birthweight (LBW) among babies with birthweight available with select variables using multiple logistic regression for livebirths between October 2018 to September 2019 in the Indian state of Bihar.

For peer review only

For peer review only - http://bmjopen.bmj.com/site/about/guidelines.xhtml

Figure 1. Distribution of birthweight by the gestation period and wealth index quartile for livebirths between October 2018 to September 2019 for whom birthweight was available in the Indian state of Bihar.

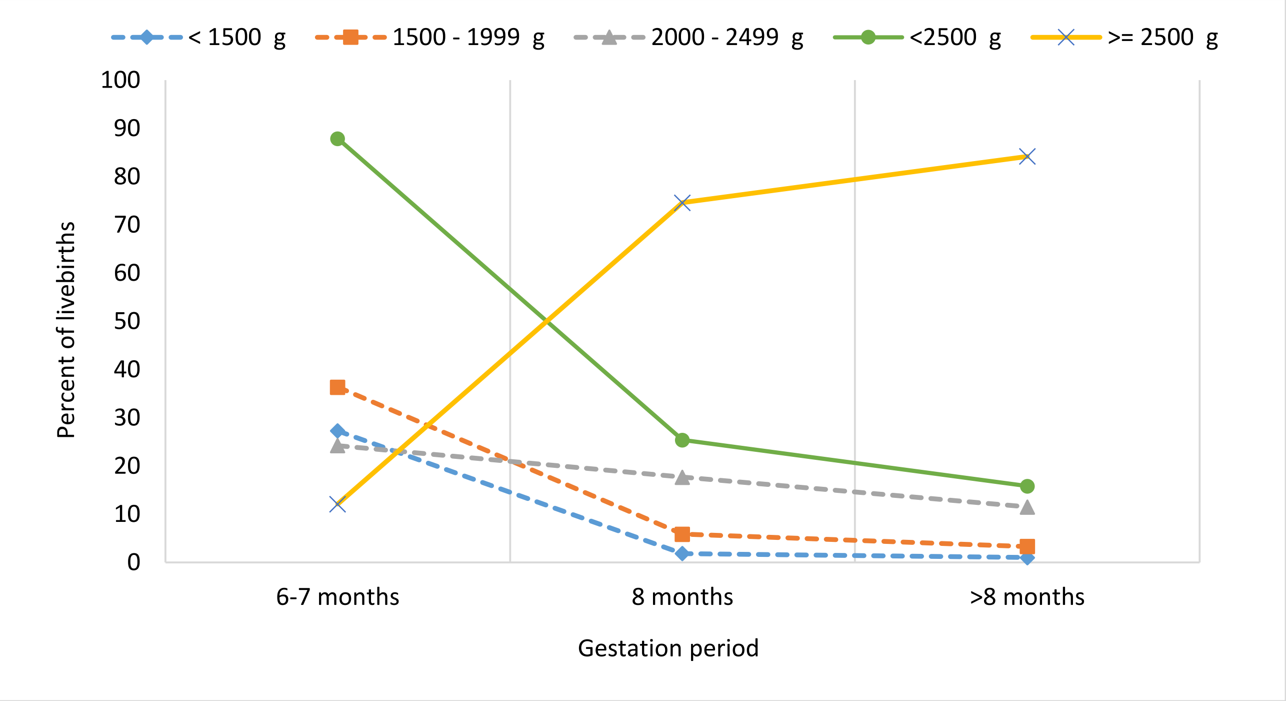

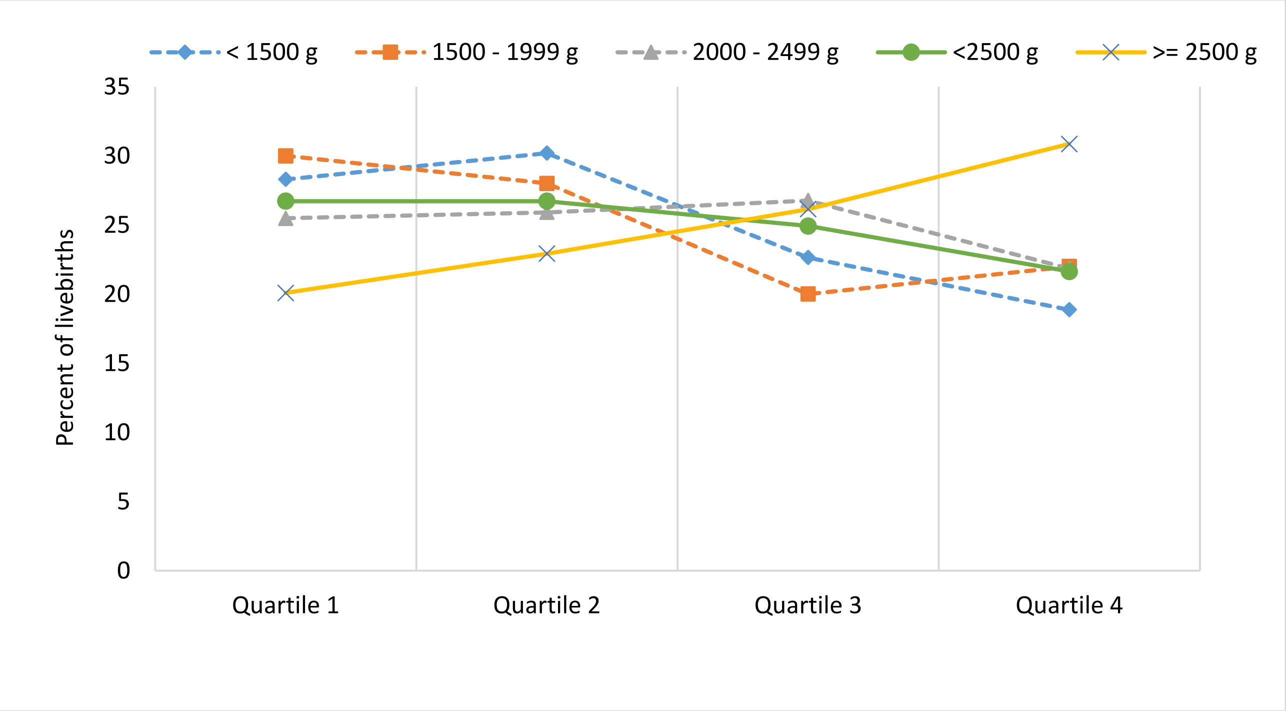

For peer review only - http://bmjopen.bmj.com/site/about/guidelines.xhtml

Figure 2. Factors perceived as responsible for low birthweight in babies among the mothers of livebirths between October 2018 to September 2019 in the Indian state of Bihar. These perceptions are not mutually exclusive.

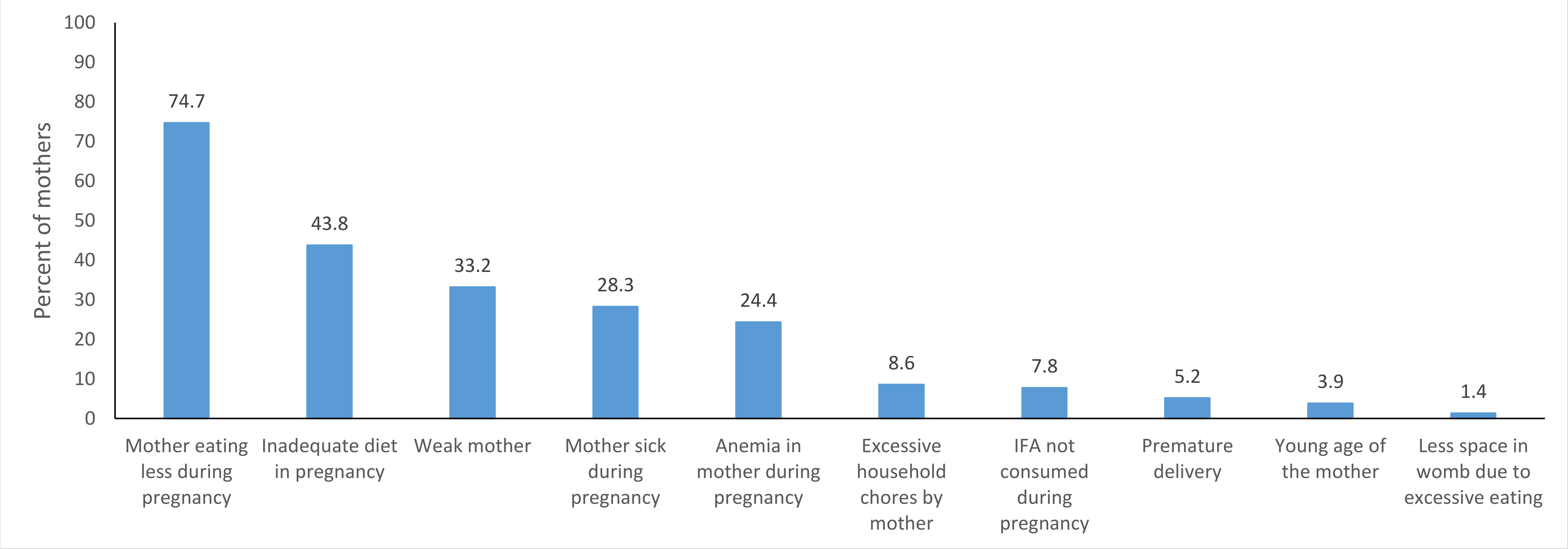

Supplementary Figure 1. Distribution of birthweight values of 2500 g, 3,000 g, and 3,500 g by select variables among the livebirths born between October 2018 to September 2019 for whom birthweight was available in the Indian state of Bihar.

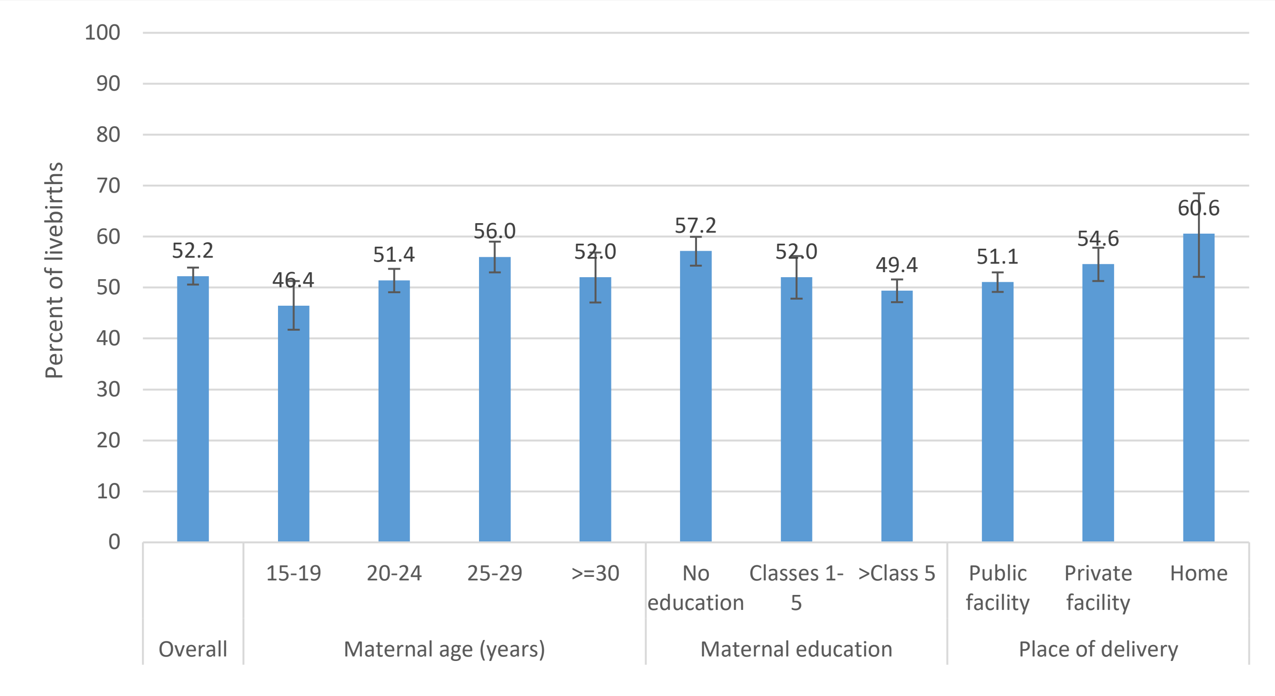

review only

Supplementary Table 1. Prevalence of birthweight by categories, of no recall, and of child not being weighted at birth for select characteristics in the Indian state of Bihar for livebirths between October 2018 to September 2019.

	Prevalence per 100 livebirths (95% confidence interval)						
	**Birthweight >=2,500 g**	**Birthweight <2,500 g**	***Birthweight 2,000 - 2,499 g***	***Birthweight 1,500 - 1,999 g***	***Birthweight <1,500 g***	**Mother could not recall birthweight**	**Child not weighted at birth**
Overall	59.3 (57.9-60.6)	13.3 (12.4-14.3)	*9.3 (8.5-10.1)*	*3.0 (2.6-3.5)*	*1.1 (0.8-1.4)*	5.8 (5.2-6.5)	21.5 (20.4-22.7)
**Maternal age***							
15-19 years	56.1 (51.9-60.3)	20.8 (17.5-24.5)	*15.3 (12.5-18.6)*	*4.7 (3.2-6.9)*	*0.8 (0.3-2.0)*	6.0 (4.3-8.4)	17.0 (14.0-20.5)
20-24 years	61.6 (59.6-63.5)	14.0 (12.7-15.5)	*9.8 (8.7-11.1)*	*3.1 (2.4-3.8)*	*1.1 (0.8-1.6)*	5.1 (4.3-6.1)	19.3 (17.7-20.9)
25-29 years	60.8 (58.3-63.3)	9.9 (8.5-11.6)	*6.5 (5.4-7.9)*	*2.3 (1.7-3.3)*	*1.0 (0.6-1.7)*	6.5 (5.4-7.9)	22.7 (20.6-24.9)
>=30 years	49.9 (46.0-53.8)	12.0 (9.7-14.8)	*8.5 (6.6-11.0)*	*2.7 (1.7-4.3)*	*0.8 (0.3-1.9)*	6.5 (4.8-8.7)	31.6 (28.1-35.3)
**Maternal education§**							
No education	48.3 (46.1-50.6)	13.1 (11.7-14.7)	*8.6 (7.4-9.9)*	*3.1 (2.5-4.0)*	*1.4 (0.9-2.0)*	8.0 (6.8-9.3)	30.6 (28.5-32.7)
Class 1 to 5	56.7 (53.2-60.2)	14.9 (12.5-17.6)	*10.4 (8.4-12.8)*	*3.7 (2.6-5.3)*	*0.8 (0.4-1.7)*	5.7 (4.2-7.5)	22.8 (19.9-25.9)
More than class 5	69.1 (67.2-70.9)	12.9 (11.6-14.4)	*9.5 (8.4-10.8)*	*2.6 (2.0-3.3)*	*0.8 (0.5-1.3)*	4.1 (3.4-5.0)	13.8 (12.5-15.3)
**Wealth index quartile #**							
I	47.6 (44.9-50.4)	14.3 (12.4-16.3)	*9.5 (8.0-11.2)*	*3.6 (2.7-4.8)*	*1.2 (0.7-2.0)*	6.1 (4.9-7.6)	32.0 (29.4-34.6)
II	54.3 (51.6-57.1)	14.3 (12.4-16.3)	*9.6 (8.1-11.4)*	*3.3 (2.5-4.5)*	*1.3 (0.8-2.1)*	7.3 (5.9-8.8)	24.1 (21.9-26.6)
III	62.0 (59.3-64.6)	13.3 (11.5-15.3)	*10.0 (8.4-11.7)*	*2.4 (1.7-3.4)*	*1.0 (0.5-1.7)*	7.0 (5.7-8.6)	17.7 (15.7-19.9)
IV	73.1 (70.6-75.5)	11.6 (9.9-13.4)	*8.1 (6.7-9.8)*	*2.6 (1.9-3.7)*	*0.8 (0.4-1.5)*	2.9 (2.1-4.0)	12.4 (10.7-14.4)
**Sex**							
Boy	62.3 (60.4-64.1)	11.9 (10.7-13.2)	*8.0 (7.0-9.1)*	*2.9 (2.4-3.7)*	*1.0 (0.6-1.4)*	5.6 (4.8-6.5)	20.2 (18.7-21.8)
Girl	56.0 (54.1-58.0)	14.9 (13.5-16.4)	*10.7 (9.5-12.0)*	*3.0 (2.4-3.8)*	*1.2 (0.8-1.7)*	6.1 (5.2-7.1)	23.0 (21.3-24.7)
**Gestation period§**							
6-7 months	8.7 (3.3-21.2)	63.0 (48.2-75.8)	*17.4 (8.9-31.3)*	*26.1 (15.4-40.7)*	*19.6 (10.4-33.7)*	8.7 (3.3-21.2)	19.6 (10.4-33.7)
8 months	55.4 (52.2-58.5)	18.9 (16.5-21.5)	*13.1 (11.1-15.4)*	*4.3 (3.2-5.8)*	*1.4 (0.8-2.4)*	5.4 (4.1-7.0)	20.3 (17.9-23.0)
>8 months	60.8 (59.3-62.3)	11.4 (10.5-12.4)	8.3 (7.5-9.2)	2.4 (2.0-2.9)	0.7 (0.5-1.0)	5.9 (5.2-6.7)	21.9 (20.6-23.2)
**Birth order**							
1st	63.5 (60.9-66.0)	17.8 (15.8-19.9)	12.5 (10.9-14.4)	4.2 (3.3-5.5)	1.0 (0.6-1.7)	5.1 (4.0-6.3)	13.7 (12.0-15.6)
2nd	62.1 (59.5-64.6)	12.3 (10.7-14.2)	8.9 (7.5-10.5)	2.4 (1.7-3.4)	1.0 (0.6-1.7)	5.8 (4.7-7.1)	19.8 (17.8-22.0)
>2nd	55.2 (53.2-57.2)	11.2 (9.9-12.5)	7.6 (6.6-8.8)	2.5 (2.0-3.3)	1.0 (0.7-1.5)	6.3 (5.4-7.4)	27.3 (25.5-29.2)

	Prevalence per 100 livebirths (95% confidence interval)						
	**Birthweight >=2,500 g**	**Birthweight <2,500 g**	***Birthweight 2,000 - 2,499 g***	***Birthweight 1,500 - 1,999 g***	***Birthweight <1,500 g***	**Mother could not recall birthweight**	**Child not weighted at birth**
**Place of delivery§**							
Public sector facility	74.8 (73.2-76.4)	16.6 (15.2-18.0)	*12.1 (10.9-13.3)*	*3.3 (2.7-4.0)*	*1.2 (0.8-1.7)*	7.3 (6.4-8.3)	1.3 (1.0-1.8)
Private sector facility	71.1 (68.3-73.8)	15.9 (13.8-18.3)	*9.9 (8.2-11.9)*	*4.7 (3.6-6.2)*	*1.4 (0.8-2.3)*	7.1 (5.7-8.9)	5.8 (4.5-7.4)
Home	9.2 (7.6-11.0)	2.6 (1.8-3.7)	*1.7 (1.1-2.6)*	*0.6 (0.3-1.3)*	*0.3 (0.1-0.8)*	0.8 (0.4-1.5)	87.5 (85.4-89.3)
**Current status of livebirth**							
Died on day 0 of birth	28.1 (17.9-41.1)	17.5 (9.7-29.8)	*5.3 (1.7-15.2)*	*8.8 (3.7-19.5)*	*3.5 (0.9-13.1)*	14.0 (7.1-25.8)	40.4 (28.4-53.6)
Died between day 1-27 of birth	37.9 (26.4-51.1)	31.0 (20.4-44.1)	*12.1 (5.8-23.8)*	*12.1 (5.8-23.3)*	*6.9 (2.6-17.1)*	*12.1 (5.8-23.3)*	*19.0 (10.8-31.2)*
Died between day 28 and 11 months of age	37.1 (22.8-54.2)	25.7 (13.8-42.8)	*8.6 (2.7-23.8)*	*11.4 (4.3-27.1)*	*5.7 (1.4-20.5)*	*0*	*37.1 (22.8-54.2)*
Alive	60.1 (58.7-61.4)	13.0 (12.1-14.0)	*9.3 (8.5-10.2)*	*2.8 (2.3-3.2)*	*0.9 (0.7-1.2)*	5.7 (5.1-6.4)	21.2 (20.1-22.4)

*Data not available for 14 livebirths
§Data not available for 4 livebirths
Data not available for 1 livebirth

For peer review only

Supplementary Table 2. Association of low birth weight (LBW) among babies with birth weight available with select variables using multiple logistic regression for livebirths between October 2018 to September 2019 in the Indian state of Bihar.

	<2,500 g birthweight (LBW)		
**Variables**	**Total N=3,647 (% of total)**	**% of livebirths with LBW**	**Odds ratio for having LBW (95% confidence interval)**
**Maternal age***			
15-19 years	407 (11.2)	110 (27.0)	1.00
20-24 years	1808 (49.7)	335 (18.5)	0.8 (0.6-1.0)
25-29 years	1028 (28.3)	144 (14.0)	0.5 (0.4-0.8)
>=30 years	392 (10.8)	76 (19.4)	0.7 (0.5-1.1)
**Maternal education[#†]**			
No education	1172 (32.2)	250 (21.3)	1.4 (1.1-1.8)
Classes 1 to 5	544 (14.9)	113 (20.8)	1.3 (1.0-1.7)
More than class 5	1928 (52.9)	304 (15.8)	1.00
**Wealth index quartile[‡]**			
I	777 (21.3)	179 (23.0)	1.8 (1.3-2.3)
II	861 (23.6)	179 (20.8)	1.6 (1.2-2.1)
III	945 (25.9)	167 (17.7)	1.3 (1.0-1.7)
IV	1063 (29.2)	145 (13.6)	1.00
**Sex**			
Boy	1939 (53.2)	311 (16.0)	1.00
Girl	1708 (46.8)	359 (21.0)	1.4 (1.2-1.6)
**Gestation period[#]**			
6-7 months	33 (0.9)	29 (87.9)	34.0 (11.6-99.6)
8 months	701 (19.2)	178 (25.4)	1.8 (1.5-2.3)
>8 months	2910 (79.9)	460 (15.8)	1.00
**Birth order[#]**			
1st	1110 (30.5)	243 (21.9)	1.00
2nd	1019 (28.0)	169 (16.6)	0.8 (0.6-1.0)
>2nd	1515 (41.6)	255 (16.8)	0.8 (0.6-1.0)
**Place of delivery[#§]**			
Public sector facility	2622 (72.0)	475 (18.1)	1.00
Private sector facility	890 (24.4)	163 (18.3)	1.0 (0.8-1.3)
Home/on route	132 (3.6)	29 (22.0)	1.2 (0.8-1.8)
**Current status of livebirth**			
Died on day 0 of birth	26 (0.7)	10 (38.5)	1.9 (0.8-4.5)
Died between day 1-27 of birth	40 (1.1)	18 (45.0)	1.8 (0.9-3.8)
Died between day 28 and 11 months of age	22 (0.6)	9 (40.9)	2.6 (1.1-6.4)
Alive	3559 (97.6)	633 (17.8)	1.00

For peer review only

*Data not available for 12 livebirths
†p-value <0.001, chi-square test of significance
§p-value= 0.536, chi-square test of significance
#Data not available for 3 livebirths
‡Data not available for 1 livebirth

For peer review only

STROBE 2007 (v4) Statement—Checklist of items that should be included in reports of *cross-sectional studies*

Section/Topic	Item #	Recommendation	Reported on page #
**Title and abstract**	1	(*a*) Indicate the study's design with a commonly used term in the title or the abstract	2
		(*b*) Provide in the abstract an informative and balanced summary of what was done and what was found	2
**Introduction**			
Background/rationale	2	Explain the scientific background and rationale for the investigation being reported	4
Objectives	3	State specific objectives, including any pre-specified hypotheses	4
**Methods**			
Study design	4	Present key elements of study design early in the paper	5
Setting	5	Describe the setting, locations, and relevant dates, including periods of recruitment, exposure, follow-up, and data collection	5
Participants	6	(*a*) Give the eligibility criteria, and the sources and methods of selection of participants	5
Variables	7	Clearly define all outcomes, exposures, predictors, potential confounders, and effect modifiers. Give diagnostic criteria, if applicable	6
Data sources/ measurement	8*	For each variable of interest, give sources of data and details of methods of assessment (measurement). Describe comparability of assessment methods if there is more than one group	6
Bias	9	Describe any efforts to address potential sources of bias	
Study size	10	Explain how the study size was arrived at	5
Quantitative variables	11	Explain how quantitative variables were handled in the analyses. If applicable, describe which groupings were chosen and why	6
Statistical methods	12	(*a*) Describe all statistical methods, including those used to control for confounding	6
		(*b*) Describe any methods used to examine subgroups and interactions	6-7
		(*c*) Explain how missing data were addressed	Tables 1 and 2
		(*d*) If applicable, describe analytical methods taking account of sampling strategy	
		(*e*) Describe any sensitivity analyses	
**Results**			

For peer review only

Participants	13*	(a) Report numbers of individuals at each stage of study—eg numbers potentially eligible, examined for eligibility, confirmed eligible, included in the study, completing follow-up, and analysed	8
		(b) Give reasons for non-participation at each stage	
		(c) Consider use of a flow diagram	
Descriptive data	14*	(a) Give characteristics of study participants (eg demographic, clinical, social) and information on exposures and potential confounders	8
		(b) Indicate number of participants with missing data for each variable of interest	Tables 1 and 2
Outcome data	15*	Report numbers of outcome events or summary measures	8
Main results	16	(*a*) Give unadjusted estimates and, if applicable, confounder-adjusted estimates and their precision (eg, 95% confidence interval). Make clear which confounders were adjusted for and why they were included	8-12
		(*b*) Report category boundaries when continuous variables were categorized	Tables 1-3
		(*c*) If relevant, consider translating estimates of relative risk into absolute risk for a meaningful time period	
Other analyses	17	Report other analyses done—eg analyses of subgroups and interactions, and sensitivity analyses	12-13
**Discussion**			
Key results	18	Summarise key results with reference to study objectives	14
Limitations	19	Discuss limitations of the study, taking into account sources of potential bias or imprecision. Discuss both direction and magnitude of any potential bias	16
Interpretation	20	Give a cautious overall interpretation of results considering objectives, limitations, multiplicity of analyses, results from similar studies, and other relevant evidence	14-16
Generalisability	21	Discuss the generalisability (external validity) of the study results	14
**Other information**			
Funding	22	Give the source of funding and the role of the funders for the present study and, if applicable, for the original study on which the present article is based	18

*Give information separately for cases and controls in case-control studies and, if applicable, for exposed and unexposed groups in cohort and cross-sectional studies.

Note: An Explanation and Elaboration article discusses each checklist item and gives methodological background and published examples of transparent reporting. The STROBE checklist is best used in conjunction with this article (freely available on the Web sites of PLoS Medicine at http://www.plosmedicine.org/, Annals of Internal Medicine at http://www.annals.org/, and Epidemiology at http://www.epidem.com/). Information on the STROBE Initiative is available at www.strobe-statement.org.

For peer review only

BMJ Open

Implications of the availability and distribution of birthweight on addressing neonatal mortality: Population-based assessment from Bihar state of India

Journal:	*BMJ Open*
Manuscript ID	bmjopen-2022-061934.R2
Article Type:	Original research
Date Submitted by the Author:	25-May-2022
Complete List of Authors:	KUMAR, ANIL; Public Health Foundation of India,<br>George, Sibin; Public Health Foundation of India<br>Akbar, Md.; Public Health Foundation of India<br>Bhattacharya, Debarshi; Bill & Melinda Gates Foundation India<br>Nanda, Priya; Bill and Melinda Gates Foundation India<br>Dandona, Lalit; Public Health Foundation of India<br>Dandona, Rakhi; Public Health Foundation of India; University of Washington
**Primary Subject Heading**:	Epidemiology
Secondary Subject Heading:	Public health
Keywords:	PUBLIC HEALTH, Community child health < PAEDIATRICS, EPIDEMIOLOGY

SCHOLARONE™
Manuscripts

I, the Submitting Author has the right to grant and does grant on behalf of all authors of the Work (as defined in the below author licence), an exclusive licence and/or a non-exclusive licence for contributions from authors who are: i) UK Crown employees; ii) where BMJ has agreed a CC-BY licence shall apply, and/or iii) in accordance with the terms applicable for US Federal Government officers or employees acting as part of their official duties; on a worldwide, perpetual, irrevocable, royalty-free basis to BMJ Publishing Group Ltd ("BMJ") its licensees and where the relevant Journal is co-owned by BMJ to the co-owners of the Journal, to publish the Work in this journal and any other BMJ products and to exploit all rights, as set out in our licence.

The Submitting Author accepts and understands that any supply made under these terms is made by BMJ to the Submitting Author unless you are acting as an employee on behalf of your employer or a postgraduate student of an affiliated institution which is paying any applicable article publishing charge ("APC") for Open Access articles. Where the Submitting Author wishes to make the Work available on an Open Access basis (and intends to pay the relevant APC), the terms of reuse of such Open Access shall be governed by a Creative Commons licence – details of these licences and which Creative Commons licence will apply to this Work are set out in our licence referred to above.

Other than as permitted in any relevant BMJ Author's Self Archiving Policies, I confirm this Work has not been accepted for publication elsewhere, is not being considered for publication elsewhere and does not duplicate material already published. I confirm all authors consent to publication of this Work and authorise the granting of this licence.

For peer review only

25 May 2022

Implications of the availability and distribution of birthweight on addressing neonatal mortality: Population-based assessment from Bihar state of India

G. Anil Kumar[1], Sibin George[1], Md. Akbar[1], Debarshi Bhattacharya[3], Priya Nanda[3], Lalit Dandona[1,2], Rakhi Dandona[1,2]

[1]Public Health Foundation of India, Gurugram, National Capital Region, India

[2]Institute for Health Metrics and Evaluation, University of Washington, Seattle, USA

[3]Bill & Melinda Gates Foundation, India Country Office, New Delhi, India

Corresponding author: Prof. Rakhi Dandona, Public Health Foundation of India, Gurugram, Haryana – 122002, India; rakhi.dandona@phfi.org

Short title: Birthweight in Bihar

For peer review only - http://bmjopen.bmj.com/site/about/guidelines.xhtml

For peer review only

ABSTRACT

Objective: A large proportion of neonatal deaths in India are attributable to low birthweight (LBW). We report population-based distribution and determinants of birthweight in Bihar state, and on the perceptions about birthweight among carers.

Design: A cross-sectional household survey in a state representative sample of 6,007 livebirths born in 2018-2019. Mothers provided detailed interviews on sociodemographic characteristics and birthweight, and their perceptions on LBW (birthweight <2500 g). We report on birthweight availability, LBW prevalence, neonatal mortality rate (NMR) by birthweight, and perceptions of mothers on LBW implications.

Setting: Bihar state, India

Participants: Women with livebirth between October 2018 to September 2019

Results: A total of 5,021 (83.5%) livebirths participated, and 3,939 (78.4%) were weighed at birth. LBW prevalence among those with available birthweight was 18.4% (95% CI 17.1-19.7). Majority (87.5%) of the livebirths born at home were not weighed at birth. LBW prevalence decreased and birthweight >=2,500 g increased significantly with increasing wealth index quartile. NMR was significantly higher in livebirths weighing <1,500 g (11.3%; 95% CI 5.1-23.1) and 1,500-1,999 g (8.0%; 95% CI 4.6-13.6) than those weighing >=2,500 g (1.3%, 95% CI 0.9-1.7. Assuming proportional correspondence of LBW and NMR in livebirths with and without birthweight, the estimated LBW among those without birthweight was 35.5% (95% CI 33.0-38.0) and among all livebirths irrespective of birthweight availability was 23.0% (95% CI 21.9-24.2). Seventy percent of mothers considered LBW to be a sign of sickness, 59.5% perceived it as a risk of developing other illnesses, and 8.6% as having an increased probability of death.

Conclusions: Missing birthweight is substantially compromising the planning of interventions to address LBW at the population-level. Variations of LBW by place of delivery and socio-demographic indicators, and the perceptions of carers about LBW can facilitate appropriate actions to address LBW and the associated neonatal mortality.

For peer review only - http://bmjopen.bmj.com/site/about/guidelines.xhtml

STRENGTHS AND LIMITATIONS OF THE STUDY

- Data on birthweight documented for a representative sample of livebirths including neonatal deaths
- Documentation of birthweight based on recall, which are of reasonable quality based on the global criterion
- Perceptions of care-givers on low birthweight documented in the same population

For peer review only

For peer review only - http://bmjopen.bmj.com/site/about/guidelines.xhtml

INTRODUCTION

Global nutrition targets include a 30% reduction in low birthweight (LBW, weight less than 2500 g) prevalence between 2012 and 2030.(1) LBW is a significant indicator of not only maternal and fetal health predicting mortality and stunting, but also of adult-onset chronic conditions.(2-7) The global LBW prevalence was estimated at 14·6% in 2015,(8, 9) and short gestation for birthweight accounted for an estimated 1·43 million deaths and 139 million DALYs in 2017.(10)

South Asia, with India as its largest component, was estimated to have the highest LBW prevalence for any region in the world in 2015 as per the most recent global update on LBW prevalence which provided country-level estimates.(8, 9) However, LBW prevalence for India was not estimated in that report due to quality concerns with the available data.(8) We have reported LBW prevalence of 21·4% in India in 2017 as part of the Global Burden of Disease (GBD) Study,(11) and that 83% of neonatal deaths could be attributed to LBW in India in 2017.(12) LBW prevalence has shown modest decline over time in India, and it is projected that India is unlikely to meet the LBW national and global nutrition targets.(11) The inadequate availability and quality of birthweight data in India, like many low-income and middle-income countries, is a major hindrance in tracking LBW as a priority.(8, 9, 11)

In this background, we report on a population-based assessment of birthweight in the Indian state of Bihar, which is among the most populous Indian states accounting for a significant burden of neonatal mortality.(12) The LBW prevalence in Bihar was estimated as 23.4% in 2017 in the GBD study.(11) The aim of this report is to provide nuanced data for policy makers and program planners on the availability and distribution of birthweight, and implications of birthweight non-availability on robustness of LBW estimate which is of utmost significance in planning of interventions to reduce LBW in order to address neonatal mortality. Furthermore, we present the perceptions about LBW among the carers which can improve specificity of interventions to address LBW. We use data as is without smoothening or imputation in order to highlight for the policy makers the gaps in the birthweight data that are to be addressed for meaningful action.(8, 11)

For peer review only - http://bmjopen.bmj.com/site/about/guidelines.xhtml

METHODS

The ethics approval for this study was provided by the Institutional Ethics Committee of Public Health Foundation of India (Study number TRC-IEC 418/19). Written informed consent were obtained from all respondents who could read and write, and the information sheet and consent form were explained by the interviewer to those who could not read/write and their thumb impressions were obtained.

For the survey, a state representative sample of 6,000 livebirths was selected using a multistage sampling approach from 37 of the 38 districts of Bihar state, excluding the Lakhisarai district. In the first stage, 70 functioning community/primary health centres (CHC/PHC) were randomly sampled with probability proportional to population size from a total of 445 functioning CHC/PHCs, with each catering to an average of 84 villages. In the next stage, five villages were selected from the catchment area of each of the selected CHC/PHC using the village list available in the Census 2011.(13) To arrive at a cluster size of 300 households, villages with <300 households were combined with an adjacent village, and the large villages were split into equal-sized segments of 300 households using natural boundaries. In total, 350 clusters were sampled using a systematic sampling. Each selected cluster was mapped and all the households (a household was defined as people eating from the same kitchen) were enumerated to identify the livebirths delivered by women aged 15-49 years between October 2018 to September 2019.

The mother/care-giver of each identified livebirth was contacted for a detailed interview irrespective of whether the baby was currently alive. Details on the socio-demography, the pregnancy, delivery, and postnatal care of the eligible livebirth were documented. Specifically, for the analysis reported in this paper, birthweight was recorded from the mother or caregiver of the child based on their recall. We also documented the mother/caretaker's perception of the birthweight for each livebirth (very weak, weak, normal, overweight), and whether the mother/caretaker perceived low birthweight in a baby to be an indication of sickness, and if so why. Furthermore, the possible reasons for LBW in babies, how to prevent LBW, and if the mother/care-

For peer review only - http://bmjopen.bmj.com/site/about/guidelines.xhtml

taker thought if the delivery process was different based on the birthweight were also documented. The questionnaire was developed in English and then translated into Hindi (local language), after which it was back-translated into English to ensure the accurate and relevant meaning and intent of the questions. Pilot testing of the questionnaires was carried out and modifications made as necessary. Data were collected between November 2019 and January 2020 using Open Development Kit by interviewers trained in study procedures. Data entered were scrutinized using the internal consistency checks built in to detect and correct errors using standardised procedures to meet the data quality. To further improve data quality, spot checks were conducted by the supervisors in 10% of the households and back checks were done in 15% of the households. At least three attempts were made to reach out to all the eligible livebirths.

We tested the quality of birthweight data by using the criteria utilised for the report on the global LBW prevalence estimates.(8) Poor quality data was defined as extreme heaping with >55% of all birthweights falling on three values (2500 g, 3000 g, or 3500 g); >10% of births weighed at least 4500 g; or excessive heaping on the tail end of the birthweight distribution with more than 5% of birthweights at 250–500 g and 5500 g. We report on the quality of birthweight data, and for which livebirths the values of 2500 g, 3000 g, or 3500 g are more likely to be reported at the population-level.(8) We assessed the assumption if the data on child not weighted at birth was missing at random in this population using the Little test for missing completely at random.(14)

We categorised birthweight into five categories for analysis - <1,500 g, 1,500-1,999 g, 2,000-2,499 g, <2,500 g (LBW), and 2,500 g or more. We present birthweight prevalence per 100 livebirths for these five categories with 95% confidence intervals, and also for not being weighted at birth, and for birthweight could not be recalled considering all livebirths irrespective of birthweight availability. We then report birthweight prevalence for these five birthweight categories considering only the livebirths for whom birthweight was available. Among these, the prevalence and mean birthweight with standard deviation (SD) is also reported by maternal age, maternal education, wealth index, sex of the baby, length of the pregnancy, place of delivery, and based on livebirth survival. Wealth index

For peer review only - http://bmjopen.bmj.com/site/about/guidelines.xhtml

was estimated using the standard questions and methods used in the National Family Health Survey.(15) Multiple logistic regression was run to investigate the association of LBW among the livebirths with birthweight available with the above variables with all the variables introduced simultaneously in the model. Odds ratio with 95% CI are presented for the regression analysis.

We explored the association of neonatal and post-neonatal mortality with birthweight. Based on the difference in neonatal mortality rates between livebirths for whom birthweight was available versus those for whom birthweight was not available, we also report proportionately adjusted LBW prevalence in those with birthweight available to estimate the LBW prevalence in those with birthweight not available. In addition, a variety of perceptions of the caregivers about LBW are reported. All analysis was performed using STATA 13.1 software (Stata Corp, USA).

Patient and Public Involvement

Patients or the public were not involved in the design, or conduct, or reporting, or dissemination plans of our research.

For peer review only - http://bmjopen.bmj.com/site/about/guidelines.xhtml

RESULTS

We identified 6,007 livebirths representative of the Bihar state between October 2018 to September 2019 from 5,852 women aged 15-49 years in 55,475 households. Detailed interview was available for 5,021 (83.6%) livebirths, majority (98.2%) of whom were singleton births, 2,614 (52.1%) were boys, 2,870 (57.2 %) were born in a public health facility, and 150 (3%) were currently not alive. Of the 5,021 livebirths, 3,939 (78.4%) were weighed at birth but birthweight could not be recalled for 292 (7.4%, 95% CI 6.6-8.3) livebirths.

Quality of birthweight data

Considering the 3,647 livebirths with birthweight available, 52% of all birthweight values fell on 2,500 g, 3,000 g, or 3,500 g; 1.6% livebirths weighed at least 4,500 g; and 0.36% of birthweights were either at 250–500 g or 5,500 g. This indicates data to be of reasonable quality, as the heaping was less than the criteria for poor quality data. Significant variation was seen in the reporting of birthweight values of 2,500 g, 3,000 g, and 3,500 g by maternal age (chi-square, p=0.008), maternal education (chi-square, p<0.001), and place of delivery (chi-square, p=0.028) as shown in Supplementary Figure 1. The data on child not weighted at birth was not missing completely at random (p<0.001).

Distribution of birthweight among all livebirths

Considering all livebirths irrespective of birthweight availability, prevalence of birthweight >=2500 g was 59.3% (95% CI 57.9-60.6), of LBW was 13.3% (95% CI 12.4-14.3), and of livebirths not weighed at birth was 21.5% (95% CI 20.4-22.7) as shown in Supplementary Table 1. Importantly, the prevalence of livebirths not weighed at birth was 87.5 (95% CI 85.4-89.3) in home births as compared with only negligible facility births for whom birthweight was not measured (Supplementary Table 1).

Distribution of birthweight among livebirths with birthweight available

Among livebirths with birthweight available, the mean birthweight was 2,848.2 g with SD of ± 647.2 g (Table 1), and was significantly lower for livebirths born at 6-7 months of gestation (1,710.6 ± 577.4

For peer review only - http://bmjopen.bmj.com/site/about/guidelines.xhtml

g) and for livebirths of younger mothers aged <20 years (2,718.0 ± 642.5 g). Girls, livebirths belonging to lower wealth index quartile, and livebirths who did not survive were significantly more likely to have a lower mean birthweight as compared with boys, those belonging to higher wealth index quartile, and those currently alive, respectively (Table 1).

Table 1. Mean birthweight for livebirths between October 2018 to September 2019 for whom birthweight could be recalled in the Indian state of Bihar.

	Total	Availability of birth weight (% of total)	Mean birthweight (g)
**Overall**	**5,021**	**3,647 (72.6)**	**2,848.2 ± 647.2**
**Maternal age** *†			
15-19 years	529	407 (76.9)	2,718.0 ± 642.5
20-24 years	2,392	1,808 (75.6)	2,836.6 ± 646.3
25-29 years	1,453	1,028 (70.8)	2,911.8 ± 632.8
>=30 years	633	392 (61.9)	2,878.7 ± 662.5
**Maternal education**§†			
No education	1,907	1,172 (61.5)	2,801.0 ± 685.6
Classes 1 to 5	760	544 (71.6)	2,826.0 ± 664.4
More than class 5	2,350	1,928 (82.0)	2,885.4 ± 613.3
**Wealth index quartile**#†			
I	1,255	777 (61.9)	2,781.9 ± 690.1
II	1,255	861 (68.6)	2,800.7 ± 656.0
III	1,255	945 (75.3)	2,879.9 ± 659.2
IV	1,255	1,063 (84.7)	2,907.0 ± 588.0
**Sex**‡			
Boy	2,614	1,939 (74.2)	2,888.7 ± 647.1
Girl	2,407	1,708 (71.0)	2,802.3 ± 644.3
**Gestation period**†			
6-7 months	46	33 (71.7)	1,710.6 ± 577.4
8 months	944	701 (74.3)	2,735.7 ± 631.7
>8 months	4,027	2,910 (72.3)	2,889.7 ± 635.2
**Birth order**†			
1st	1,366	1,110 (81.3)	2,775.2 ± 628.5
2nd	1,369	1,019 (74.4)	2,892.5 ± 653.1
>2nd	2,282	1,515 (66.4)	2,874.8 ± 649.8
**Place of delivery**§†			
Public sector facility	2,870	2,622 (91.4)	2,839.3 ± 625.9
Private sector facility	1,022	890 (87.1)	2,880.7 ± 697.0
Home	1,125	132 (11.7)	2,839.2 ± 679.6
**Current status of livebirth**‡			
Died on day 0 of birth	57	26 (45.6)	2,644.2 ± 1,082.1
Died between day 1-27 of birth	58	40 (69.0)	2,611.3 ± 1,071.3
Died between day 28 and 11 months of age	35	22 (62.9)	2,368.2 ± 771.9
Alive	4,871	3559 (73.1)	2,855.3 ± 634.4

For peer review only - http://bmjopen.bmj.com/site/about/guidelines.xhtml

*Data not available for 14 livebirths
†Chi-square test of significance, p-value <0.001
§Data not available for 4 livebirths
#Data not available for 1 livebirth
‡Chi-square test of significance, p-value =0.001

The prevalence of LBW was 18.4 (95% CI 17.1-19.7), and that of birthweight <1,500 g was 1.5 (95% CI 1.1-1.9), of 1,500-1,999 g was 4.1 (95% CI 3.5-4.8), and of 2,000-2,400 g was 12.8 (95% CI 11.8-13.9) as shown in Table 2. LBW prevalence was 5.6 times higher among the babies who were born with 6-7 months of gestation as compared with those born >8 months of gestation (Table 2 and Figure 1). LBW prevalence decreased and that for birthweight >=2,500 g increased significantly (p<0.001) with increasing wealth index quartile (Table 2 and Figure 1). Using multiple logistic regression (Supplementary Table 2), the most significant odds of having LBW were for livebirths with gestation period of 6-7 months (OR 34.0; 95% CI 11.6-99.6).

Table 2. Prevalence of birthweight by categories among the livebirths who had birthweight available for select characteristics in the Indian state of Bihar for livebirths between October 2018 to September 2019.

	Prevalence per 100 livebirths (95% confidence interval)				
	**Birthweight >=2,500 g**	**Birthweight <2,500 g**	***Birthweight 2,000 - 2,499 g***	***Birthweight 1,500 - 1,999 g***	***Birthweight <1,500 g***
Overall	81.6 (80.3-82.9)	18.4 (17.1-19.7)	*12.8 (11.8-13.9)*	*4.1 (3.5-4.8)*	*1.5 (1.1-1.9)*
**Maternal age***					
15-19 years	73.0 (68.4-77.1)	27.0 (22.9-31.6)	*19.9 (16.3-24.1)*	*6.1 (4.2-8.9)*	*1.0 (0.4-2.6)*
20-24 years	81.5 (79.6-83.2)	18.5 (16.8-20.4)	*13.0 (11.5-14.6)*	*4.0 (3.2-5.1)*	*1.5 (1.0-2.2)*
25-29 years	86.0 (83.7-88.0)	14.0 (12.0-16.3)	*9.2 (7.6-11.2)*	*3.3 (2.4-4.6)*	*1.5 (0.9-2.4)*
>=30 years	80.6 (76.4-84.2)	19.4 (15.8-23.6)	*13.8 (10.7-17.6)*	*4.3 (2.7-6.9)*	*1.3 (0.5-3.0)*
**Maternal education§**					
No education	78.7 (76.2-80.9)	21.3 (19.1-23.8)	*14.0 (12.1-16.1)*	*5.1 (4.0-6.5)*	*2.2 (1.5-3.2)*
Class 1 to 5	79.2 (75.6-82.4)	20.8 (17.6-24.4)	*14.5 (11.8-17.7)*	*5.2 (3.6-7.4)*	*1.1 (0.5-2.4)*
More than class 5	84.2 (82.5-85.8)	15.8 (14.2-17.5)	*11.6 (10.3-13.1)*	*3.2 (2.5-4.1)*	*1.0 (0.6-1.5)*
**Wealth index quartile #**					
I	78.7 (76.2-80.9)	23.0 (20.2-26.1)	*15.3 (13.0-18.0)*	*5.8 (4.4-7.7)*	*1.9 (1.2-3.2)*
II	79.2 (75.6-82.4)	20.8 (18.2-23.6)	*14.1 (11.9-16.5)*	*4.9 (3.6-6.5)*	*1.9 (1.1-3.0)*
III	84.2 (82.5-85.8)	17.7 (15.4-20.2)	*13.2 (11.2-15.5)*	*3.2 (2.2-4.5)*	*1.3 (0.7-2.2)*
IV	78.7 (76.2-80.9)	13.6 (11.7-15.8)	*9.6 (8.0-11.5)*	*3.1 (2.2-4.3)*	*0.9 (0.5-1.7)*
**Sex**					
Boy	84.0 (82.3-85.5)	16.0 (14.5-17.7)	*10.8 (9.5-12.2)*	*4.0 (3.2-4.9)*	*1.3 (0.9-1.9)*
Girl	79.0 (77.0-80.9)	21.0 (19.2-23.0)	*15.1 (13.5-16.9)*	*4.3 (3.4-5.3)*	*1.6 (1.1-2.4)*

For peer review only - http://bmjopen.bmj.com/site/about/guidelines.xhtml

	Prevalence per 100 livebirths (95% confidence interval)				
	**Birthweight >=2,500 g**	**Birthweight <2,500 g**	***Birthweight 2,000 - 2,499 g***	***Birthweight 1,500 - 1,999 g***	***Birthweight <1,500 g***
**Gestation period§**					
6-7 months	12.1 (4.6-28.5)	87.9 (71.5-95.5)	*24.2 (12.5-41.8)*	*36.4 (21.8-54.0)*	*27.3 (14.7-45.0)*
8 months	74.6 (71.3-77.7)	25.4 (22.3-28.8)	*17.7 (15.0-20.7)*	*5.9 (4.3-7.9)*	*1.9 (1.1-3.2)*
>8 months	84.2 (82.8-85.5)	15.8 (14.5-17.2)	*11.5 (10.4-12.7)*	*3.3 (2.7-4.0)*	*1.0 (0.7-1.4)*
**Birth order**					
1st	78.1 (75.6-80.5)	21.9 (19.6-24.4)	*15.4 (13.4-17.7)*	*5.2 (4.1-6.7)*	*1.3 (0.8-2.1)*
2nd	83.4 (81.0-85.6)	16.6 (14.4-19.0)	*12.0 (10.1-14.1)*	*3.2 (2.3-4.5)*	*1.4 (0.8-2.3)*
>2nd	83.2 (81.2-85.0)	16.8 (15.0-18.8)	*11.5 (10.0-13.2)*	*3.8 (3.0-4.9)*	*1.5 (1.0-2.3)*
**Place of delivery§**					
Public sector facility	81.9 (80.4-83.3)	18.1 (16.7-19.6)	*13.2 (12.0-14.6)*	*3.6 (2.9-4.4)*	*1.3 (0.9-1.8)*
Private sector facility	81.7 (79.0-84.1)	18.3 (15.9-21.0)	*11.4 (9.4-13.6)*	*5.4 (4.1-7.1)*	*1.6 (0.9-2.6)*
Home	78.0 (70.1-84.3)	22.0 (15.7-29.9)	*14.4 (9.4-21.5)*	*5.3 (2.5-10.7)*	*2.3 (0.7-6.8)*
**Current status of livebirth**					
Died on day 0 of birth	61.5 (41.7-78.2)	38.5 (21.8-58.3)	*11.5 (3.7-30.8)*	*19.2 (8.1-39.2)*	*7.7 (1.9-26.6)*
Died between day 1-27 of birth	55.0 (39.4-69.7)	45.0 (30.3-60.6)	*17.5 (8.5-32.6)*	*17.5 (8.5-32.6)*	*10.0 (3.8-24.0)*
Died between day 28 and 11 months of age	59.1 (37.7-77.5)	40.9 (22.5-62.3)	*13.6 (4.3-35.5)*	*18.2 (6.8-40.3)*	*9.1 (2.2-30.7)*
Alive	82.2 (80.9-83.4)	17.8 (16.6-19.1)	*12.8 (11.7-13.9)*	*3.8 (3.2-4.4)*	*1.3 (1.0-1.7)*

Of the 670 LBW babies, the parents of 463 (69.1%) livebirths were informed by the health provider that the baby was weak/LBW. This proportion was 87.2% for the 203 livebirths with birthweight of <2,000 g and 94.3% for 53 livebirths with birthweight of <1,500 g. Considering the 190 facility livebirths with birthweight <2,000 g, livebirths at public facility (84%) were significantly less likely to be informed by the health provider of the baby being weak/having LBW as compared with those born in a private sector facility (93.6%; Z test for significance p<0.1).

Mortality and birthweight

A total of 150 (3.0%) livebirths were not currently alive) of whom 114 (76%) had died during the neonatal period (Table 1). The neonatal mortality rate in livebirths weighing <1,500 g (11.3%; 95% CI 5.1-23.1) and 1,500-1,999 g (8.0%; 95% CI 4.6-13.6) was significantly higher than in those weighing >=2,500 g (Table 3). The neonatal mortality rate in livebirths for whom birthweight was not available (3.5; 95% CI 2.6-4.6) was almost twice as high as compared with those for whom birthweight was

For peer review only - http://bmjopen.bmj.com/site/about/guidelines.xhtml

available (1.8%, 95% CI1.4-2.3) as shown in Table 3. Based on this 93% higher neonatal mortality rate among livebirths for whom birthweight was not available, and assuming a direct correspondence between neonatal mortality rate and LBW, we estimated that LBW among livebirths for whom birthweight was not available would be 35.5% (95% CI 33.0-38.0), that is, 93% higher than the 18.4% LBW among livebirths for whom birthweight was available. Based on the proportions of these two groups among all livebirths, we estimated an overall LBW of 23.0% (95% CI 21.9-24.2) among all livebirths.

Table 3. Mortality by birthweight categories among the livebirths born between October 2018 to September 2019 in the Indian state of Bihar. CI denotes confidence interval.

Birthweight	Number of livebirths	Number of neonatal deaths	Neonatal mortality rate, % (95% CI)	Number of deaths in post neonatal period to 11 months of age	Post-neonatal mortality rate to 11 months of age, % (95% CI)
>=2,500 g	2,977	38	1.3 (0.9-1.7)	13	0.4 (0.3-0.8)
<2,500 g	670	28	4.2 (2.9-6.0)	9	1.3 (0.7-2.6)
<1,500 g	*53*	*6*	*11.3 (5.1-23.1)*	*2*	*3.8 (0.9-14.0)*
1,500-1,999 g	*150*	*12*	*8.0 (4.6-13.6)*	*4*	*2.7 (1.0-6.9)*
2,000-2,499 g	*467*	*10*	*2.1 (1.2-3.9)*	*3*	*0.6 (0.2-2.0)*
**Birthweight available**	3,647	66	1.8 (1.4-2.3)	22	0.6 (0.4-0.9)
Not recalled	292	15	5.1 (3.1-8.4)	0	0
Not weighed at birth	1,082	33	3.0 (2.2-4.3)	14	1.3 (0.8-2.2)
**Birthweight not available**	1,374	48	3.5 (2.6-4.6)	14	1.0 (0.6-1.7)
**All livebirths**	**5,021**	**114**	**2.3 (1.9-2.7)**	**96**	**0.7 (0.5-1.0)**

Respondent's perceptions about LBW

Mothers were the predominant respondent in the survey (99.8%). Figure 2 shows the perception of mothers on the birthweight of their livebirth. Overall, 74.7% (3,748) of all mothers of livebirth, 88.1% (2,622) of mothers of livebirths >= 2,500g, and 25.5% (170) of mothers of LBW livebirths perceived their newborns to be of normal weight. Perception of weak or very weak was higher in LBW livebirths (73.3%) as compared with >= 2,500g livebirths (11%). Among the 53 livebirths with birthweight <1,500 g, 36 (67.9%) were perceived to be very weak, 9 (17%) weak and 6 (1.3%) of normal weight by the mother. These perceptions are not mutually exclusive.

A total of 3,527 (70.2%) mothers considered LBW to be a sign of sickness/illness. Among these 3,527 women, 2,988 (84.2%) perceived it as a risk of developing other illnesses, 1,764 (50%)

For peer review only - http://bmjopen.bmj.com/site/about/guidelines.xhtml

considered it a risk for weak growth, and 433 (12.3%) perceived it as having an increased probability of death (not mutually exclusive). Among the 1,350 (26.9%) women who did not consider LBW to be a sickness in a newborn, 1,308 (96.9%) felt that the baby would gain weight after birth and hence there was nothing to worry. Majority (4,570; 91%) of the mothers thought that LBW baby needed extra care; and the extra care practices commonly reported (not mutually exclusive) were oil massage (76.4%), exclusive breastfeeding (74.3%), seeking health care advice (46.6%), and keeping the baby warm (31.2%).

Figure 2 shows the possible reasons of LBW as reported by the mothers (not mutually exclusive). Mother eating less during pregnancy (74.7%), inadequate diet during pregnancy (43.8%), and weak mother (33.2%) were the most cited reasons for LBW baby. Majority of the mothers (94.9%) reported that intake of nutritious diet during pre- and during pregnancy can prevent LBW, followed by full antenatal care check-up (28.3%) and iron and folic acid intake (23.3%). A total of 3,026 (60.8%) mothers perceived the delivery process to be different depending on the birthweight of baby; 2,515 (83.1%) felt that delivery of LBW baby was easier than that of a normal weight baby, 891 (29.4%) thought that C-section was needed less for LBW babies, and 874 (28.9 %) felt that duration of labour was shorter for them (not mutually exclusive).

For peer review only

For peer review only - http://bmjopen.bmj.com/site/about/guidelines.xhtml

DISCUSSION

We present the estimates for birthweight prevalence across various categories in the Indian state of Bihar, including LBW prevalence which is essential for tracking progress towards the national and global nutrition targets. These estimates are presented in two ways – including and excluding livebirths based on birthweight availability – to highlight the need for improved birthweight availability to arrive at robust understanding of LBW prevalence for appropriate action both within the health system and the community. Socio-demographic distribution of livebirths for whom birthweight was not available can facilitate formulating specific actions in these populations to improve birthweight availability. Notably, the perceptions of mothers regarding reasons for LBW and its implications can provide a framework for developing relevant actions to improve care of LBW babies and possible actions to reduce LBW prevalence.

Birthweight was missing for 1 out of 4 livebirths in this population. Extrapolating our findings to the estimated 2.5 million livebirths in 2019 in Bihar, 543,000 livebirths were not weighted at birth and recall was not available for 146,600. Though home births accounted for only 22% of all livebirths in this population, these accounted for majority of the livebirths who were not weighted at birth. Therefore, until facility births can be increased further in the long-term that could result in increased birthweight measurement, tracking LBW as a priority target is not possible unless urgent targeted efforts are made in the short-term to engage with the health providers who assist with home births to improve birthweight availability.

Overall, birthweight data in our study was of reasonable quality as per the criteria used in the recent report on global estimation of LBW prevalence.(8) Unlike other reports,(8, 9) we did not smoothen the data for heaping, but have presented data as is to enhance understanding of where heaping was more likely to be reported to facilitate development of targeted approach in addressing this heaping. For the policy makers and program planners it is imperative to note where most action is needed to improve robustness of birthweight estimates. One of the assumptions made in the recent global report on LBW prevalence was that missing birthweights are missing at random and

For peer review only - http://bmjopen.bmj.com/site/about/guidelines.xhtml

that the true distribution of birthweights in a population can be approximated by a mixture of two normal distributions.(8) Our data has highlight that birthweight is not missing at random but in specific sub-groups, and this may be need to be taken into account in assumptions for global estimates.

The LBW prevalence estimated was 18.4% considering only livebirths with birthweight available, and 23% in all livebirths by proportionately adjusting for those who did not birthweight available based on their higher neonatal mortality rate. Even though the adjustment made for neonatal mortality is fairly simplistic, the extent of variation in LBW prevalence with this adjustment conveys the enormous implications of non-availability of birthweight for the planning of interventions and to appropriately allocate resources to address LBW at the population-level. Those without birthweight accounted for one-third of all neonatal deaths, and birthweight availability was less than half for the livebirths who had died on day 0. Importantly, the LBW prevalence was estimated to be almost twice among livebirths for whom birthweight was not available versus those for whom birthweight was available. This finding is of significance as we have previously reported that 50% of all neonatal mortality in the state to be in 0-2 days of birth, with 35% of them not weighted at birth.(16) Though the current study included only livebirths, our previous work in Bihar has also documented birthweight non-availability at 85% for stillbirths.(17) One of the proposed newborn quality of care indicator at health-facility level in low- and middle-income setting is facility neonatal mortality rate disaggregated by birth weight.(18) With majority of births now in the facilities, urgent and sustained effort is needed to track this quality indicator on a routine basis, which is currently not tracked in the Indian health information system. Interestingly, the Civil Registration System captures the birthweight for all births but that data is not available in public domain to comment on availability and quality of that data.(19) As LBW and short gestation are the predominant risk factors for neonatal mortality in India and in Bihar,(12) ensuring birthweight is measured for all livebirths irrespective of survival at birth is extremely important. Understanding the health providers perspectives on the need of birthweight measurement and quality is an

For peer review only - http://bmjopen.bmj.com/site/about/guidelines.xhtml

understudied issue,(20) and effort to improve this understanding is needed urgently to improve birthweight documentation.

A significant focus of neonatal health programs is on caring for the small and sick newborns, and communication with the carer/family is an integral part for their meaningful participation.(21) Seven in 10 carers of LBW babies were informed by the health provider that the baby was weak/LBW, and this proportion increased with decreasing birthweight. Some additional effort is needed in the public sector facilities as the families of babies born there were less likely to be informed than those in the private sector and informing birthweight and its implications by them to the family. Importantly, 70% of the mothers interviewed considered LBW to be a sign of sickness/illness, and such level of awareness could be translated not only into demand for availability of birthweight in the community, but also to increase uptake of relevant interventions for LBW babies.(22-27)

The finding of decrease in prevalence of LBW and increase in birthweight >=2,500 g with increasing wealth index quartile is not surprising, given that maternal undernutrition is associated with poor maternal-fetal outcomes including LBW.(2-6, 28) Despite decades of efforts in India to tackle malnutrition, it was the predominant risk factor for under-5 deaths in every state of India in 2017, accounting for 68·2% of the total under-5 deaths.(11) Globally, India has the highest prevalence of BMI lower than 16 in women, with less prevalence in women belonging to higher wealth index.(29) Evidence from Bangladesh suggests that low levels of women's empowerment are associated with maternal undernutrition as well as with delivering LBW babies, and empowerment is lower in women of lower wealth index.(28) What is noteworthy is that majority of the women in our study were well aware of the link between maternal nutrition and LBW, highlighting that facilitators are needed to translate this awareness into action to improve maternal nutrition, which can be achieved by bringing convergence of variety of nutrition-related activities of various government ministries and stakeholders for maternal health across the life cycle.(11, 30-34)

For peer review only - http://bmjopen.bmj.com/site/about/guidelines.xhtml

Documentation of birthweight based on recall in this study could be considered a limitation, however, these data were of reasonable quality using the global criterion.(8) The strengths of our study include an attempt to estimate LBW for all livebirths at the population level, and inclusion of carer perspectives in addition to birthweight availability that can facilitate actionable interventions or further implementation research to improve tracking of LBW, which is a priority global health indicator.

For peer review only

CONCLUSION

Significant efforts are needed in India beyond what is has been done so far to increase the availability and quality of birthweight in order to improve robustness of LBW estimates, which can help planning of appropriate interventions and investments to address this important risk factor of neonatal mortality. Without robust birthweight estimates, India may not able to address neonatal mortality effectively to meet the Sustainable Development Goal by 2030.

For peer review only

For peer review only - http://bmjopen.bmj.com/site/about/guidelines.xhtml

ACKNOWLEDGEMENTS

The authors acknowledge the contributions of Moutushi Majumder and Kaavya Singh from Public Health Foundation of India, and Asif Iqbal and Vipul Singhal from the Oxford Policy Management, India for data collection and data management.

AUTHORS' CONTRIBUTIONS

RD and GAK had full access to data in the study, take full responsibility for the integrity of data and accuracy of the data analysis, and had final responsibility for the decision to submit for publication; RD, GAK and LD conceptualized the study; RD guided the data analysis and drafted the manuscript; SG performed data analysis; MA guided data collection; MA, DB, PN and LD contributed to data analysis and interpretation; all authors approved the final manuscript.

FUNDING

This work was supported by Bill & Melinda Gates Foundation grant number INV-007989.

COMPETING INTEREST

PN and DB are employees of Bill & Melinda Gates Foundation. Other authors declare no completing interests.

DATA AVAILABILITY STATEMENT

All the data of the current study is available with the corresponding author, can be made available on request.

ETHICS APPROVAL

The ethics approval for this study was provided by the Institutional Ethics Committee of Public Health Foundation of India (Study number TRC-IEC 418/19).

For peer review only

REFERENCES

1. World Health Organization. Global nutrition monitoring framework: operational guidance for tracking progress in meeting targets for 2025. Geneva: WHO, 2017.
2. Katz J, Lee AC, Kozuki N, Lawn JE, Cousens S, Blencowe H, et al. Mortality risk in preterm and small-for-gestational-age infants in low-income and middle-income countries: a pooled country analysis. Lancet. 2013;382(9890):417-25.
3. Loret de Mola C, de França GV, Quevedo Lde A, Horta BL. Low birth weight, preterm birth and small for gestational age association with adult depression: systematic review and meta-analysis. Br J Psychiatry. 2014;205(5):340-7.
4. Oudgenoeg-Paz O, Mulder H, Jongmans MJ, van der Ham IJM, Van der Stigchel S. The link between motor and cognitive development in children born preterm and/or with low birth weight: A review of current evidence. Neurosci Biobehav Rev. 2017;80:382-93.
5. Shinzawa M, Tanaka S, Tokumasu H, Takada D, Tsukamoto T, Yanagita M, et al. Association of Low Birth Weight With Childhood Proteinuria at Age 3 Years: A Population-Based Retrospective Cohort Study. Am J Kidney Dis. 2019;74(1):141-3.
6. Victora CG, Adair L, Fall C, Hallal PC, Martorell R, Richter L, et al. Maternal and child undernutrition: consequences for adult health and human capital. Lancet. 2008;371(9609):340-57.
7. Wojcik W, Lee W, Colman I, Hardy R, Hotopf M. Foetal origins of depression? A systematic review and meta-analysis of low birth weight and later depression. Psychol Med. 2013;43(1):1-12.
8. Blencowe H, Krasevec J, de Onis M, Black RE, An X, Stevens GA, et al. National, regional, and worldwide estimates of low birthweight in 2015, with trends from 2000: a systematic analysis. Lancet Glob Health. 2019;7(7):e849-e60.
9. United Nations Children's Fund (UNICEF) and World Health Organization. UNICEF-WHO Low birthweight estimates: Levels and trends 2000–2015. Geneva: UNICEF and WHO, 2019.
10. GBD 2017 Risk Factor Collaborators. Global, regional, and national comparative risk assessment of 84 behavioural, environmental and occupational, and metabolic risks or clusters of risks for 195 countries and territories, 1990-2017: a systematic analysis for the Global Burden of Disease Study 2017. Lancet. 2018;392(10159):1923-94.
11. India State-Level Disease Burden Initiative Malnutrition Collaborators. The burden of child and maternal malnutrition and trends in its indicators in the states of India: the Global Burden of Disease Study 1990-2017. Lancet Child Adolesc Health. 2019;3(12):855-70.
12. India State-Level Disease Burden Initiative Child Mortality Collaborators. Subnational mapping of under-5 and neonatal mortality trends in India: the Global Burden of Disease Study 2000-17. Lancet. 2020;395(10237):1640-58.
13. Office of the Registrar General & Census Commissioner of India GoI, . C.D. Blockwise Primary Census Abstract Data (PCA) - Bihar: RGI; [cited 2021 23 June]. Available from: http://censusindia.gov.in/pca/cdb_pca_census/Houselisting-housing-BR.html.
14. Li C. Little's test of missing completely at random. The Stata Journal. 2013;13(4):795-809.
15. International Institute for Population Sciences. About NFHS: IIPS; [cited 2021 20 August]. Available from: http://rchiips.org/nfhs/about.shtml.
16. Dandona R, Kumar GA, Bhattacharya D, Akbar M, Atmavilas Y, Nanda P, et al. Distinct mortality patterns at 0-2 days versus the remaining neonatal period: results from population-based assessment in the Indian state of Bihar. BMC Med. 2019;17(1):140.
17. Dandona R, Kumar GA, Akbar M, Bhattacharya D, Nanda P, Dandona L. Deferred and referred deliveries contribute to stillbirths in the Indian state of Bihar: results from a population-based survey of all births. BMC Med. 2019;17(1):28.

For peer review only - http://bmjopen.bmj.com/site/about/guidelines.xhtml

18. Madaj B, Smith H, Mathai M, Roos N, van den Broek N. Developing global indicators for quality of maternal and newborn care: a feasibility assessment. Bull World Health Organ. 2017;95(6):445-52I.
19. Office of the Registrar General & Census Commissioner of India GoI. Birth Report: RGI; [cited 2021 23 August]. Available from: https://censusindia.gov.in/2011-Documents/CRS_Forms/CRS%20Forms.pdf.
20. Gladstone ME, Salim N, Ogillo K, Shamba D, Gore-Langton GR, Day LT, et al. Birthweight measurement processes and perceived value: qualitative research in one EN-BIRTH study hospital in Tanzania. BMC Pregnancy Childbirth. 2021;21(Suppl 1):232.
21. World Health Organization. Standards for improving the quality of care for small and sick newborns in health facilities. Geneva: WHO, 2020.
22. Koenraads M, Phuka J, Maleta K, Theobald S, Gladstone M. Understanding the challenges to caring for low birthweight babies in rural southern Malawi: a qualitative study exploring caregiver and health worker perceptions and experiences. BMJ Glob Health. 2017;2(3):e000301.
23. Lydon M, Longwe M, Likomwa D, Lwesha V, Chimtembo L, Donohue P, et al. Starting the conversation: community perspectives on preterm birth and kangaroo mother care in southern Malawi. J Glob Health. 2018;8(1):010703.
24. Nisha MK, Raynes-Greenow C, Rahman A, Alam A. Perceptions and practices related to birthweight in rural Bangladesh: Implications for neonatal health programs in low- and middle-income settings. PLoS One. 2019;14(12):e0221691.
25. Chan G, Bergelson I, Smith ER, Skotnes T, Wall S. Barriers and enablers of kangaroo mother care implementation from a health systems perspective: a systematic review. Health Policy Plan. 2017;32(10):1466-75.
26. Mazumder S, Upadhyay RP, Hill Z, Taneja S, Dube B, Kaur J, et al. Kangaroo mother care: using formative research to design an acceptable community intervention. BMC Public Health. 2018;18(1):307.
27. Smith ER, Bergelson I, Constantian S, Valsangkar B, Chan GJ. Barriers and enablers of health system adoption of kangaroo mother care: a systematic review of caregiver perspectives. BMC Pediatr. 2017;17(1):35.
28. Kabir A, Rashid MM, Hossain K, Khan A, Sikder SS, Gidding HF. Women's empowerment is associated with maternal nutrition and low birth weight: evidence from Bangladesh Demographic Health Survey. BMC Womens Health. 2020;20(1):93.
29. Razak F, Corsi DJ, Slutsky AS, Kurpad A, Berkman L, Laupacis A, et al. Prevalence of Body Mass Index Lower Than 16 Among Women in Low- and Middle-Income Countries. JAMA. 2015;314(20):2164-71.
30. Kim SS, Avula R, Ved R, Kohli N, Singh K, van den Bold M, et al. Understanding the role of intersectoral convergence in the delivery of essential maternal and child nutrition interventions in Odisha, India: a qualitative study. BMC Public Health. 2017;17(1):161.
31. McKerricher L, Petrucka P. Maternal nutritional supplement delivery in developing countries: a scoping review. BMC Nutr. 2019;5:8.
32. Noznesky EA, Ramakrishnan U, Martorell R. A situation analysis of public health interventions, barriers, and opportunities for improving maternal nutrition in Bihar, India. Food Nutr Bull. 2012;33(2 Suppl):S93-103.
33. Young MF, Bootwala A, Kachwaha S, Avula R, Ghosh S, Sharma PK, et al. Understanding Implementation and Improving Nutrition Interventions: Barriers and Facilitators of Using Data Strategically to Inform the Implementation of Maternal Nutrition in Uttar Pradesh, India. Curr Dev Nutr. 2021;5(6):nzab081.
34. Ministry of Women and Child Development. Government of India. Administrative guidelines for implementation of National Nutrition Mission.: Ministry of Women and Child Development; 2018 [cited 2021 23 August]. Available from: https://icds-wcd.nic.in/nnm/NNM-Web-

For peer review only - http://bmjopen.bmj.com/site/about/guidelines.xhtml

Contents/UPPER-MENU/AdministrativeApproval-Guidelines/Administrative_Guidelines_NNM-26022018.pdf.

For peer review only

FIGURES

Figure 1. Distribution of birthweight by the gestation period and wealth index quartile for livebirths between October 2018 to September 2019 for whom birthweight was available in the Indian state of Bihar.

Figure 2. Factors perceived as responsible for low birthweight in babies among the mothers of livebirths between October 2018 to September 2019 in the Indian state of Bihar.

SUPPLEMENTARY MATERIAL

Supplementary Figure 1. Distribution of birthweight values of 2500 g, 3,000 g, and 3,500 g by select variables among the livebirths born between October 2018 to September 2019 for whom birthweight was available in the Indian state of Bihar.

Supplementary Table 1. Prevalence of birthweight by categories, of no recall, and of child not being weighted at birth for select characteristics in the Indian state of Bihar for livebirths between October 2018 to September 2019.

Supplementary Table 2. Association of low birthweight (LBW) among babies with birthweight available with select variables using multiple logistic regression for livebirths between October 2018 to September 2019 in the Indian state of Bihar.

For peer review only

Figure 1. Distribution of birthweight by the gestation period and wealth index quartile for livebirths between October 2018 to September 2019 for whom birthweight was available in the Indian state of Bihar.

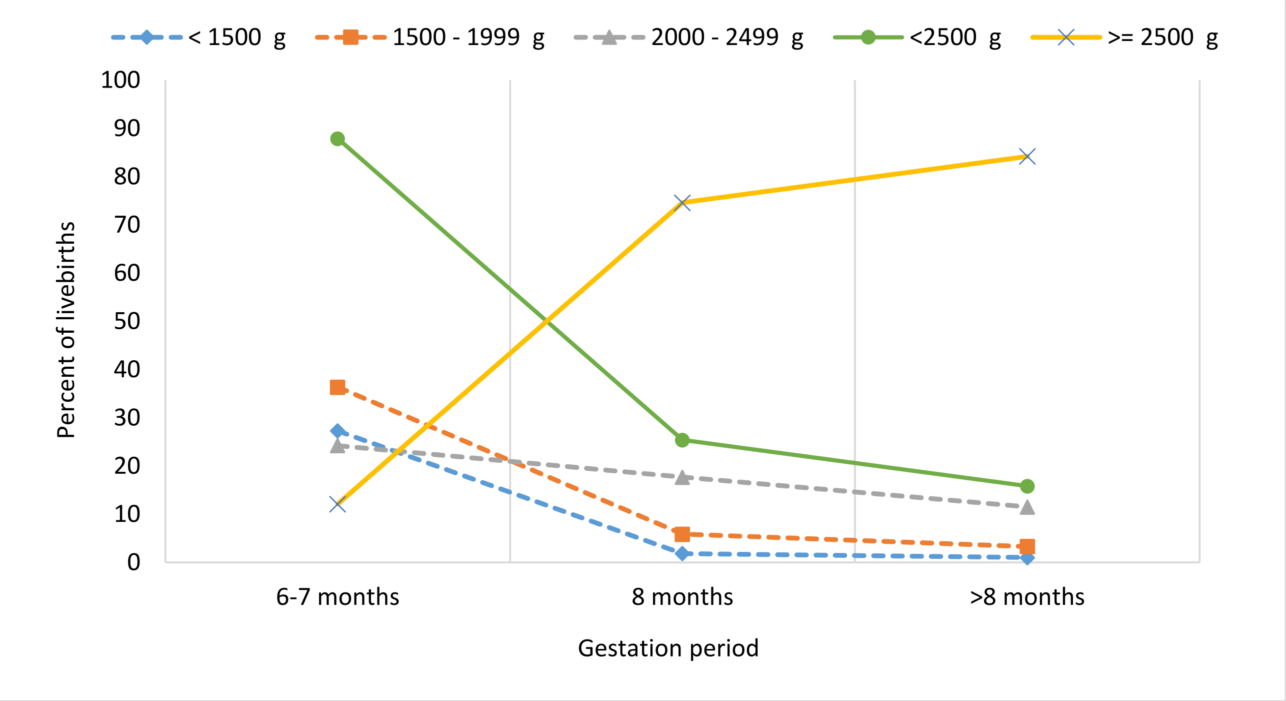

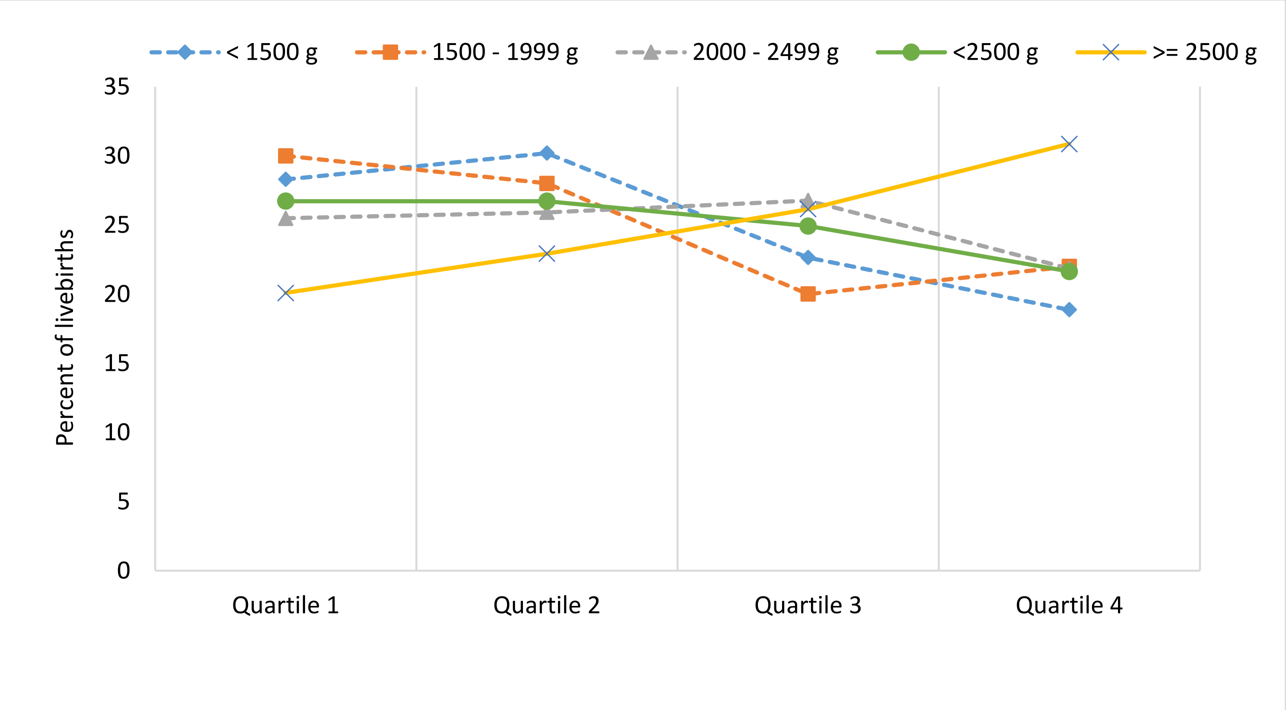

For peer review only - http://bmjopen.bmj.com/site/about/guidelines.xhtml

Figure 2. Factors perceived as responsible for low birthweight in babies among the mothers of livebirths between October 2018 to September 2019 in the Indian state of Bihar. These perceptions are not mutually exclusive.

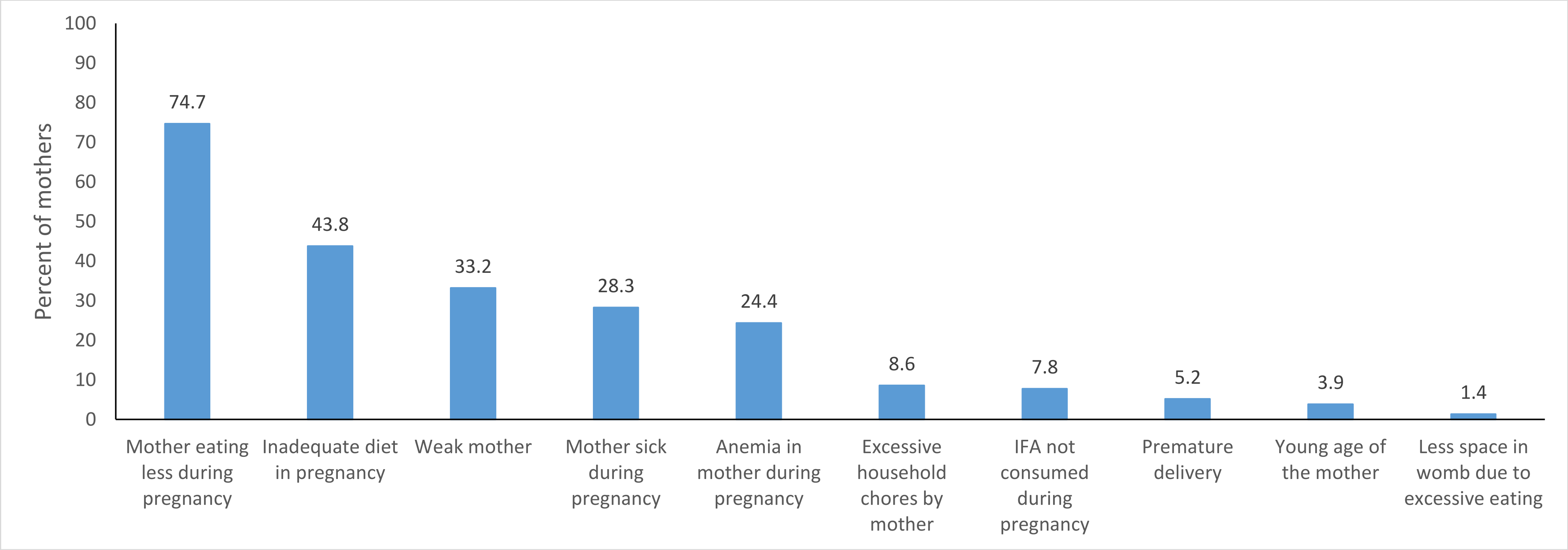

Supplementary Figure 1. Distribution of birthweight values of 2500 g, 3,000 g, and 3,500 g by select variables among the livebirths born between October 2018 to September 2019 for whom birthweight was available in the Indian state of Bihar.

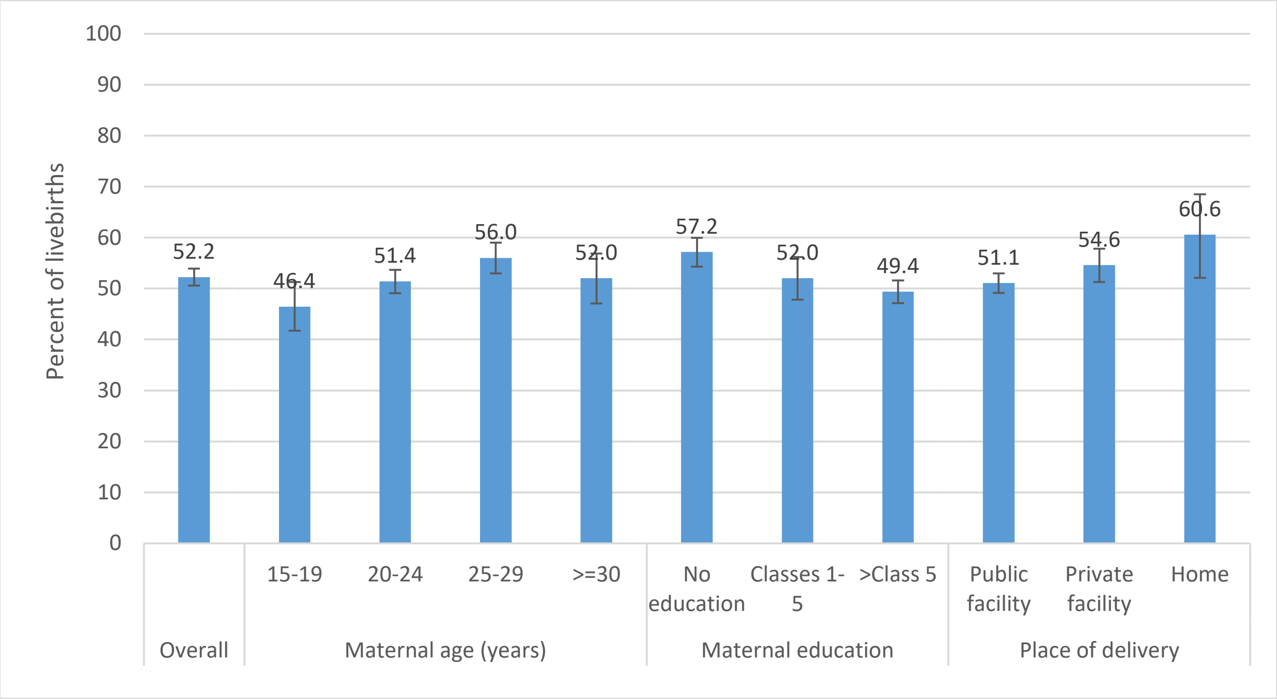

For peer review only

Supplementary Table 1. Prevalence of birthweight by categories, of no recall, and of child not being weighted at birth for select characteristics in the Indian state of Bihar for livebirths between October 2018 to September 2019.

	Prevalence per 100 livebirths (95% confidence interval)						
	**Birthweight >=2,500 g**	**Birthweight <2,500 g**	***Birthweight 2,000 - 2,499 g***	***Birthweight 1,500 - 1,999 g***	***Birthweight <1,500 g***	**Mother could not recall birthweight**	**Child not weighted at birth**
Overall	59.3 (57.9-60.6)	13.3 (12.4-14.3)	*9.3 (8.5-10.1)*	*3.0 (2.6-3.5)*	*1.1 (0.8-1.4)*	5.8 (5.2-6.5)	21.5 (20.4-22.7)
**Maternal age**[*]							
15-19 years	56.1 (51.9-60.3)	20.8 (17.5-24.5)	*15.3 (12.5-18.6)*	*4.7 (3.2-6.9)*	*0.8 (0.3-2.0)*	6.0 (4.3-8.4)	17.0 (14.0-20.5)
20-24 years	61.6 (59.6-63.5)	14.0 (12.7-15.5)	*9.8 (8.7-11.1)*	*3.1 (2.4-3.8)*	*1.1 (0.8-1.6)*	5.1 (4.3-6.1)	19.3 (17.7-20.9)
25-29 years	60.8 (58.3-63.3)	9.9 (8.5-11.6)	*6.5 (5.4-7.9)*	*2.3 (1.7-3.3)*	*1.0 (0.6-1.7)*	6.5 (5.4-7.9)	22.7 (20.6-24.9)
>=30 years	49.9 (46.0-53.8)	12.0 (9.7-14.8)	*8.5 (6.6-11.0)*	*2.7 (1.7-4.3)*	*0.8 (0.3-1.9)*	6.5 (4.8-8.7)	31.6 (28.1-35.3)
**Maternal education**[§]							
No education	48.3 (46.1-50.6)	13.1 (11.7-14.7)	*8.6 (7.4-9.9)*	*3.1 (2.5-4.0)*	*1.4 (0.9-2.0)*	8.0 (6.8-9.3)	30.6 (28.5-32.7)
Class 1 to 5	56.7 (53.2-60.2)	14.9 (12.5-17.6)	*10.4 (8.4-12.8)*	*3.7 (2.6-5.3)*	*0.8 (0.4-1.7)*	5.7 (4.2-7.5)	22.8 (19.9-25.9)
More than class 5	69.1 (67.2-70.9)	12.9 (11.6-14.4)	*9.5 (8.4-10.8)*	*2.6 (2.0-3.3)*	*0.8 (0.5-1.3)*	4.1 (3.4-5.0)	13.8 (12.5-15.3)
**Wealth index quartile** [#]							
I	47.6 (44.9-50.4)	14.3 (12.4-16.3)	*9.5 (8.0-11.2)*	*3.6 (2.7-4.8)*	*1.2 (0.7-2.0)*	6.1 (4.9-7.6)	32.0 (29.4-34.6)
II	54.3 (51.6-57.1)	14.3 (12.4-16.3)	*9.6 (8.1-11.4)*	*3.3 (2.5-4.5)*	*1.3 (0.8-2.1)*	7.3 (5.9-8.8)	24.1 (21.9-26.6)
III	62.0 (59.3-64.6)	13.3 (11.5-15.3)	*10.0 (8.4-11.7)*	*2.4 (1.7-3.4)*	*1.0 (0.5-1.7)*	7.0 (5.7-8.6)	17.7 (15.7-19.9)
IV	73.1 (70.6-75.5)	11.6 (9.9-13.4)	*8.1 (6.7-9.8)*	*2.6 (1.9-3.7)*	*0.8 (0.4-1.5)*	2.9 (2.1-4.0)	12.4 (10.7-14.4)
**Sex**							
Boy	62.3 (60.4-64.1)	11.9 (10.7-13.2)	*8.0 (7.0-9.1)*	*2.9 (2.4-3.7)*	*1.0 (0.6-1.4)*	5.6 (4.8-6.5)	20.2 (18.7-21.8)
Girl	56.0 (54.1-58.0)	14.9 (13.5-16.4)	*10.7 (9.5-12.0)*	*3.0 (2.4-3.8)*	*1.2 (0.8-1.7)*	6.1 (5.2-7.1)	23.0 (21.3-24.7)
**Gestation period§**							
6-7 months	8.7 (3.3-21.2)	63.0 (48.2-75.8)	*17.4 (8.9-31.3)*	*26.1 (15.4-40.7)*	*19.6 (10.4-33.7)*	8.7 (3.3-21.2)	19.6 (10.4-33.7)
8 months	55.4 (52.2-58.5)	18.9 (16.5-21.5)	*13.1 (11.1-15.4)*	*4.3 (3.2-5.8)*	*1.4 (0.8-2.4)*	5.4 (4.1-7.0)	20.3 (17.9-23.0)
>8 months	60.8 (59.3-62.3)	11.4 (10.5-12.4)	8.3 (7.5-9.2)	2.4 (2.0-2.9)	0.7 (0.5-1.0)	5.9 (5.2-6.7)	21.9 (20.6-23.2)
**Birth order**							
1st	63.5 (60.9-66.0)	17.8 (15.8-19.9)	12.5 (10.9-14.4)	4.2 (3.3-5.5)	1.0 (0.6-1.7)	5.1 (4.0-6.3)	13.7 (12.0-15.6)
2nd	62.1 (59.5-64.6)	12.3 (10.7-14.2)	8.9 (7.5-10.5)	2.4 (1.7-3.4)	1.0 (0.6-1.7)	5.8 (4.7-7.1)	19.8 (17.8-22.0)
>2nd	55.2 (53.2-57.2)	11.2 (9.9-12.5)	7.6 (6.6-8.8)	2.5 (2.0-3.3)	1.0 (0.7-1.5)	6.3 (5.4-7.4)	27.3 (25.5-29.2)

	Prevalence per 100 livebirths (95% confidence interval)						
	**Birthweight >=2,500 g**	**Birthweight <2,500 g**	***Birthweight 2,000 - 2,499 g***	***Birthweight 1,500 - 1,999 g***	***Birthweight <1,500 g***	**Mother could not recall birthweight**	**Child not weighted at birth**
**Place of delivery§**							
Public sector facility	74.8 (73.2-76.4)	16.6 (15.2-18.0)	*12.1 (10.9-13.3)*	*3.3 (2.7-4.0)*	*1.2 (0.8-1.7)*	7.3 (6.4-8.3)	1.3 (1.0-1.8)
Private sector facility	71.1 (68.3-73.8)	15.9 (13.8-18.3)	*9.9 (8.2-11.9)*	*4.7 (3.6-6.2)*	*1.4 (0.8-2.3)*	7.1 (5.7-8.9)	5.8 (4.5-7.4)
Home	9.2 (7.6-11.0)	2.6 (1.8-3.7)	*1.7 (1.1-2.6)*	*0.6 (0.3-1.3)*	*0.3 (0.1-0.8)*	0.8 (0.4-1.5)	87.5 (85.4-89.3)
**Current status of livebirth**							
Died on day 0 of birth	28.1 (17.9-41.1)	17.5 (9.7-29.8)	*5.3 (1.7-15.2)*	*8.8 (3.7-19.5)*	*3.5 (0.9-13.1)*	14.0 (7.1-25.8)	40.4 (28.4-53.6)
Died between day 1-27 of birth	37.9 (26.4-51.1)	31.0 (20.4-44.1)	*12.1 (5.8-23.8)*	*12.1 (5.8-23.3)*	*6.9 (2.6-17.1)*	*12.1 (5.8-23.3)*	*19.0 (10.8-31.2)*
Died between day 28 and 11 months of age	37.1 (22.8-54.2)	25.7 (13.8-42.8)	*8.6 (2.7-23.8)*	*11.4 (4.3-27.1)*	*5.7 (1.4-20.5)*	*0*	*37.1 (22.8-54.2)*
Alive	60.1 (58.7-61.4)	13.0 (12.1-14.0)	*9.3 (8.5-10.2)*	*2.8 (2.3-3.2)*	*0.9 (0.7-1.2)*	5.7 (5.1-6.4)	21.2 (20.1-22.4)

*Data not available for 14 livebirths
§Data not available for 4 livebirths
Data not available for 1 livebirth

For peer review only

Supplementary Table 2. Association of low birth weight (LBW) among babies with birth weight available with select variables using multiple logistic regression for livebirths between October 2018 to September 2019 in the Indian state of Bihar.

	<2,500 g birthweight (LBW)		
**Variables**	**Total N=3,647 (% of total)**	**% of livebirths with LBW**	**Odds ratio for having LBW (95% confidence interval)**
**Maternal age***			
15-19 years	407 (11.2)	110 (27.0)	1.00
20-24 years	1808 (49.7)	335 (18.5)	0.8 (0.6-1.0)
25-29 years	1028 (28.3)	144 (14.0)	0.5 (0.4-0.8)
>=30 years	392 (10.8)	76 (19.4)	0.7 (0.5-1.1)
**Maternal education**[#†]			
No education	1172 (32.2)	250 (21.3)	1.4 (1.1-1.8)
Classes 1 to 5	544 (14.9)	113 (20.8)	1.3 (1.0-1.7)
More than class 5	1928 (52.9)	304 (15.8)	1.00
**Wealth index quartile**[‡]			
I	777 (21.3)	179 (23.0)	1.8 (1.3-2.3)
II	861 (23.6)	179 (20.8)	1.6 (1.2-2.1)
III	945 (25.9)	167 (17.7)	1.3 (1.0-1.7)
IV	1063 (29.2)	145 (13.6)	1.00
**Sex**			
Boy	1939 (53.2)	311 (16.0)	1.00
Girl	1708 (46.8)	359 (21.0)	1.4 (1.2-1.6)
**Gestation period**[#]			
6-7 months	33 (0.9)	29 (87.9)	34.0 (11.6-99.6)
8 months	701 (19.2)	178 (25.4)	1.8 (1.5-2.3)
>8 months	2910 (79.9)	460 (15.8)	1.00
**Birth order**[#]			
1st	1110 (30.5)	243 (21.9)	1.00
2nd	1019 (28.0)	169 (16.6)	0.8 (0.6-1.0)
>2nd	1515 (41.6)	255 (16.8)	0.8 (0.6-1.0)
**Place of delivery**[#§]			
Public sector facility	2622 (72.0)	475 (18.1)	1.00
Private sector facility	890 (24.4)	163 (18.3)	1.0 (0.8-1.3)
Home/on route	132 (3.6)	29 (22.0)	1.2 (0.8-1.8)
**Current status of livebirth**			
Died on day 0 of birth	26 (0.7)	10 (38.5)	1.9 (0.8-4.5)
Died between day 1-27 of birth	40 (1.1)	18 (45.0)	1.8 (0.9-3.8)
Died between day 28 and 11 months of age	22 (0.6)	9 (40.9)	2.6 (1.1-6.4)
Alive	3559 (97.6)	633 (17.8)	1.00

For peer review only

*Data not available for 12 livebirths
†p-value <0.001, chi-square test of significance
§p-value= 0.536, chi-square test of significance
#Data not available for 3 livebirths
‡Data not available for 1 livebirth

For peer review only

STROBE 2007 (v4) Statement—Checklist of items that should be included in reports of *cross-sectional studies*

Section/Topic	Item #	Recommendation	Reported on page #
**Title and abstract**	1	(*a*) Indicate the study's design with a commonly used term in the title or the abstract	2
		(*b*) Provide in the abstract an informative and balanced summary of what was done and what was found	2
**Introduction**			
Background/rationale	2	Explain the scientific background and rationale for the investigation being reported	4
Objectives	3	State specific objectives, including any pre-specified hypotheses	4
**Methods**			
Study design	4	Present key elements of study design early in the paper	5
Setting	5	Describe the setting, locations, and relevant dates, including periods of recruitment, exposure, follow-up, and data collection	5
Participants	6	(*a*) Give the eligibility criteria, and the sources and methods of selection of participants	5
Variables	7	Clearly define all outcomes, exposures, predictors, potential confounders, and effect modifiers. Give diagnostic criteria, if applicable	6
Data sources/ measurement	8*	For each variable of interest, give sources of data and details of methods of assessment (measurement). Describe comparability of assessment methods if there is more than one group	6
Bias	9	Describe any efforts to address potential sources of bias	
Study size	10	Explain how the study size was arrived at	5
Quantitative variables	11	Explain how quantitative variables were handled in the analyses. If applicable, describe which groupings were chosen and why	6
Statistical methods	12	(*a*) Describe all statistical methods, including those used to control for confounding	6
		(*b*) Describe any methods used to examine subgroups and interactions	6-7
		(*c*) Explain how missing data were addressed	Tables 1 and 2
		(*d*) If applicable, describe analytical methods taking account of sampling strategy	
		(*e*) Describe any sensitivity analyses	
**Results**			

For peer review only

Participants	13*	(a) Report numbers of individuals at each stage of study—eg numbers potentially eligible, examined for eligibility, confirmed eligible, included in the study, completing follow-up, and analysed	8
		(b) Give reasons for non-participation at each stage	
		(c) Consider use of a flow diagram	
Descriptive data	14*	(a) Give characteristics of study participants (eg demographic, clinical, social) and information on exposures and potential confounders	8
		(b) Indicate number of participants with missing data for each variable of interest	Tables 1 and 2
Outcome data	15*	Report numbers of outcome events or summary measures	8
Main results	16	(*a*) Give unadjusted estimates and, if applicable, confounder-adjusted estimates and their precision (eg, 95% confidence interval). Make clear which confounders were adjusted for and why they were included	8-12
		(*b*) Report category boundaries when continuous variables were categorized	Tables 1-3
		(*c*) If relevant, consider translating estimates of relative risk into absolute risk for a meaningful time period	
Other analyses	17	Report other analyses done—eg analyses of subgroups and interactions, and sensitivity analyses	12-13
**Discussion**			
Key results	18	Summarise key results with reference to study objectives	14
Limitations	19	Discuss limitations of the study, taking into account sources of potential bias or imprecision. Discuss both direction and magnitude of any potential bias	16
Interpretation	20	Give a cautious overall interpretation of results considering objectives, limitations, multiplicity of analyses, results from similar studies, and other relevant evidence	14-16
Generalisability	21	Discuss the generalisability (external validity) of the study results	14
**Other information**			
Funding	22	Give the source of funding and the role of the funders for the present study and, if applicable, for the original study on which the present article is based	18

*Give information separately for cases and controls in case-control studies and, if applicable, for exposed and unexposed groups in cohort and cross-sectional studies.

Note: An Explanation and Elaboration article discusses each checklist item and gives methodological background and published examples of transparent reporting. The STROBE checklist is best used in conjunction with this article (freely available on the Web sites of PLoS Medicine at http://www.plosmedicine.org/, Annals of Internal Medicine at http://www.annals.org/, and Epidemiology at http://www.epidem.com/). Information on the STROBE Initiative is available at www.strobe-statement.org.

For peer review only
